# High throughput sequencing reveals novel and abiotic stress-regulated microRNAs in the inflorescences of rice

**DOI:** 10.1186/1471-2229-12-132

**Published:** 2012-08-03

**Authors:** Blanca E Barrera-Figueroa, Lei Gao, Zhigang Wu, Xuefeng Zhou, Jianhua Zhu, Hailing Jin, Renyi Liu, Jian-Kang Zhu

**Affiliations:** 1 Department of Botany and Plant Sciences and Institute for Integrative Genome Biology, University of California, Riverside, CA, 92521, USA; 2 Instituto de Biotecnología, Universidad del Papaloapan, Tuxtepec, Oaxaca, 38601, Mexico; 3 Department of Computer Science and Engineering, Washington University in St. Louis, St. Louis, MO, 63130, USA; 4 Department of Plant Science and Landscape Architecture, University of Maryland, College Park, MD, 20742, USA; 5 Department of Plant Pathology and Microbiology, Center for Plant Cell Biology and Institute for Integrative Genome Biology, University of California, Riverside, CA, 92521, USA; 6 Department of Horticulture and Landscape Architecture, Purdue University, West Lafayette, IN, 47907, USA

## Abstract

**Background:**

MicroRNAs (miRNAs) are small RNA molecules that play important regulatory roles in plant development and stress responses. Identification of stress-regulated miRNAs is crucial for understanding how plants respond to environmental stimuli. Abiotic stresses are one of the major factors that limit crop growth and yield. Whereas abiotic stress-regulated miRNAs have been identified in vegetative tissues in several plants, they are not well studied in reproductive tissues such as inflorescences.

**Results:**

We used Illumina deep sequencing technology to sequence four small RNA libraries that were constructed from the inflorescences of rice plants that were grown under control condition and drought, cold, or salt stress. We identified 227 miRNAs that belong to 127 families, including 70 miRNAs that are not present in the miRBase. We validated 62 miRNAs (including 10 novel miRNAs) using published small RNA expression data in *DCL1*, *DCL3*, and *RDR2* RNAi lines and confirmed 210 targets from 86 miRNAs using published degradome data. By comparing the expression levels of miRNAs, we identified 18, 15, and 10 miRNAs that were regulated by drought, cold and salt stress conditions, respectively. In addition, we identified 80 candidate miRNAs that originated from transposable elements or repeats, especially miniature inverted-repeat elements (MITEs).

**Conclusion:**

We discovered novel miRNAs and stress-regulated miRNAs that may play critical roles in stress response in rice inflorescences. Transposable elements or repeats, especially MITEs, are rich sources for miRNA origination.

## Background

Endogenous small RNAs (sRNAs) are 20–30 nt RNA molecules that modulate gene expression at the transcriptional and posttranscriptional levels and have roles in developmental and physiological processes in eukaryotic organisms [1-3]. In plants, sRNAs are classified into microRNAs (miRNAs) and small interfering RNAs (siRNAs), according to their biogenesis and precursor structures [4-9]. siRNAs are derived from double stranded RNA precursors and can be further divided into heterochromatic siRNAs (hc-siRNAs), trans-acting siRNAs (ta-siRNAs), long siRNAs (lsiRNAs), and natural antisense transcripts-derived siRNAs (nat-siRNAs). hc-siRNAs are involved in DNA methylation and histone modifications that lead to silencing of target genes at the transcriptional level [10]. ta-siRNAs, lsiRNAs, and nat-siRNAs act at the posttranscriptional level, guiding mRNA cleavage, degradation, or translational repression of target genes. *Arabidopsis* ta-siRNAs are phased sRNAs generated from a primary transcript that is targeted by a miRNA. The product of this cleavage is then converted to double stranded RNA by RNA-dependent RNA polymerase 6 (RDR6) and processed by Dicer like protein 4 (DCL4) to produce ta-siRNAs [11]. *Arabidopsis* lsiRNAs are DCL1-dependent sRNAs of 30–40 nt in length, which mediate mRNA decapping and degradation [12]. nat-siRNAs are generated from co-expressed cis-antisense overlapping genes. The transcripts of overlapping genes may hybridize to form double-stranded RNAs and be processed by DCL proteins into sRNAs that target the antisense gene for regulation [13-15].

miRNAs are distinguished from siRNAs since they are transcribed into a single-stranded pri-miRNA by RNA polymerase II, which folds into a stable, usually imperfect, hairpin structure [16]. This structure is then processed by DCL1 to produce ~21 nt, double-stranded RNA duplex. The duplex is exported into the cytoplasm by HASTY and methylated at the 3’ end by HEN1 [17]. One strand functions as the mature miRNA and is incorporated into the RNA-Induced Silencing Complex (RISC) to target mRNAs for cleavage in a sequence-specific manner. The other strand, called miRNA*, is usually degraded, although some miRNA*s were found to be functional under certain conditions [18]. Plant miRNAs recognize their targets through near-perfect complementarity to direct RISC-mediated cleavage, although in some cases translational inhibition and DNA methylation can be the mode of action of miRNA-mediated gene silencing [19-21].

Several studies have demonstrated that miRNAs play important roles in the responses to biotic and abiotic stimuli [22-24]. Abiotic stress-regulated miRNAs were first investigated in *Arabidopsis*. Sunkar and Zhu [25] showed that miR393, miR402, miR397b, and miR319c were induced by at least one of the treatments including drought, cold, salt and ABA, whereas miR398 was downregulated. Further study showed that miR398 mediates the post-transcriptional induction of two *superoxide dismutase* genes involved in the first line of defense against toxic superoxide radicals and is also downregulated by oxidative stress in *Arabidopsis*[26]. Also in *Arabidopsis*, miR169 is downregulated by drought through an ABA-dependent pathway to control the expression of the NFYA5 transcription factor, which mediates tolerance to drought [27].

To discover stress-regulated miRNAs, it is necessary to compare the expression of miRNAs in plants grown under normal and stress-treated conditions. This was achieved by Northern blot analyses when digital expression analysis was not effective because traditional sequencing technology provided only very low coverage [25]. With the application of next-generation sequencing and microarrays, it became much easier and cost-effective to perform genome-wide expression profiling to identify stress-regulated miRNAs. As a result, discovery of stress-regulated miRNAs has expanded from the model dicot *Arabidopsis* to model monocot rice and other non-model plants, and many more stress-regulated miRNAs were found [28-35].

Rice (*Oryza sativa*) is a staple crop that is cultivated worldwide and constitutes a primary source of human food. Besides its high agricultural importance, rice is a model monocot with a small genome and excellent genomic resources. Recently extensive efforts have been devoted to the discovery of novel miRNAs, as well as the analysis of miRNAs in stress responses in rice [28,29,36-44]. miRNAs that are regulated by various stresses were identified. For example, a genome-wide study conducted across different developmental stages of rice revealed that 16 miRNAs, including miR156, miR159 and miR168, were downregulated by drought stress, while 14 miRNAs, such as miR169, miR319 and miR395, were upregulated [42].

Most of previous studies on miRNAs that are regulated by abiotic stresses in rice have been focused on early growth stages [36,41,44-46]. However, the onset of abiotic stresses during reproductive stages of rice can dramatically compromise seed production. Seed development requires a series of molecular events that are finely regulated at the transcriptional and posttranscriptional levels [47] and it has been recently demonstrated that miRNAs are involved in those processes [39]. Therefore, there is a need to expand our knowledge on miRNA expression in reproductive tissues under abiotic stresses. In this study we set to identify miRNAs that were involved in the responses to abiotic stresses in rice inflorescences. We applied high-throughput sequencing and bioinformatic analysis to small RNA populations derived from rice inflorescences under control, as well as drought, cold, and salt stresses. We identified 227 miRNAs, including 70 candidate miRNAs that are not in the miRBase. Using stringent criteria, we identified 18, 15, and 10 miRNAs that were differentially regulated by drought, cold, and salt stress, respectively. We validated 62 miRNAs using published small RNA data from *DCL1DCL3RDR2* RNAi knockdown lines and confirmed 210 miRNA targets using published degradome data. We also identified 80 miRNAs that appear to have originated from transposable elements and repeats.

## Results and discussion

### Identification of miRNAs

We constructed and sequenced four small RNA libraries using the inflorescences of rice plants that grew under control (untreated) and three abiotic stress treatments. After quality control and adaptor removal, we obtained 5328145, 4186380, 3524691 and 3992166 high quality clean reads from control, drought, cold and salt libraries, respectively (Additional file 1). We removed around 27% of clean reads from each library that matched rice repeats and known rRNAs, tRNAs, snoRNAs, snRNAs, and then mapped the rest of small RNA reads to the rice whole genome sequence. Using an in-house miRNA prediction pipeline that was built according to the updated annotation criteria for plant miRNAs [48,49], we predicted 227 miRNAs that were classified into 127 families (details of predicted miRNAs are included in Additional file 2).

Because rice is an important crop and is the model species for monocotyledons, it has been subject to substantial effort for miRNA discovery. As a result, rice has 491 registered miRNAs in the miRBase [50] (release 17), which is the most for any plant species. We compared the genomic locations and mature miRNAs of our predicted miRNAs to those of known rice miRNAs in the miRBase and found that 145 predicted miRNAs (64 families) had already been included in the database, 12 predicted miRNAs were new homologs of 7 known miRNA families (allowing mature miRNAs to have up to 2 mismatches), and 70 miRNAs (62 families) were new miRNA candidates (Additional file 2; Predicted secondary structures of new miRNA candidates are provided in Additional file 3 and distributions and abundances of all sRNAs that are mapped to each precursor are provided in Additional file 4). Among these new miRNA candidates, osa-cand006, osa-cand021, osa-cand027, osa-cand032, and osa-cand036 were included in release 18 of the miRBase as osa-miR5159, osa-miR2863c, osa-miR5485, osa-5150-3p, and osa-miR5337, respectively.

Among 145 miRNA precursors that match registered rice miRNAs in the miRBase, 81 precursors have identical predicted mature miRNAs to those in the miRBase, 40 have predicted mature miRNAs that are highly similar but not identical, and 24 have a mature miRNA that is different from the registered mature miRNA (Additional file 5). For each predicted miRNA precursor, a small RNA is usually chosen as the mature miRNA if it has the highest abundance among all reads that are mapped to the precursor. In some cases, small RNA with highest expression was not chosen because it did not reside in a hairpin structure that meets the stringent structural requirements for miRNA annotation [48,49]. Because the cleavage of miRNA precursor into miRNA/miRNA* duplex is imprecise at some level [51], generating overlapping but not identical small RNAs with different abundances over the hairpin region, the choice for mature miRNAs may not be clear cut, especially when number of mapped reads is low. Low coverage can be caused by low sequencing depth from early sequencing technologies such as 454 pyrosequencing, comparing to Illumina sequencing that was used in this study. Low coverage can also be caused by the low expression level of some miRNAs in the chosen tissue and under the chosen growth condition. In addition, a miRNA* might have higher abundance than the miRNA in some cases [18,39].

### Validation of predicted miRNAs

Based on their biogenesis pathway, rice miRNAs can be grouped into two classes. The first class is canonical miRNAs (cmiRNAs), which are usually 21nt in length, cleaved by DCL1, and sorted into Argonaut 1 (AGO1) family proteins [21,52]. The other class is long miRNAs (lmiRNAs), which are usually 24nt in length, cleaved by DCL3, and sorted into AGO4 family proteins [21]. Both classes of miRNAs do not require RDR2, which is a critical component in the siRNA biogenesis pathway. Therefore, the expression of authentic miRNAs would be significantly reduced in either *dcl1* or *dcl3* knockdown lines, but not in the *rdr2* knockdown line.

In order to validate our 227 predicted miRNAs, we compared the abundances of their mature miRNAs in the published small RNA sequencing data from the wild type (WT) and three RNAi knockdown lines (*dcl3a-17rdr2-2*, and *DCL1IR-2*) [21], as well as small RNAs that were pulled down with Argonaut proteins AGO1a, AGO1b, AGO1c, AGO4a, AGO4b, and AGO16 [21,53]. Using the criteria described in the Methods, we were able to confirm 46 canonical miRNAs that had significantly reduced expression in *DCL1IR-2*, including six novel miRNAs (osa-cand001, osa-cand039, osa-cand053, osa-cand056, osa-cand059, and osa-cand060). We also confirmed 16 long miRNAs that had significantly reduced expression in *dcl3a-17*, including four novel miRNAs (osa-cand020, osa-cand021, osa-cand032, and osa-cand054). Five more miRNAs (osa-cand017, osa-cand058, osa-miR1862, osa-miR1862d, and osa-miR440) narrowly missed the list of confirmed lmiRNAs because their expression level in *rdr2-2* was slightly less than 50% of that in WT (Additional file 6). As expected, whereas confirmed cmiRNAs have a typical length of 21nt and are predominantly associated with AGO1 proteins, lmiRNAs have a typical length of 24nt and are exclusively associated with AGO4 or AGO16 proteins (Additional file 6).

### Prediction and confirmation of miRNA targets

Because plant miRNAs have near perfect complementarity to their targets, computational prediction has been an effective way to identifying miRNA targets. Using the procedure described in the Methods, we searched rice annotated cDNAs and were able to predict targets for 170 (75%) miRNAs (Additional file 2). Deep sequencing of mRNA cleavage products (degradome sequencing) provides a means for confirmation of miRNA targets [54,55]. To confirm miRNA targets that were predicted in this study, we searched the two published degradome datasets from *Oryza sativa* ssp. japonica using the CleaveLand software [56]. We were able to confirm 210 targets for 86 miRNAs (Additional file 7), including 154 targets that were previously verified [46,53], mostly for known miRNAs. We also confirmed 20 targets for 12 novel miRNAs. For instance, osa-cand041 targets a putative inorganic phosphate transporter, osa-cand046 targets a putative amino acid transporter, and osa-cand026 targets a putative ATP-dependent RNA helicase. We were also able to confirm some targets for new miRNA homologs and for known miRNAs for the first time (Additional file 7).

Because many miRNAs and target genes show tissue- or growth condition-specific expression patterns, our ability to verify predicted targets depends highly on the treatments and tissues used to construct the degradome libraries. As more degradome data from a variety of tissues and treatments become available, it will be possible to verify targets for more miRNAs.

### Abiotic stress-regulated miRNAs

The deep coverage of mature miRNAs provided by Illumina sequencing allowed us to compare the normalized count of each miRNA in a stressed library to that in the control library to find miRNAs that were induced or downregulated by the stress. Normalized expression level was calculated as the number of mature miRNAs per ten million clean small RNA reads (transcripts per ten million, TPTM). A miRNA was identified as regulated by a particular stress only if the following three conditions were met: (1) normalized expression was at least 100 TPTM in either control or stress library; (2) log2 ratio of normalized expression under stress or control was greater than 1 or less than −1; (3) test for differential expression in stress versus control library according to the Audic and Claverie (1997) [57,58] method was significant (p ≤ 0.01). Applying this stringent set of criteria, we identified 18, 15, and 10 miRNAs that were regulated by drought, cold, and salt stress, respectively (Table 1).

**Table 1:** Abiotic stress-regulated miRNAs identified in rice inflorescences

**MiRNA**	**Family**	*Log2 (Drought/Ctrl)		*Log2 (Cold/Ctrl)		*Log2 (Salt/Ctrl)		**Putative target**
AAUUCACAGGCCCUAUCUUGUG	miR1428	−2.71	↓	−0.82		−0.15		Cytochrome c
CUUGGAUUGAAGGGAGCUCU	miR159	0.03		0.99		−1.57	↓	MYB family transcription factor
UGCCUGGCUCCCUGUAUGCCA	miR160	0.28		0.29		−1.07	↓	Auxin response factor
UGCCUGGCUCCCUGAAUGCCA	miR160	1.40	↑	0.60		−0.37		Auxin response factor
GGAAUGUUGUCUGGCUCGGGG	miR166	−1.69	↓	−0.65		−0.96		START domain containing protein
GGAAUGUUGUCUGGUCCGAGA	miR166	−2.68	↓	0.08		−0.63		START domain containing protein
UGAAGCUGCCAGCAUGAUCUA	miR167	1.02	↑	0.81		−0.79		Auxin response factor
UAUGCGUAAGACGGAUUCGUA	miR1856	0.72		1.29	↑	0.95		
GAGGGAUUUUGCGGGAAUUUCACG	miR1866	−4.67	↓	−7.00	↓	−1.49	↓	Hypotethical protein
UUCAGUUUCCUCUAAUAUCUCG	miR2275d	1.46	↑	1.62	↑	0.09		
CUUGUUUUUCUCCAAUAUCUCA	miR2275e	1.37	↑	1.83	↑	0.15		
AUUGUUUUUCUCCAAUAUCUCA	miR2275c	1.32	↑	1.32	↑	−0.02		
UAUUUUAGUUUCUAUGGUCAC	miR2871	1.12	↑	1.96	↑	1.56	↑	Glycosyltransferase family protein
UUGGACUGAAGGGUGCUCCC	miR319	−0.46		−0.09		−1.46	↓	TCP family transcription factor
UCCAAAGGGAUCGCAUUGAUC	miR393	1.26	↑	0.98		0.55		F-box domain and LRR containing protein
UCAGUGCAAUCCCUUUGGAAU	miR393	0.99		1.08	↑	0.21		MYB family transcription factor
UUGGCAUUCUGUCCACCUCC	miR394	0.21		1.19	↑	−2.94	↓	F-box domain containing protein
UUCCACAGCUUUCUUGAACUG	miR396	0.69		1.09	↑	0.34		Growth regulating factor
UUCCACAGCUUUCUUGAACUU	miR396	1.03	↑	2.09	↑	−0.14		Growth regulating factor
UCCACAGGCUUUCUUGAACUG	miR396	1.17	↑	1.32	↑	0.06		Growth-regulating factor
UGGAAGGGGCAUGCAGAGGAG	miR528	−0.70		−0.46		−1.39	↓	Plastocyanin-like domain containing protein
AGAAGAGAGAGAGUACAGCCU	miR529	1.41	↑	1.35	↑	0.79		SBP-box gene family
AGGUGCAGAGGCAGAUGCAAC	miR530-3p	−2.23	↓	1.09	↑	−2.06	↓	Hairpin-induced protein 1 domain containing protein
UUGCUCUGAUACCAAUUGUCGG	osa-cand027	2.35	↑	0.60		−0.72		
GAAGCUGCAGCUGUCAGAAGCUCC	osa-cand032	−0.01		−0.64		−1.18	↓	
AAUGGCUUGUCUUGUUUUGUGUGC	osa-cand042	−0.62		−2.82	↓	−1.24	↓	
UACAACUUCUUGUUGAUGGAAACU	osa-cand052	−3.66	↓	−7.49	↓	−0.92		
UAAAUGGAGAGAACGAAAGAG	osa-cand056	1.25	↑	0.96		0.32		

*Log2 ratio of normalized miRNA expression in stress and control libraries. Ctrl: control condition; ↑ and ↓: up- and downregulated in stress, respectively.

We observed an apparent difference in how a stress would change the expression of miRNAs. Whereas the majority of stress-regulated miRNAs were upregulated by drought (12 out of 18) or cold stress (12 out of 15), most salt-regulated miRNAs were downregulated (9 out of 10). Whereas 16 miRNAs were regulated with only one of the three abiotic stresses examined, 12 miRNAs were regulated by two or three stresses (Table 1), indicating that they might be involved in a pathway that is shared in the response to different stresses. For example, miR2871b was induced by all three abiotic stresses examined in this study.

**Figure 1:**
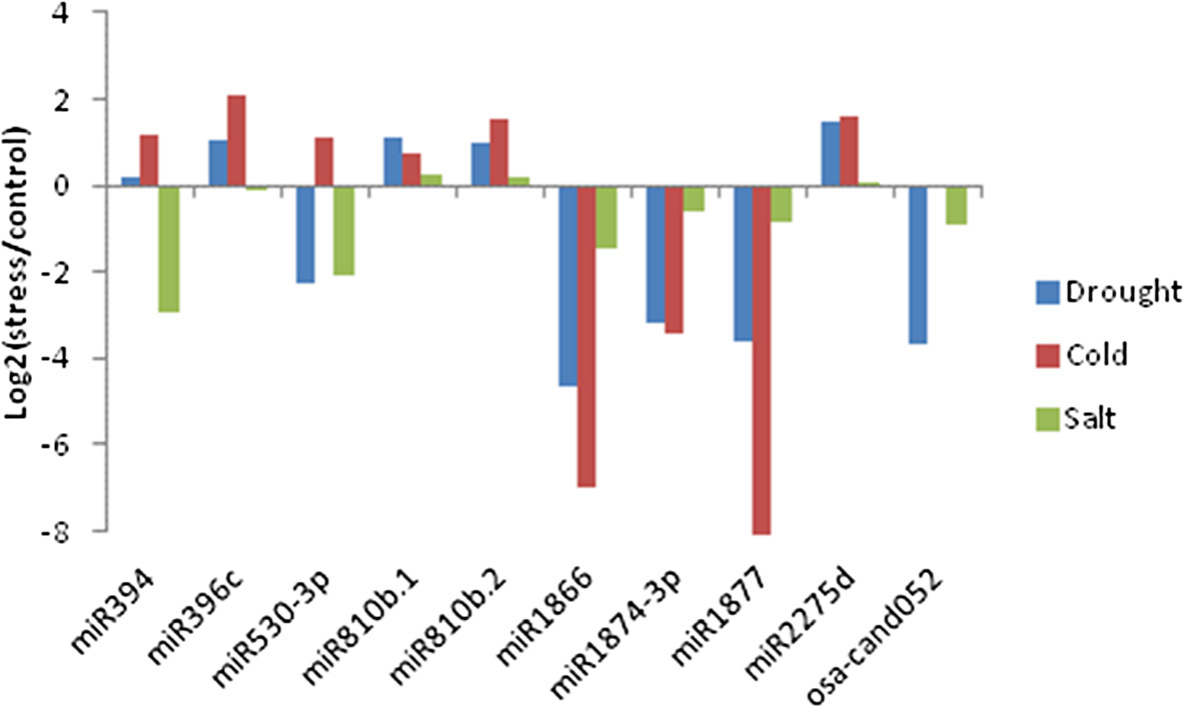
**Relative expression level of selected stress-regulated miRNAs in rice inflorescences.** Log_2_ was calculated for the ratio of normalized miRNA counts in each library to that in the control library.

**Figure 2:**
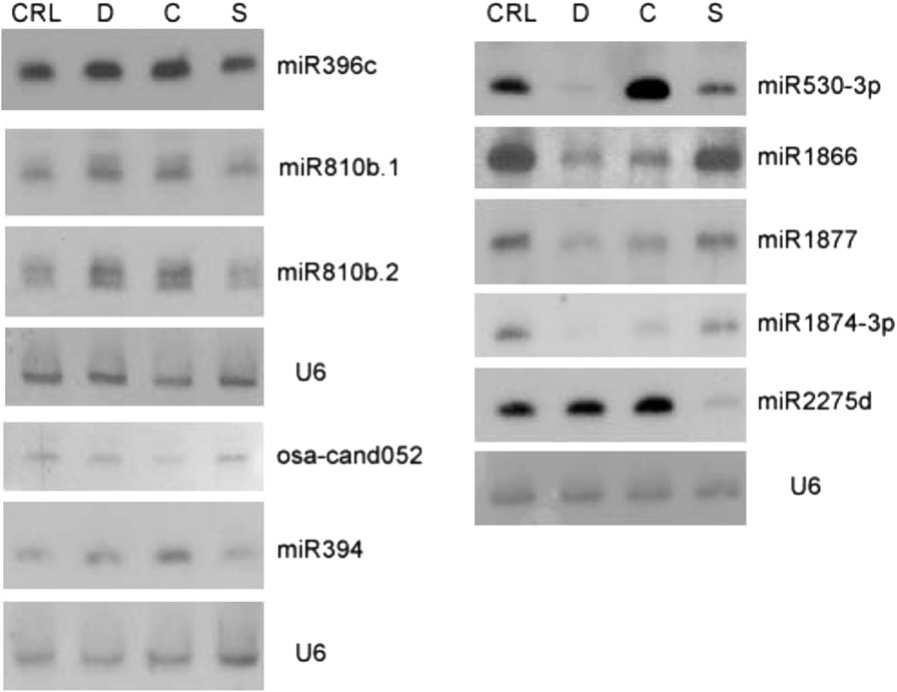
**Northern blot analysis of selected stress-regulated miRNAs in rice inflorescences.** Each sample contains 40 μg of total RNAs from inflorescences of plants that were treated with drought (D), cold (C) and salt (S) stress, or control (CRL) conditions, respectively. U6 was used as control of equal loading as shown at the bottom of each hybridization set.

Among the miRNAs that were identified as stress-regulated miRNAs here, some miRNA families have been previously found to be regulated by certain stress, either in rice or in other plants. For example, miR167, miR393, miR396, miR529 have been shown to be regulated by drought stress [25,42,59], miR393 and miR396 were shown to be regulated by cold stress [25,42,59], and miR159, miR160, miR319, miR394, miR528, and miR530 were shown to be regulated by salt stress [25,29,59-61]. To the best of our knowledge, some miRNAs were found to be regulated by certain stress for the first time in this study. This list includes miR1428, miR160, miR1866, miR2275, miR2871, and miR530 that were regulated by drought stress, miR1866, miR2275, miR2871, miR394, and miR529 that were regulated by cold stress, and miR1866 by salt stress. In addition, we identified five novel miRNA candidates (osa-cand027, osa-cand032, osa-cand042, osa-cand-052, and osa-cand056) that were regulated by at least one of the three abiotic stresses in rice inflorescences (Table 1). Among these stress-regulated miRNAs, we randomly chose five miRNAs to confirm their expression patterns with Northern blot assays. In general, the differential expression patterns of miRNAs based on cloning frequencies in small RNA sequencing libraries (Figure 1) were consistent with Northern blot results (Figure 2), although the strong upregulation of miRNA396 was only partially reflected in Northern blots.

Targets for stress-regulated miRNAs were predicted and confirmed based on degradome data analysis. As shown in Table 1, targets of stress-regulated miRNAs have diverse functions, mainly as transcriptional factors such as MYB, auxin responsive factors and proteins with F-box domains. Since miR2871 was upregulated in all stress conditions tested, it is very likely that its target, a glycosyltransferase family protein, is downregulated. Therefore, negative regulation of glycosylation processes may be a common mechanism to respond to a variety of abiotic stresses. On the other hand, miR396 regulates a family of growth factors in rice [42]. Its upregulation in our drought and cold stress libraries suggests the downregulation of growth factors, perhaps to redirect resources to other parts of the plant in response to drought. This confirms that growth regulation is a mechanism highly sensitive to abiotic stresses.

Three miRNAs, miR394, miR530-3p and miR2275d were upregulated by cold stress (Figure 1). The predicted targets of miR394 are F-Box proteins of diverse functions [46,62]. Interestingly, miR530-3p was strongly accumulated in cold treated plants, while it was downregulated by drought and salt stresses as predicted by quantitative analysis and confirmed in Northern blots (Table 1, Figure 2). This suggests a high level of specialization of miRNAs to respond to different abiotic stresses. The expression patterns suggest that some elements need to be regulated by drought and salt treatments, while the same elements are regulated in an opposite way under cold stress to regulate a cold specific mechanism.

We used very stringent criteria for determining stress-regulated miRNAs. While it helps us reduce false positives, some true stress-regulated miRNAs may be missed. For example, miR169 has been previously found to be downregulated in drought-stressed *Arabidopsis*[27] and salt-stressed *Nicotiana*[60], but induced by drought in *Nicotiana*[60] and rice [45], and by UV irradiation in *Populus tremula*[63]. miR169b and miR169c were highly expressed in rice inflorescences and were apparently upregulated by drought and cold stresses (Additional file 2). However, the change of expression was a little bit less than 2-fold in both cases and miR169 was not identified as stress-regulated miRNA based on our criteria.

The results of this work confirm that some miRNAs may be involved in response to several abiotic stresses, while others seem to be specific to an individual stress. Differences in expression patterns could also be an effect of the nature and severity of individual stress and the level of impact that it has on the tissue under study. For example, we observed that salt treated plants showed clear symptoms of stress on the leaves, e.g. the rolling of old leaves, while inflorescences did not appear to be affected since the plants were able to recover and produced normal amount of seeds after they were irrigated with NaCl-free nutrient solution. In contrast, cold and drought stress caused apparent damage to the inflorescences. Even though plants were able to recover and continued growing after stress treatments were removed, both development of new inflorescences and production of seeds were reduced, most likely due to stress-induced sterility or permanent damage to floral structures.

It is interesting that, in general, we did not find dramatic changes in expression of miRNAs in response to abiotic stresses and only a small fraction of miRNAs showed some level of regulation. Overall, the slight changes observed in the expression of miRNAs points to the existence of a fine-tuning mechanism rather than a dramatic control of expression exerted by miRNAs under drought, cold and salt stress in rice inflorescences. This fine-tuning mechanism may be important in plants to regulate gene expression without impacting negatively growth and development.

### Other known miRNAs that were regulated by stresses

Because we used a stringent set of criteria on miRNA structure and expression pattern [48] for miRNA prediction and identification, it is possible that some real miRNAs were left behind, including some stress-regulated miRNAs. In order to find additional known miRNAs that are regulated by abiotic stresses, we used the mature miRNAs from the miRBase to query our small RNA reads from each library and compared their frequencies. We indeed found a few known miRNAs that appear to be regulated by abiotic stresses (Table 2). We confirmed their expression using Northern blot assays (Figure 2) and the hybridization signals were in general consistent with log2 ratios of mature miRNA counts (Figure 1).

**Table 2:** Transposon- or repeat-derived miRNAs and other known miRNAs that were regulated with abiotic stresses

**MiRNA**	**Family**	*Log2 (Drought/Ctrl)		*Log2 (Cold/Ctrl)		*Log2 (Salt/Ctrl)		**Putative target**
UGGAUGUGACAUACUCUAGUA	osa-cand066	0.72		1.27	↑	0.42		LTPL8 - Protease inhibitor
UGGGAUACUGAUGUCGAGGUCGAG	osa-cand084	−1.25	↓	−2.33	↓	−1.12	↓	Transposon protein
AUAAGACGGACAGUCAAAGUUGGA	osa-cand085	−0.68		−0.71		−1.26	↓	Expressed protein
AAUGUAUGACGCUGUUGACUUUUA	miR1884	−1.38	↓	−0.77		−0.15		AAA ATPase
AUGAAUGUGGGCAAUGCUAGAAAG	miR809	−0.59		−1.54	↓	−0.77		Glutaredoxin 2, putative
AUGAAUGUGAGAAAUGCUAGAAUG	miR809	−0.62		0.31		−2.08	↓	Glutaredoxin subgroup II
UAUGAAUGUGGGCAAUGCUAGAAA	miR809	−1.35	↓	−0.88		−0.71		PPR repeat containing protein
TGAACACCGATATGCGTCATC	miR810b.1	1.10	↑	0.76		0.25		Unknown
AAGTGATTTAATTATGCCGTT	miR810b.2	0.96		1.51	↑	0.17		Unknown
TATGGATGGAGGTGTAACCCGATG	miR1874-3p	−3.20	↓	−3.40	↓	−0.62		Unknown
AGATGACATGTGAATGATGAGGGG	miR1877	−3.61	↓	−8.34	↓	−0.83		Unknown

*Log2 ratio of normalized miRNA expression in stress and control libraries. Ctrl: control condition; ↑ and ↓: up- and downregulated in stress, respectively.

### Transposon- and Repeat-derived miRNAs

In order to reduce false positives in miRNA discovery, it is a general practice to remove small RNAs that are highly similar to known transposable elements and repetitive sequences from consideration. In our first round of miRNA prediction, we did not consider small RNA reads as potential mature miRNAs if they match known transposons or repeats, or have a copy number higher than 20 in the genome. However, recent studies have shown that miRNAs could have originated from transposons or repeats [64,65]. Rice genome is enriched with miniature inverted-repeat transposable elements (MITEs) [66] and other inverted repeats that once transcribed, may form hairpin structures and be cleaved into miRNAs by DCL1 or DCL3. To identify miRNAs that may have been derived from transposons and repeats, we relaxed the requirements in the first round of miRNA prediction and used small RNA reads that did have a match (alignment length ≥ 80% of the length of the small RNA, and identity ≥ 80%) in the rice repeats database (ftp://ftp.plantbiology.msu.edu/pub/data/TIGR_Plant_Repeats) or Repbase [67] or have a copy number (allowing up to 2 mismatches) higher than 20 in the genome, as anchor sequences for miRNA prediction. We predicted 424 potential miRNA precursors (Additional file 8). These precursors met all the criteria for miRNA annotation, including precise cleavage and strand bias in expression.

To further check the authenticity of these potential miRNAs, we again examined their expression in the *DCL1IR-2*, *dcl3a-17*, and *rdr2-2* RNAi lines. Using the same set of criteria for miRNA validation as stated earlier, we found that 29 miRNAs were potentially generated by DCL1 and 51 miRNAs that were generated by DCL3. All these 80 miRNAs did not depend on RDR2 and were sorted into AGO1 or AGO4 family proteins, respectively, as expected. We inspected the types of transposable elements or repeats from which these miRNAs have originated and found that 53 (66%) miRNAs were derived from MITEs, 11 from En/Spm DNA transposons, 7 from non LTR retrotransposon LINE1, and 9 from unclassified repeats (Additional file 9). Compared to the copy number abundance of different classes of TEs in the genome, MITEs are apparently enriched in TEs from which miRNAs were derived (Additional file 10). *Stowaway* and *adh* were the two main types of MITEs that contributed to miRNA origination, accounting for 37 and 11 miRNAs, respectively. Both 21nt cmiRNAs and 24nt lmiRNAs originated from the same MITE families such as *Stowaway* and *adh*, indicating that once MITEs were transcribed, they could fold into hairpin structures and be cleaved by either DCL1 or DCL3 proteins. This list contains some known miRNAs in the miRBase, including 7 miR166 family miRNAs derived from a LINE element, two miR169 family miRNAs derived from En/Spm transposons, and 19 miR809 family miRNAs derived from MITEs (Additional file 9).

We were able to predict at least one target for 78 TE/repeat derived miRNAs, indicating these miRNAs do have biological functions (Additional file 9). Among the targets predicted with high confidence, there are proteins such as 3-ketoacyl-CoA synthases that are targeted by miR809 and are involved in the biosynthesis of cuticular wax [68]. Other TE/repeat derived miRNAs appear to target genes that encode transcriptional activators (osa-cand064, osa-cand076, osa-cand079, and miR809), proteins involved in signaling cascades (osa-cand071 and osa-cand077), and proteins that may be involved in regulation of transposon and retrotransposon activity (miR166, osa-cand063, and osa-cand084). These results suggest a potential role of transposon- and repeat-derived miRNAs in important processes in plant biology.

Using the same set of criteria for finding stress-regulated miRNAs, we found that osa-cand066 was upregulated by cold, osa-cand084 downregulated by all three stresses, osa-cand085 downregulated by salt, miR1884 downregulated by drought, some miR809 family miRNAs downregulated by drought or cold (Table 2Additional file 9). It is remarkable that members of the miR809 family are predicted to target F-box-containing proteins and glutarredoxin. Many F-box proteins have been found in the response to drought stress [69], whereas glutarredoxins are proteins involved in the defense against oxidative stress [70]. It is possible that downregulation of miR809 triggers the accumulation of these proteins in a mechanism to cope with the abiotic stresses.

## Conclusions

High-throughput sequencing is a cost-effective approach for identification of novel miRNAs in the inflorescences of rice. Deep sequencing also allows for comparison of miRNA expression in different growth conditions and for identification of stress-regulated miRNAs. Our results suggest that miRNAs play important roles in rice in response to abiotic stresses not only in vegetative tissues as shown in previous studies, but also in reproductive tissues. Further functional analysis of stress-regulated miRNAs and their targets will allow us to dissect the complex miRNA-mediated pathways and networks in plant stress responses.

## Methods

### Plant materials and stress treatments

Rice (*Oryza sativa* spp. *japonica* cv. Nipponbare) plants were grown in a greenhouse at 28°C, 13 h light until 90 day old and were then randomly divided into four groups. One group was used as untreated control, and other three groups were treated with drought (water withholding for 3 days), cold (5°C for 24 hours) or salt (400 mM NaCl for 24 hours) stresses, respectively. After control and stress treatments were applied, mature inflorescences of 14–17 cm in length, in stage In 9 were collected and stored at −80°C until RNA extraction. Inflorescence tissues, composed by rachis, branches and spikelets from 10 plants per treatment, were used for total RNA extraction with Trizol (Invitrogen). There were no seeds in the collected tissues. The treatments used in this work were set to induce an intermediate level of stress, based on the development of clear, typical stress symptoms in rice, such as leaf rolling and wilting, while plants were still able to recover and resume growth if they were transferred from the stress treatments to normal growth condition.

### Small RNA library construction and deep sequencing

Small RNA libraries were constructed following a standard method [25] with some modifications [71]. In brief, total RNA was run in 15% denaturing polyacrylamide gel and the 18-30nt small RNA fraction was excised and eluted. After an adaptor was added to the 3’ end and another to the 5’ end, small RNA constructs were reverse-transcribed and amplified by PCR for 15 cycles. The products were sequenced with an Illumina Genome Analyzer at the UC Riverside Core Facilities.

### Prediction of miRNAs

Raw reads from four small RNA libraries were first processed to obtain clean reads. Only raw reads that contained clear adaptor sequence were considered. After adaptor sequence was removed, clean reads of at least 18nt long were clustered into unique reads. We first removed reads that match known rice repeats (ftp://ftp.plantbiology.msu.edu/pub/data/TIGR_Plant_Repeats), rRNAs, tRNAs, snRNAs, and snoRNAs and then used an in-house miRNA prediction pipeline to predict miRNAs [49]. We mapped unique reads to the rice genome assembly (version 6.1, ftp://ftp.plantbiology.msu.edu) with SOAP2 [72], requiring perfect matches. To ensure that our miRNA candidates did not come from highly repetitive sequences, we removed unique reads that had more than 20 hits (allowing up to 2 mismatches) in the rice genome. Mapped reads with a minimum count of 10 were used as anchor sequences to extract surrounding DNA segments. These segments had one end at 20nt away from the mapping location on one side and extended across the anchor sequence for 100 to 300nt with a step size of 20nt. Extracted DNA segments were folded into secondary structures using UNAFold [73]. A segment was considered a valid miRNA candidate if its secondary structure met the following criteria according to Meyers et al. [48]: (1) free energy is at most −35 kcal/mol; (2) number of mismatches between putative miRNA and miRNA* is 4 or less; (3) number of asymmetrical bulges in the stem region is no more than 1 and if exist, its size is 2 or less; (4) strand bias ― the ratio of small RNA reads that map to the positive and negative strands of the DNA segment is 5:1 or more (5) precise cleavage ― reads that map to the miRNA and miRNA* regions (defined as miRNA or miRNA* plus 2nt on the 5’ and 3’ ends) account for at least 75% of reads that map to the segment. If more than one segment from the same locus met the above criteria, we chose the segment with highest putative miRNA counts, lowest free energy, or shortest length as the candidate miRNA precursor.

We classified predicted miRNAs into families by first comparing mature miRNAs with themselves using the ssearch35 program in the FASTA package (version 3.5) [74]. We then used a single-linkage algorithm to put homologous miRNAs (up to two mismatches were allowed) into clusters. miRNAs from each cluster were then compared to the known plant mature miRNAs in the miRBase (Release 17) [75] using ssearch35. If a cluster had a homologous known miRNA (allowing up to two mismatches in the alignment), the family number of the known miRNA was assigned to the cluster, otherwise the cluster was annotated as a new family.

### Validation of predicted miRNAs

Mature miRNA sequences of predicted miRNAs were searched in the published small RNA sequencing data and their abundances were compared in the wild type (WT) and three RNAi knockdown lines (*dcl3a-17rdr2-2*, and *DCL1IR-2*) [21], as well as small RNAs that were pulled down with Argonaut proteins AGO1a, AGO1b, AGO1c, AGO4a, AGO4b, and AGO16 [21,53]. A miRNA was considered validated if it met four conditions: (1) its normalized expression level (transcripts per ten million, TPTM) was at least 50 in one of the four libraries (WT, *dcl3a-17rdr2-2*, and *DCL1IR-2*); (2) the ratio of normalized expression in *dcl3a-17* or *DCL1IR-2* versus WT was less than 0.5; (3) the ratio of normalized expression in *dcl3a-17* or *DCL1IR-2* versus *rdr2-2* was also less than 0.5; (4) the ratio of normalized expression in *rdr2-2* versus WT was greater than 0.5.

### Prediction of miRNA targets

We used the predicted mature miRNAs as query to search the annotated rice cDNAs with miRanda [76]. We scored the alignments between miRNAs and potential targets using a position-dependent, mispair penalty system [11,50,77]. Each alignment was divided into two regions: a core region that included positions 2–13 from the 5’ end of the miRNA, and a general region that contained other positions. In the general region, a penalty score of 1 was given to a mismatch or a single-nucleotide bulge or gap, and 0.5 to a G:U pair. Penalty scores were doubled in the core region. A gene was considered a valid target if the alignment between miRNA and target met two conditions: (1) the penalty score is 4 or less; (2) total number of bulges and gaps is less than 2. Predicted miRNA-targets were further validated by searching two public degradome datasets from *Oryza sativa* ssp. japonica [46,53] using the CleaveLand software [56] with default parameters.

### Northern blot assays

Northern blot assays were performed to confirm a selected set of stress-regulated miRNAs. Briefly, 40 μg of total RNA obtained from inflorescences were loaded in 15% denaturing polyacrylamide gels and transferred to a nylon membrane (Hybond NX). The RNA was then fixed to the membrane by chemical cross-linking [78]. Blots were hybridized to radioactive probes complementary to mature miRNAs at 38°C overnight (probe sequences are provided in Additional file 11). Membranes were then washed twice with 2X SSC, 0.1% SDS solution for 5 minutes each, and exposed to X-ray films for autoradiography.

## Accession number

Deep sequencing data from the four small RNA libraries have been deposited in the NCBI/GEO database with accession number GSE26357.

## Authors’ contributions

BEB-F, J-KZ, and RL conceived the study. BEB-F, ZW, and JZ carried out the experiments. BEB-F, LG, ZW, XZ, and RL analyzed the data, LG and RL contributed new analysis tools, BEB-F and RL wrote the paper, J-KZ and HJ edited the paper. All authors read and approved the final manuscript.

## Supplementary Material

Additional file 1 Summary of small RNA sequencing data. Detailed information of preprocessing of small RNA reads from four libraries in rice inflorescences. Click here for file

Additional file 2 **miRNAs that were identified in rice inflorescences.** Detailed information of the predicted rice miRNAs and their targets. Click here for file

Additional file 3 **Predicted secondary structures of the precursors of new miRNA candidates.** Free energy and miRNA ID are on the bottom of each structure. Nucleotides that constitute mature RNAs are drawn in blue. Click here for file

Additional file 4 **Small RNA reads from four libraries that were mapped to the precursors of new candidate miRNAs.** On each map, the first line contains the miRNA ID. The second line contains the miRNA precursor sequence, with mature miRNA region in red. The third line contains the notation of secondary structure with parentheses denoting base-pairing and dots denoting mismatches or bulges. The number on the right is the free energy. Every line starting from line 4 contains the sequence, mapping position, and count of a mapped unique small RNA read. Click here for file

Additional file 5 **Detailed information of known miRNAs that were identified in rice inflorescences.** For each miRNA gene that match a known miRNA gene in the miRBase, precursor sequence is listed first, followed by alignment of mature miRNA sequence identified in this study, and then alignment of mature miRNA sequence that is listed in the miRBase (if it is different). Length of miRNA and normalized expression values in four small RNA libraries are listed on the right side of each mature miRNA. Click here for file

Additional file 6 Validation of predicted miRNAs with published small RNA data from DCL1, DCL3, RDR2 RNAi lines and small RNAs in Argonaut protein pulldown. Click here for file

Additional file 7 Confirmation of some of the predicted targets using two published degradome data in rice. Click here for file

Additional file 8 **Candidate miRNAs that are derived from transposons or repeats.** Candidate miRNAs that were predicted using anchor sRNAs that match annotated transposons or repeats, or have high copy number (>20) in the genome. Click here for file

Additional file 9 Detailed information of 80 candidate miRNAs that were derived from transposable elements or repeats and were validated with expression data from DCL1, DCL3, and RDR2 RNAi lines. Click here for file

Additional file 10 **MITEs are rich sources for generating miRNAs.** Relative abundance of different classes of TEs in the genome is compared to the proportion of miRNAs that were derived from different classes of TEs. MITEs are apparently enriched in TEs from which miRNAs were derived. Click here for file

Additional file 11 **List of oligos that were used as probes to detect the expression of miRNAs in Northern blot assays.** Probes are complimentary to the predicted mature miRNA sequences. Click here for file
