# Supplementary material for: High throughput sequencing reveals novel and abiotic stress-regulated microRNAs in the inflorescences of rice
Source: BMC Plant Biol. 2012 Aug 3;12:132. doi: 10.1186/1471-2229-12-132 (PMC3431262; doi:10.1186/1471-2229-12-132)
Supplement: Additional file 1 — Summary of small RNA sequencing data. Detailed information of preprocessing of small RNA reads from four libraries in rice inflorescences. [file 1471-2229-12-132-S1.doc]

**Additional File 1**. Summary of small RNA sequencing data

| Library | Raw reads | Clean reads | Unique reads | Perfect match to the genome |
| --- | --- | --- | --- | --- |
| Control | 10487626 | 5328145 | 3093872 | 4619308 |
| Drought | 7827240 | 4186380 | 2369547 | 3577137 |
| Cold | 6290902 | 3524691 | 2094479 | 3019815 |
| Salt | 6331226 | 3992166 | 2260975 | 3481663 |
