# Supplementary material for: High throughput sequencing reveals novel and abiotic stress-regulated microRNAs in the inflorescences of rice
Source: BMC Plant Biol. 2012 Aug 3;12:132. doi: 10.1186/1471-2229-12-132 (PMC3431262; doi:10.1186/1471-2229-12-132)
Supplement: Additional file 3 — Predicted secondary structures of the precursors of new miRNA candidates. Free energy and miRNA ID are on the bottom of each structure. Nucleotides that constitute mature RNAs are drawn in blue. [file 1471-2229-12-132-S3.pdf]

Output of sir_graph (©)
mfold_util 4.6

Created Thu Jun 14 20:12:07 2012

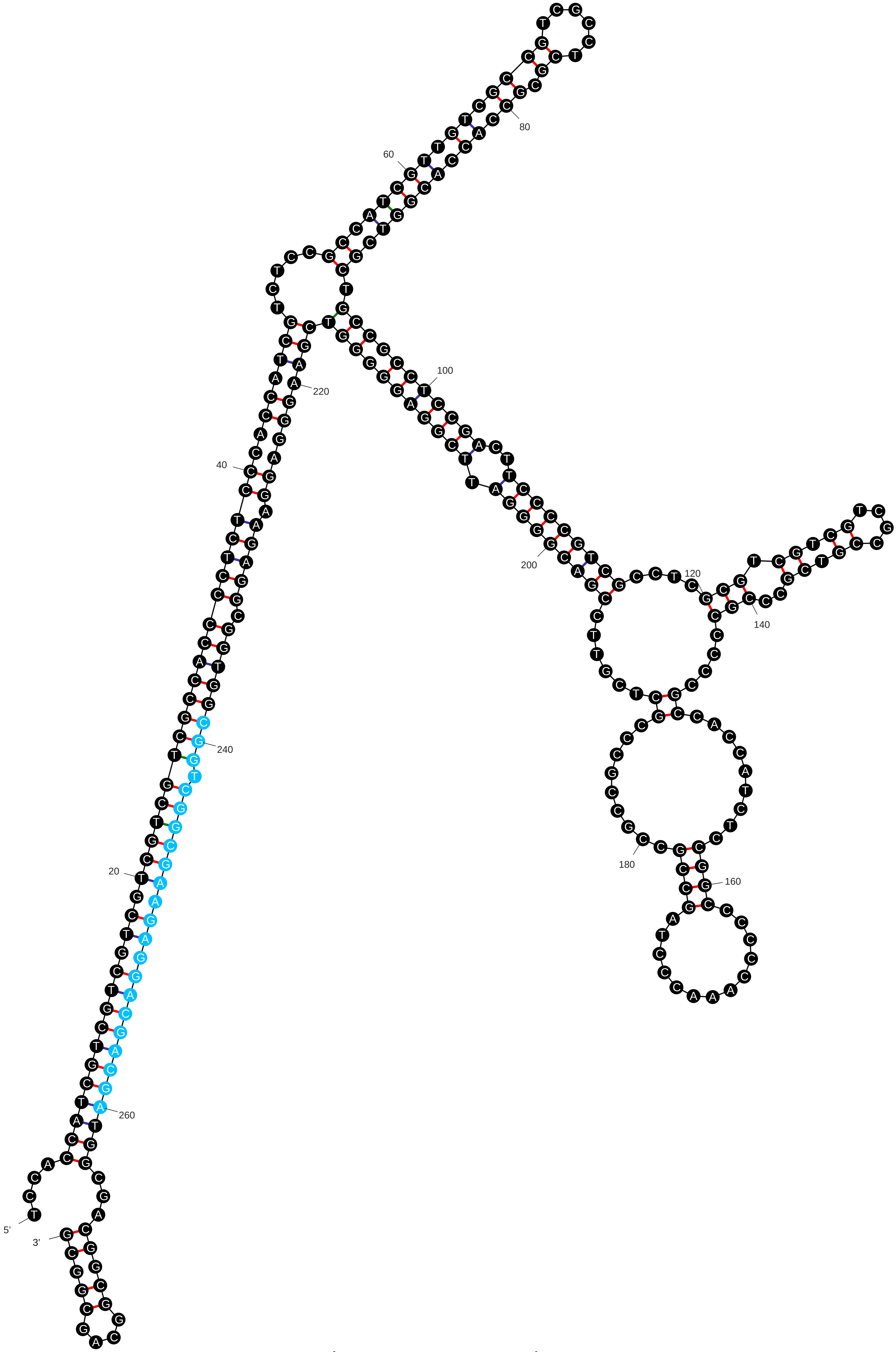

dG = -133.70 osa-cand001

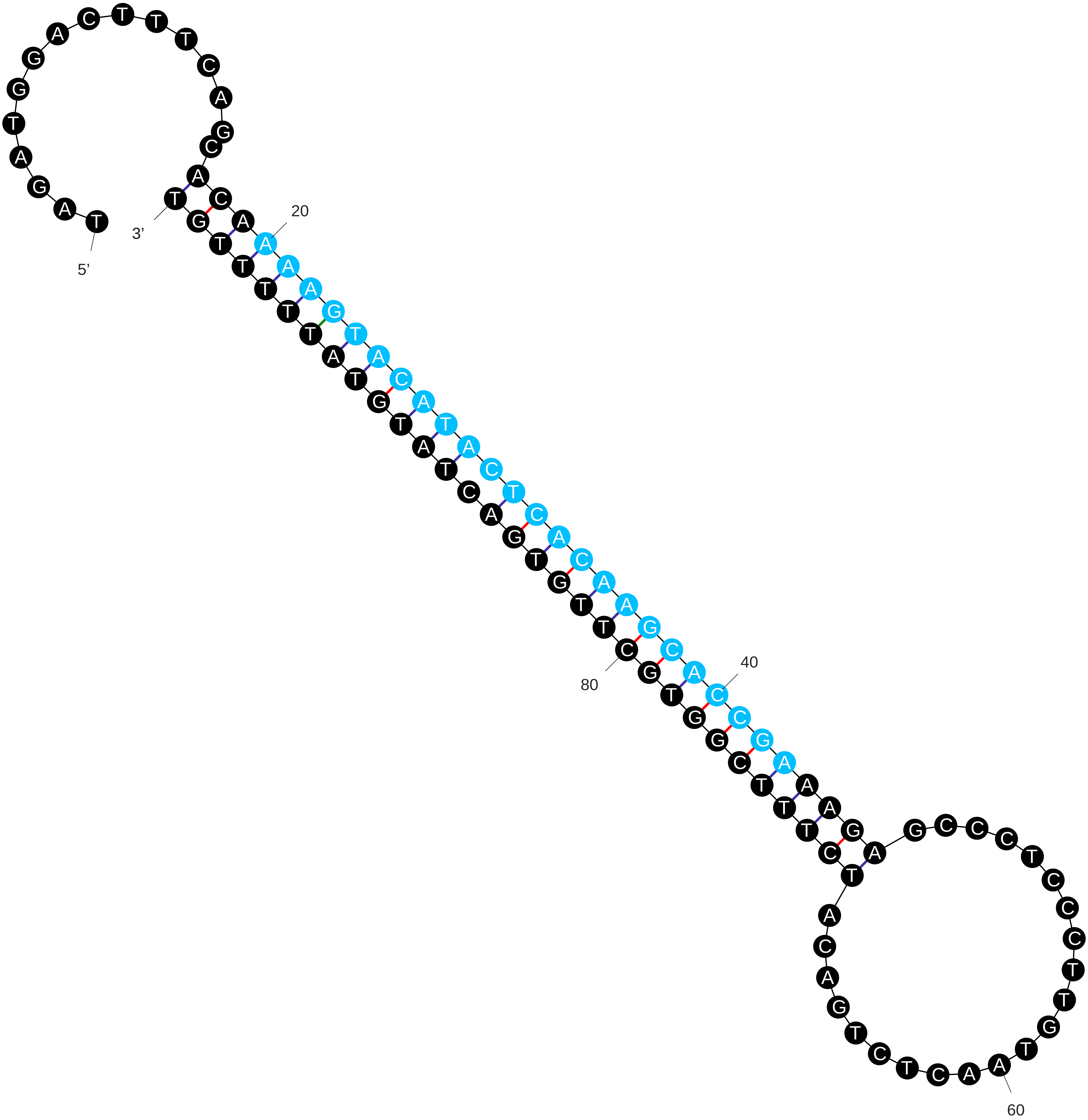

dG = -42.80 osa-cand002

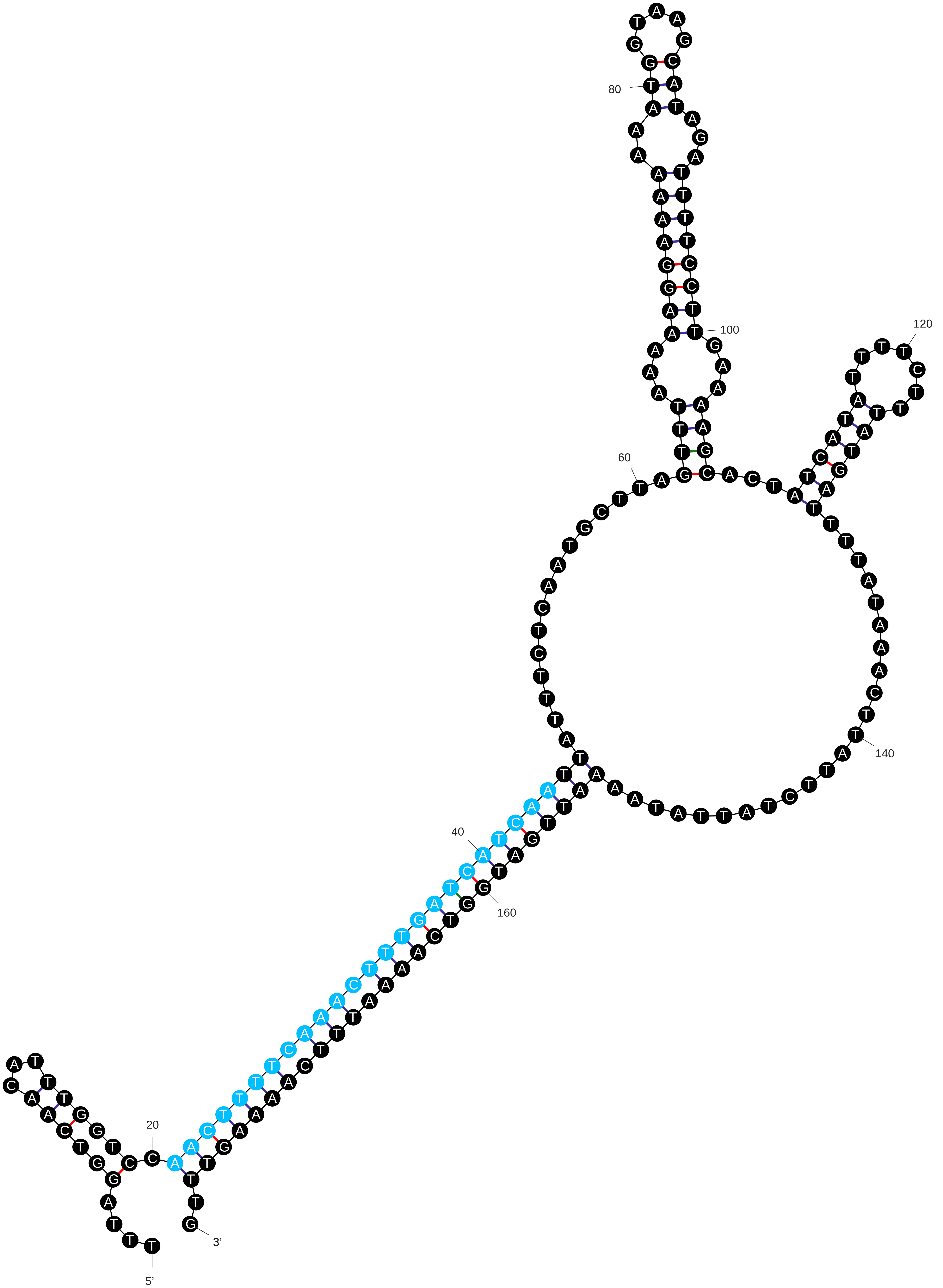

dG = -36.80 osa-cand003

Output of sir_graph (©)
mfold_util 4.6

Created Thu Jun 14 20:17:07 2012

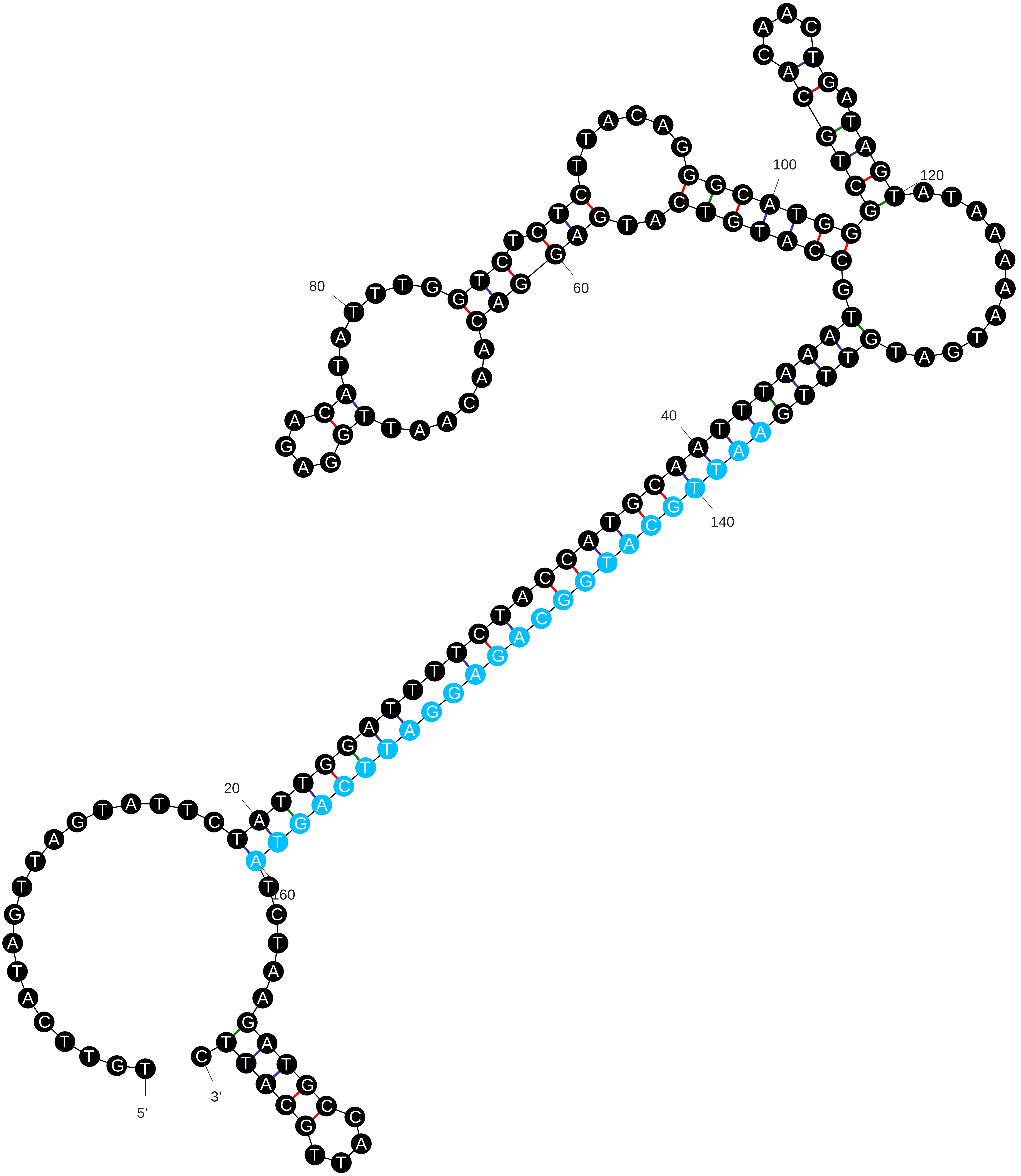

dG = -56.10 osa-cand004

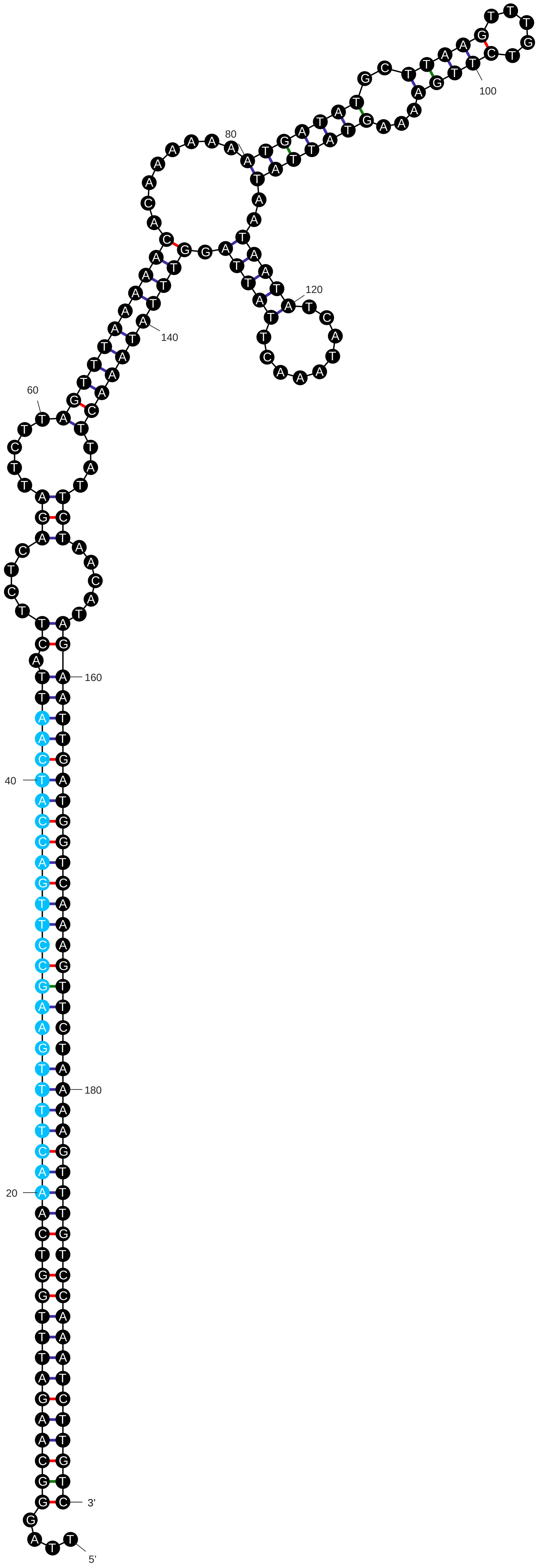

dG = -65.10 osa-cand005a

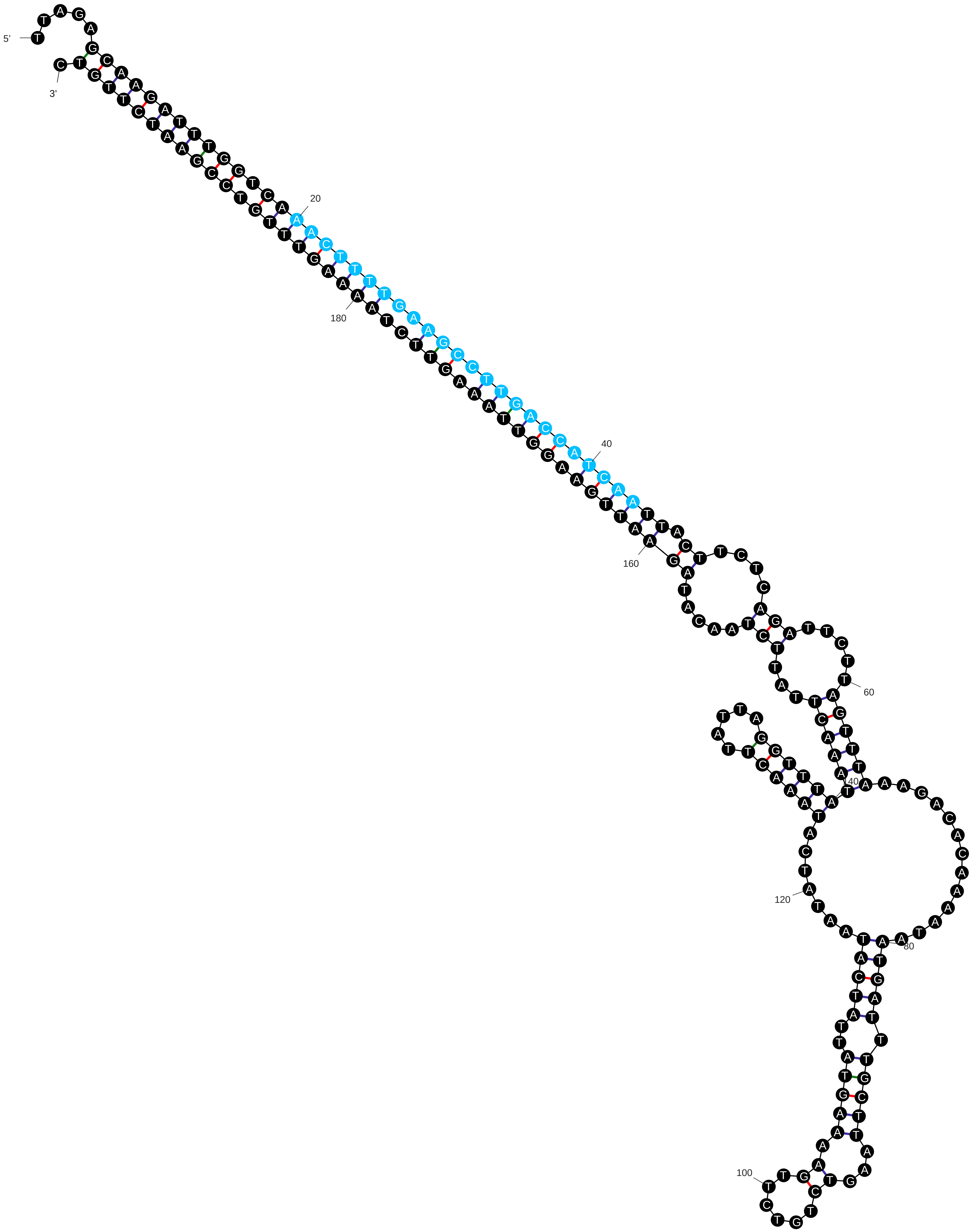

dG = -55.50 osa-cand005b

Output of sir_graph (©)
mfold_util 4.6

Created Thu Jun 14 20:21:01 2012

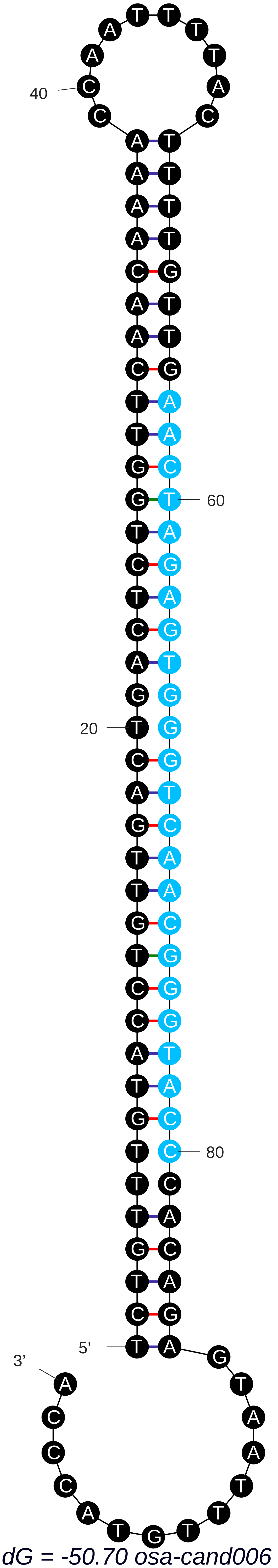

dG = -50.70 osa-cand006

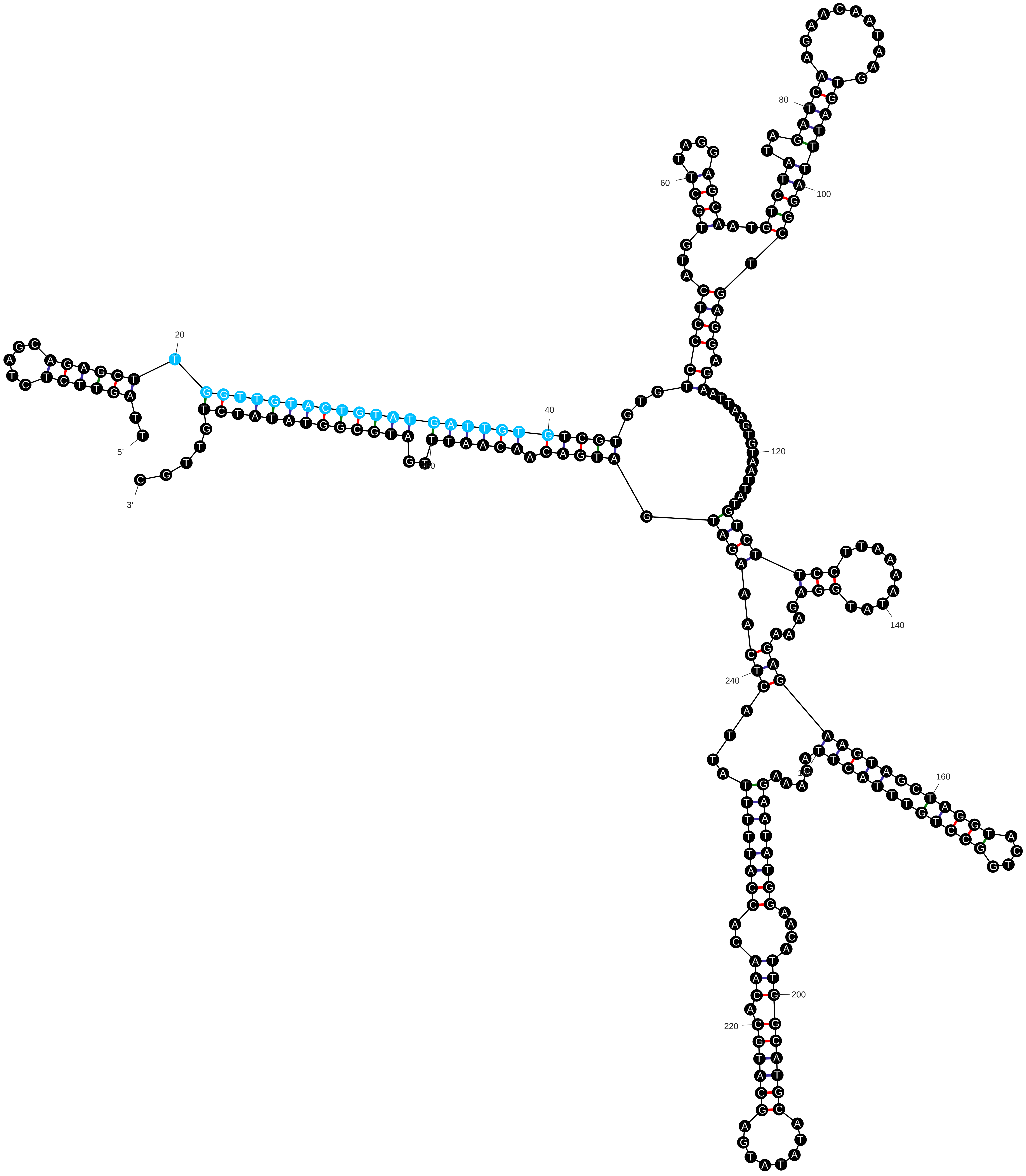

dG = -68.00 osa-cand007

Output of sir_graph (©)
mfold_util 4.6

Created Thu Jun 14 20:24:27 2012

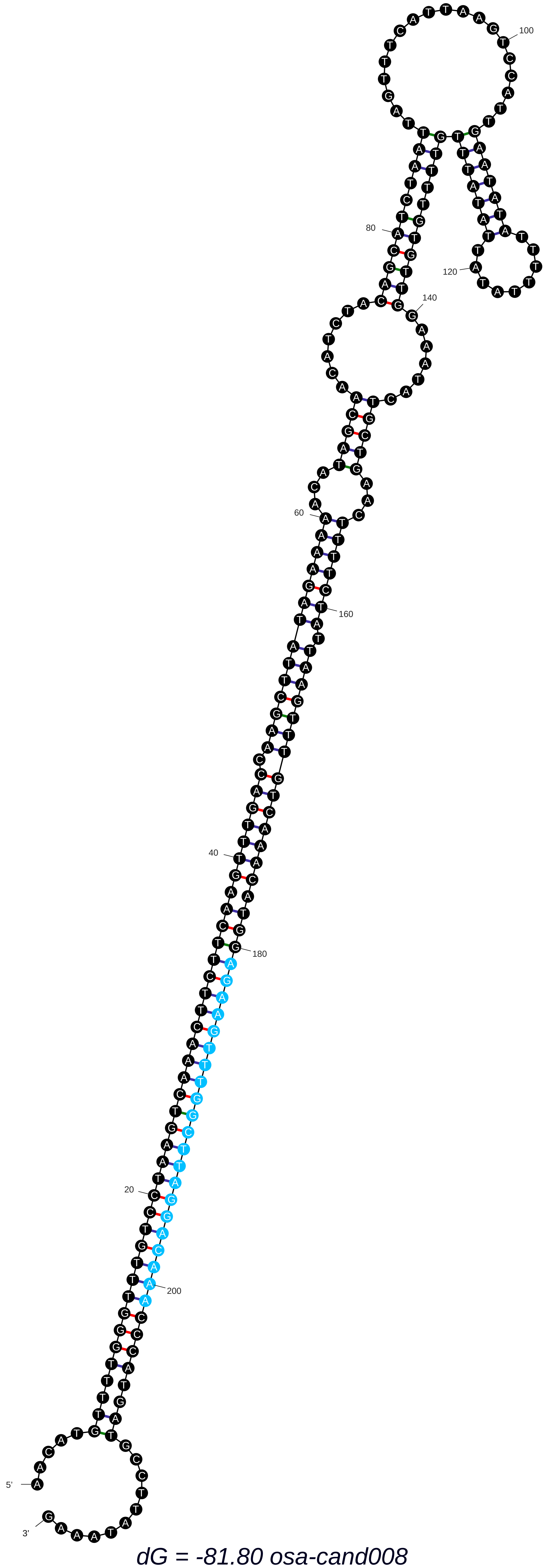

dG = -81.80 osa-cand008

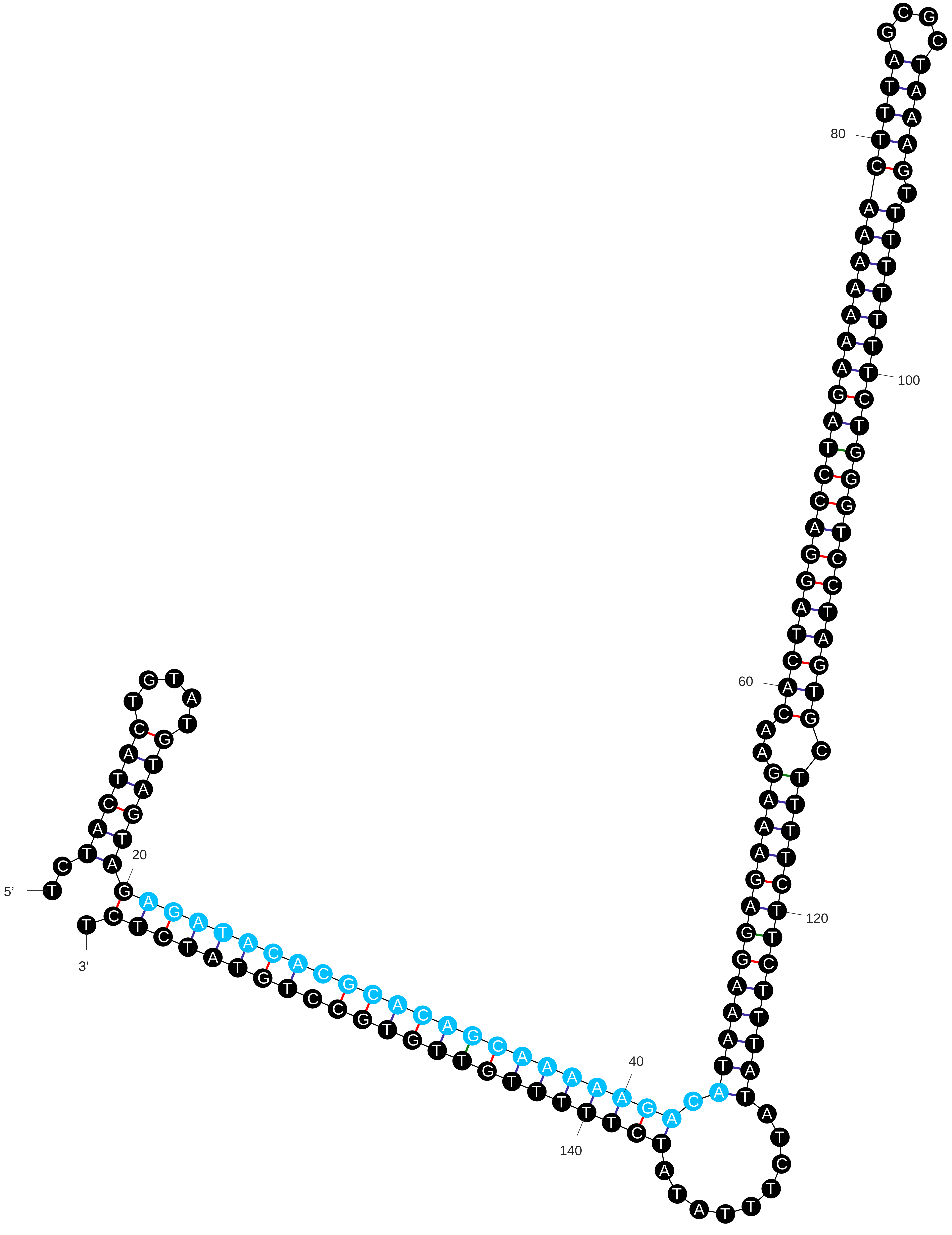

dG = -81.10 osa-cand009

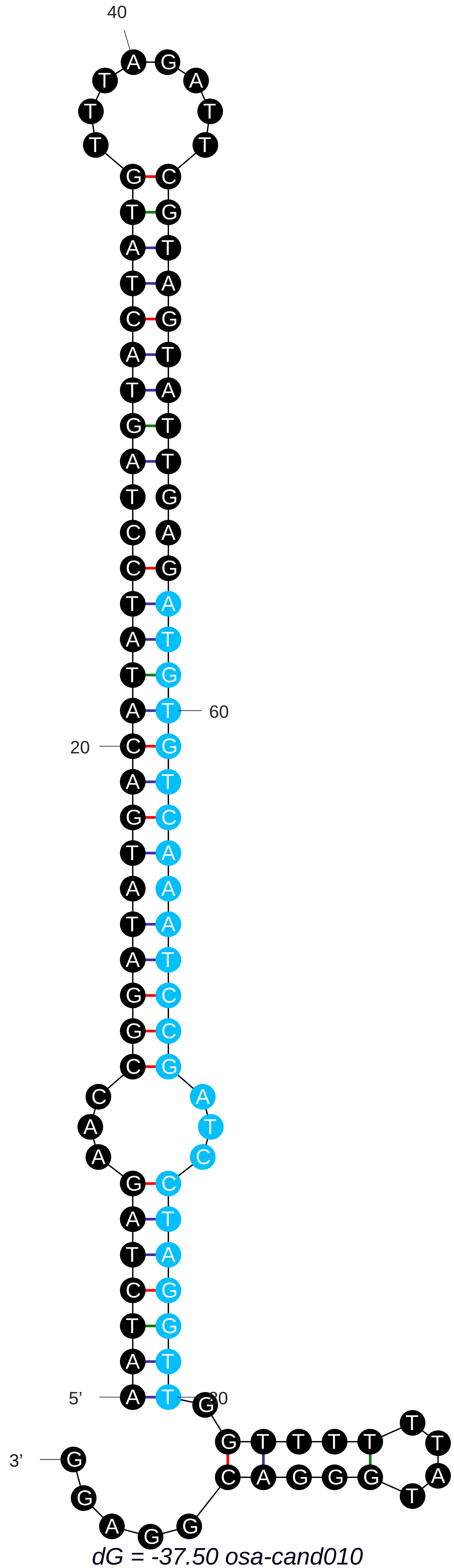

dG = -37.50 osa-cand010

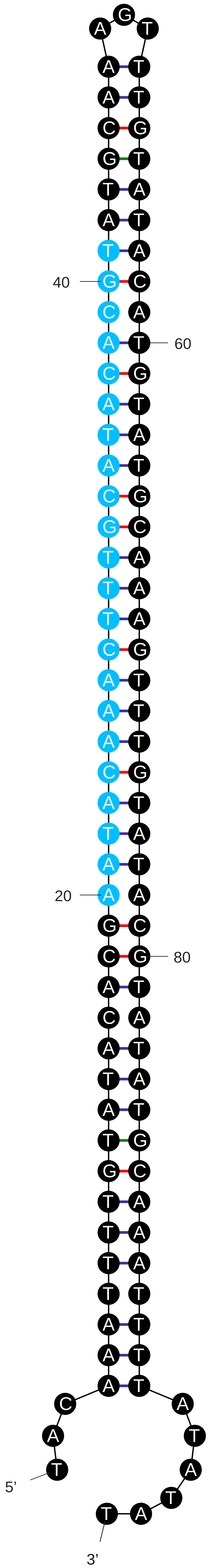

dG = -45.20 osa-cand011

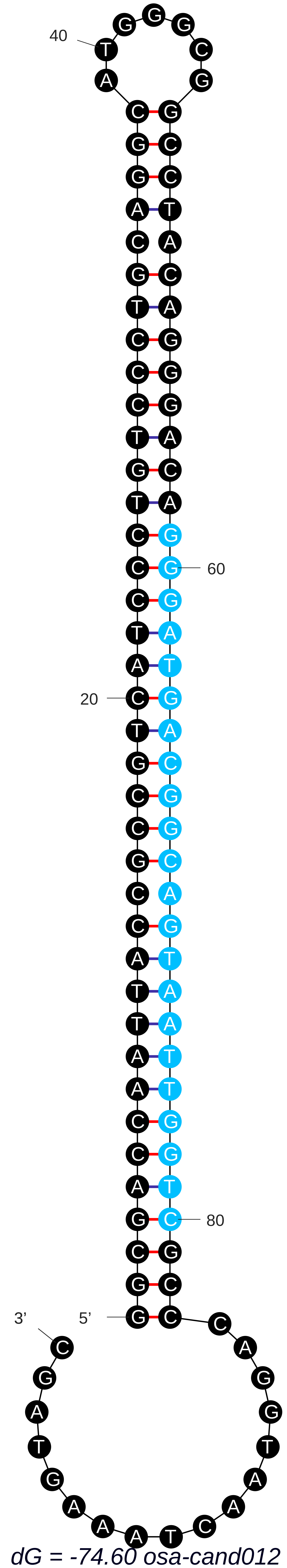

dG = -74.60 osa-cand012

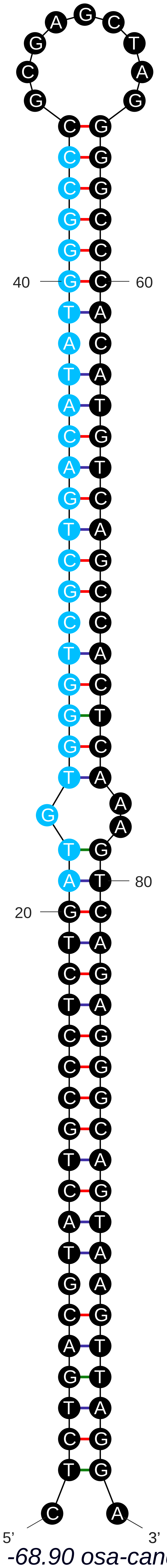

dG = -68.90 osa-cand013

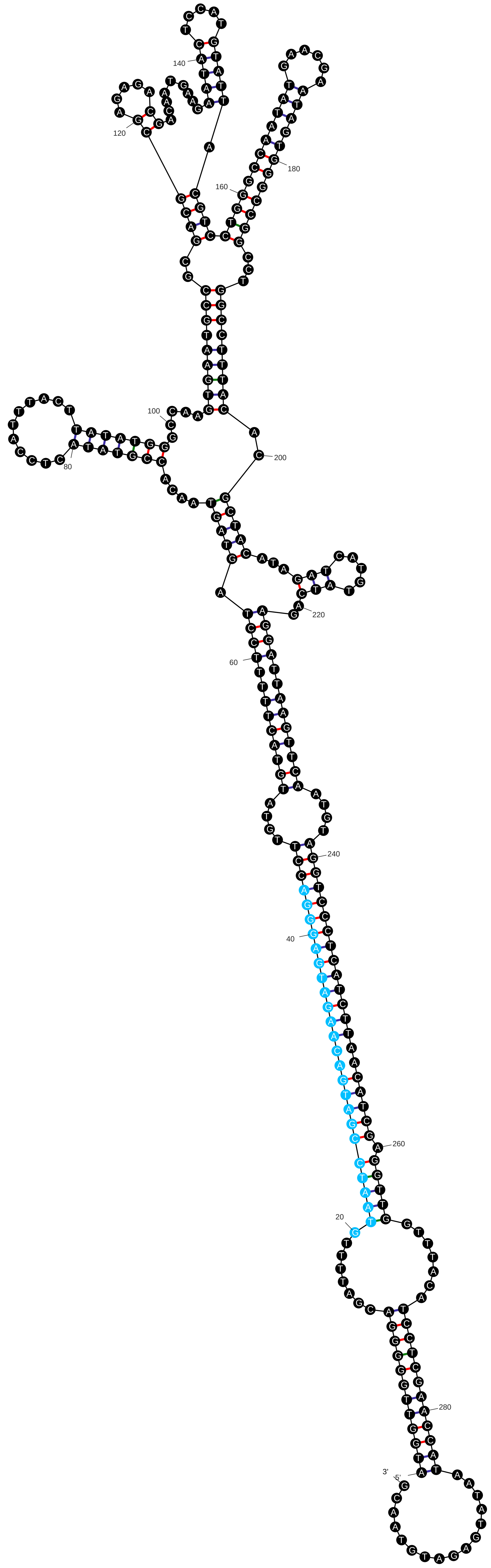

dG = -96.80 osa-cand014

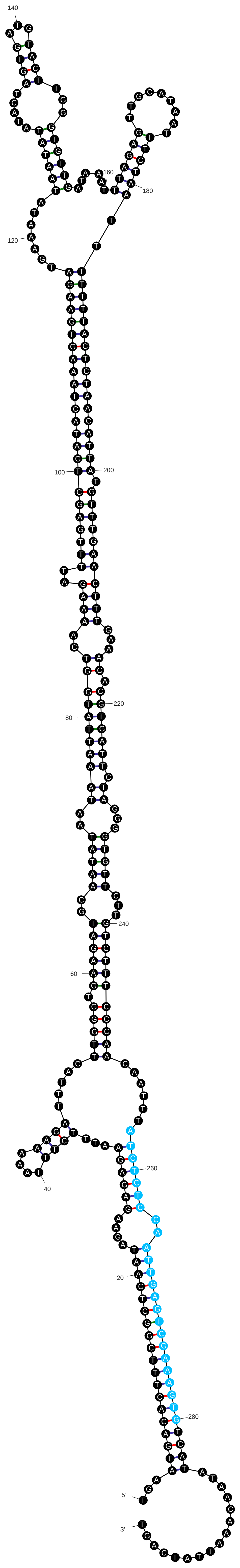

dG = -73.20 osa-cand015a

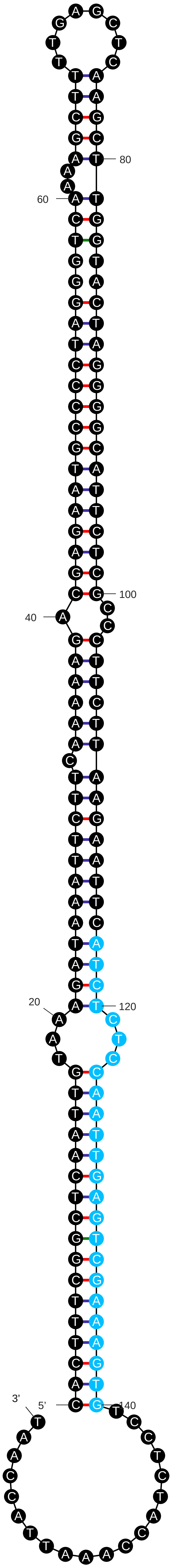

dG = -72.20 osa-cand015b

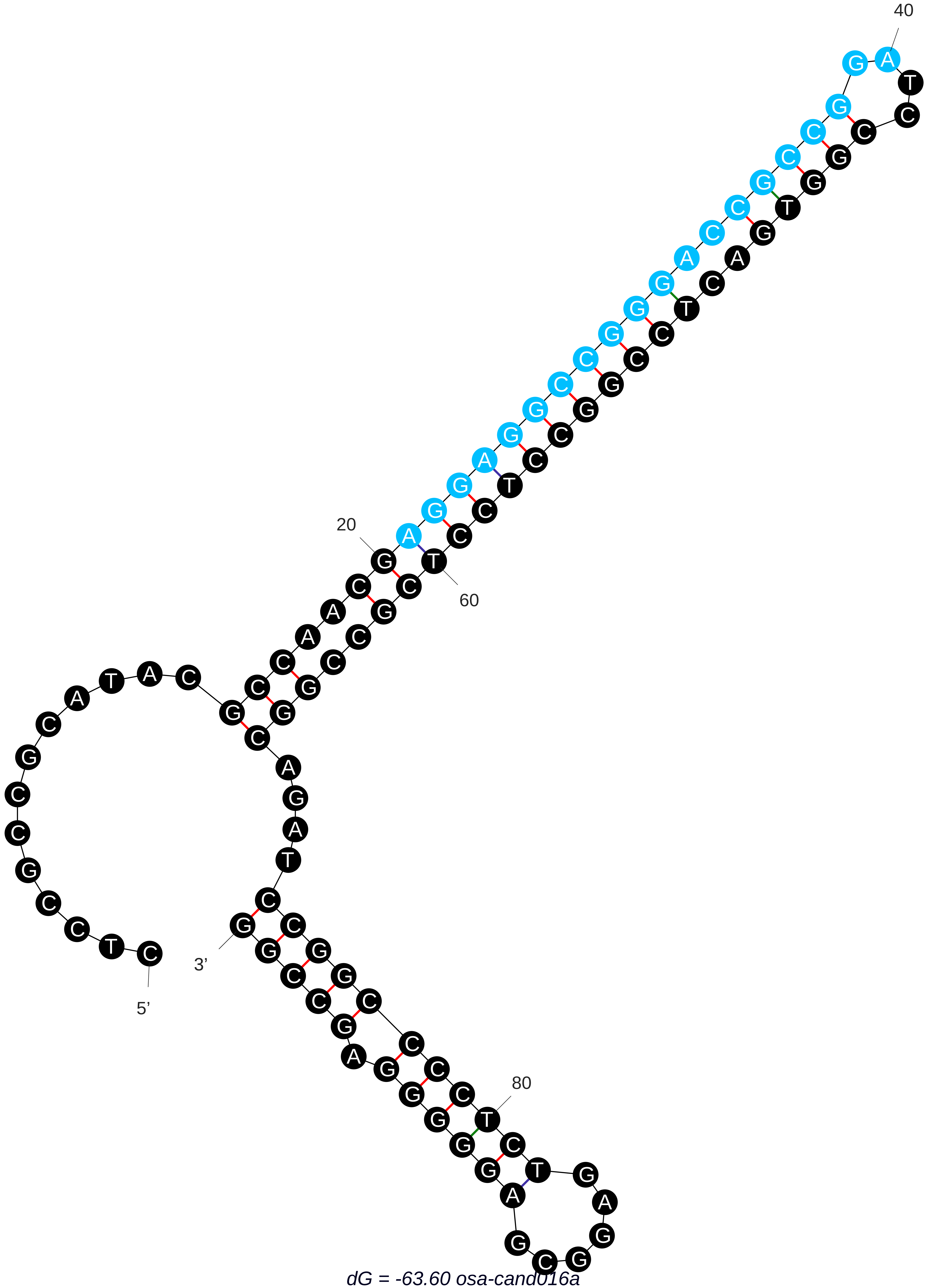

dG = -63.60 osa-cand016a

Output of sir_graph (©)
mfold_util 4.6

Created Thu Jun 14 20:39:30 2012

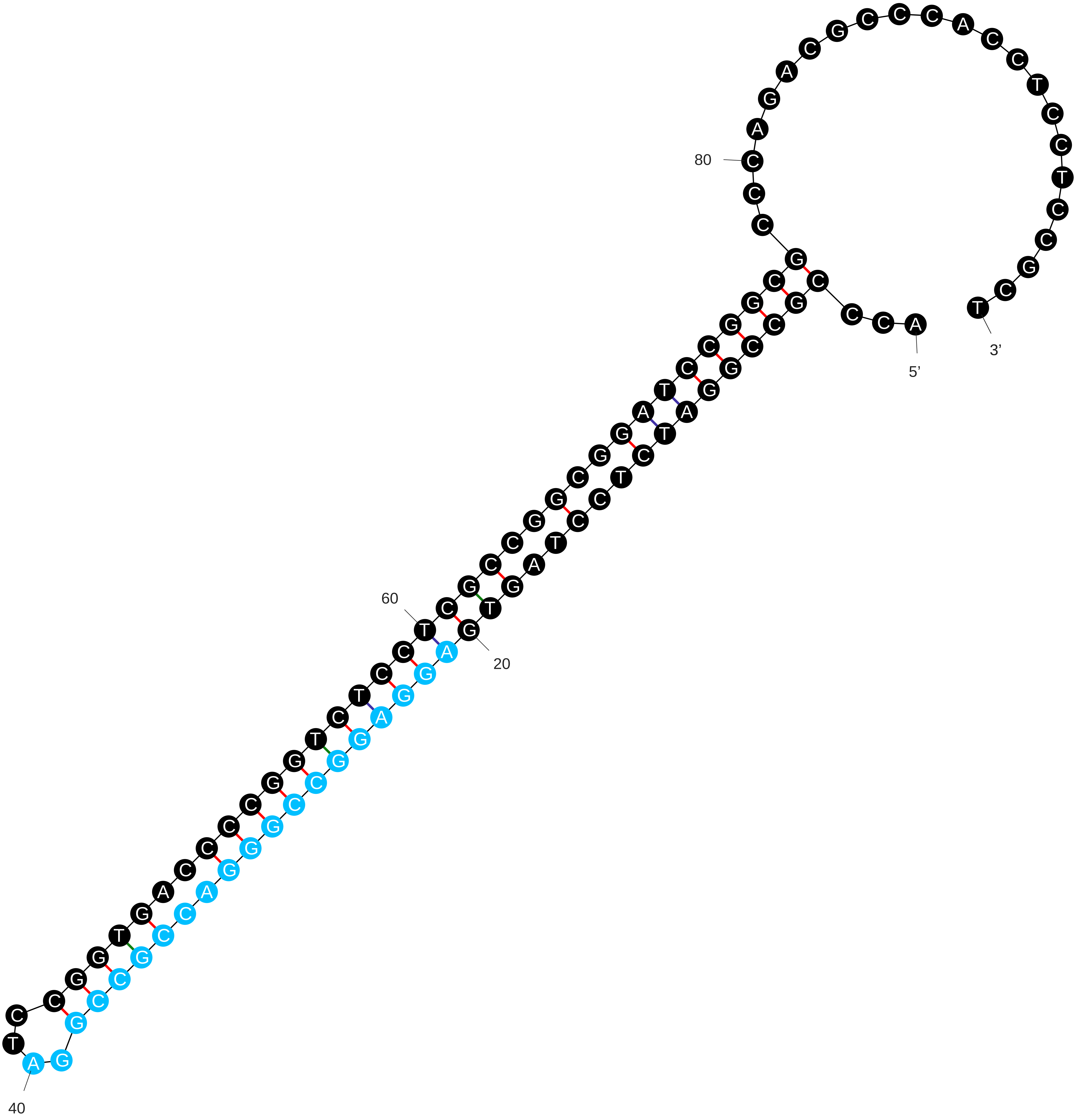

dG = -62.00 osa-cand016b

Output of sir_graph (©)
mfold_util 4.6

Created Thu Jun 14 20:44:24 2012

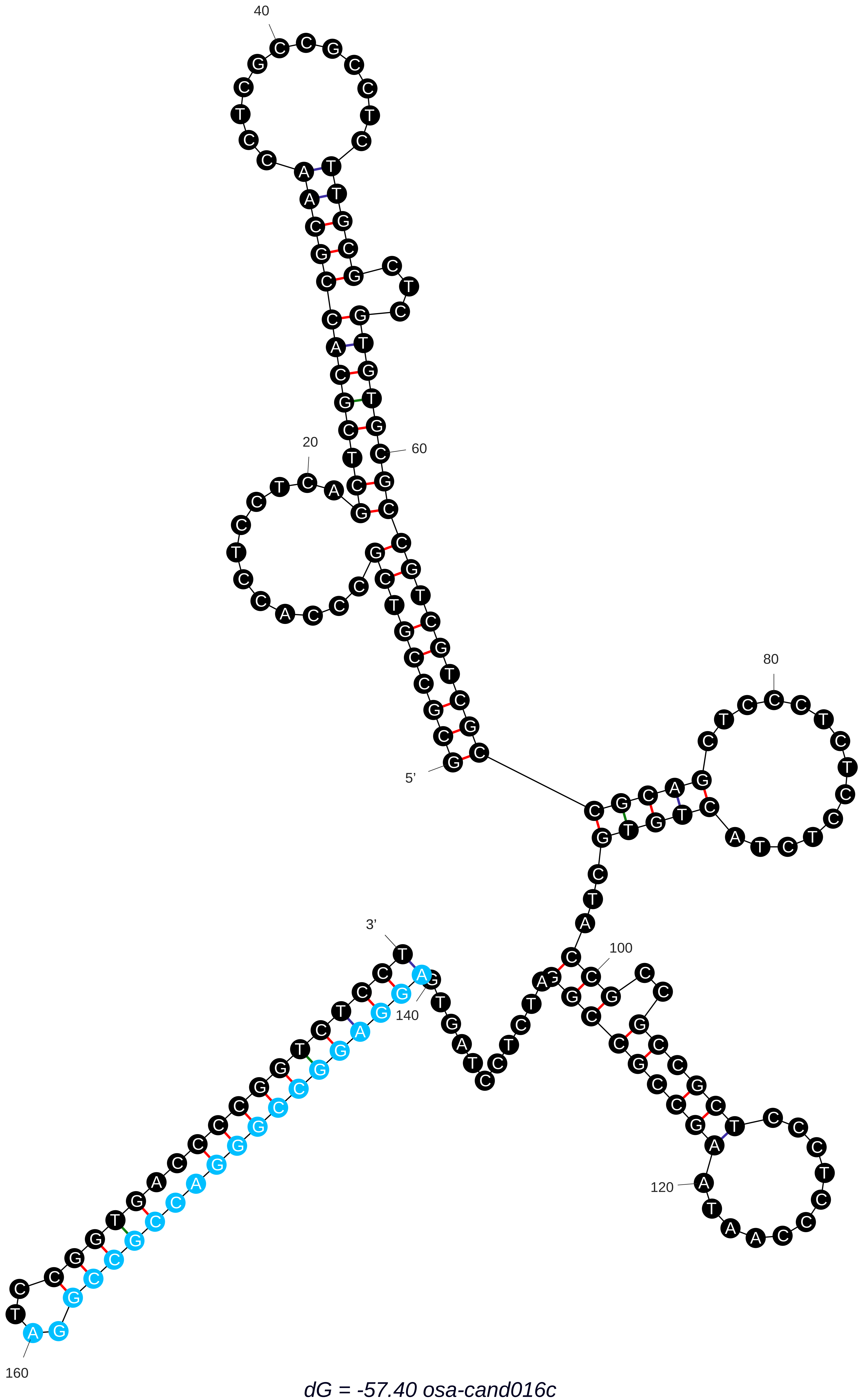

dG = -57.40 osa-cand016c

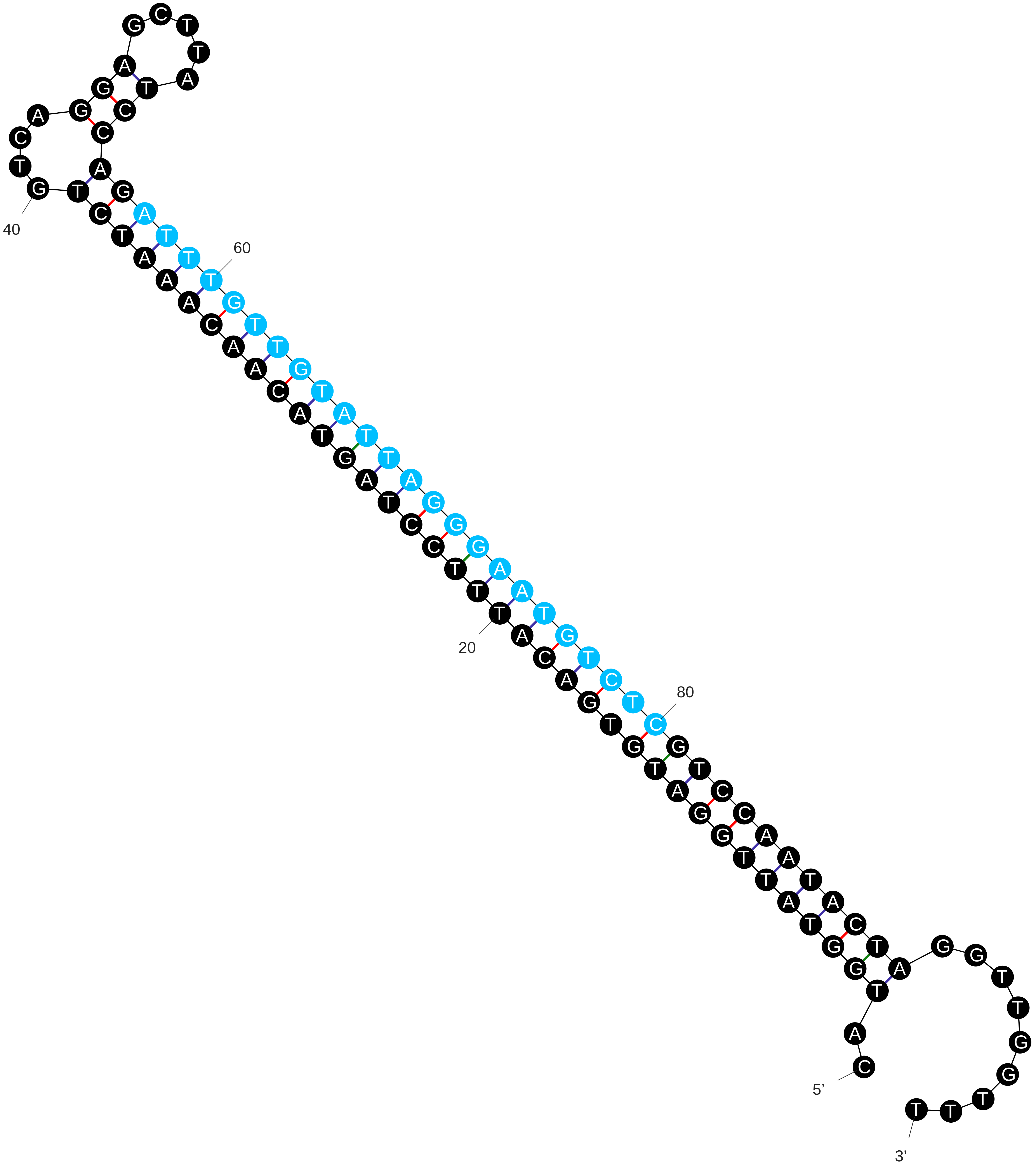

dG = -55.30 osa-cand017

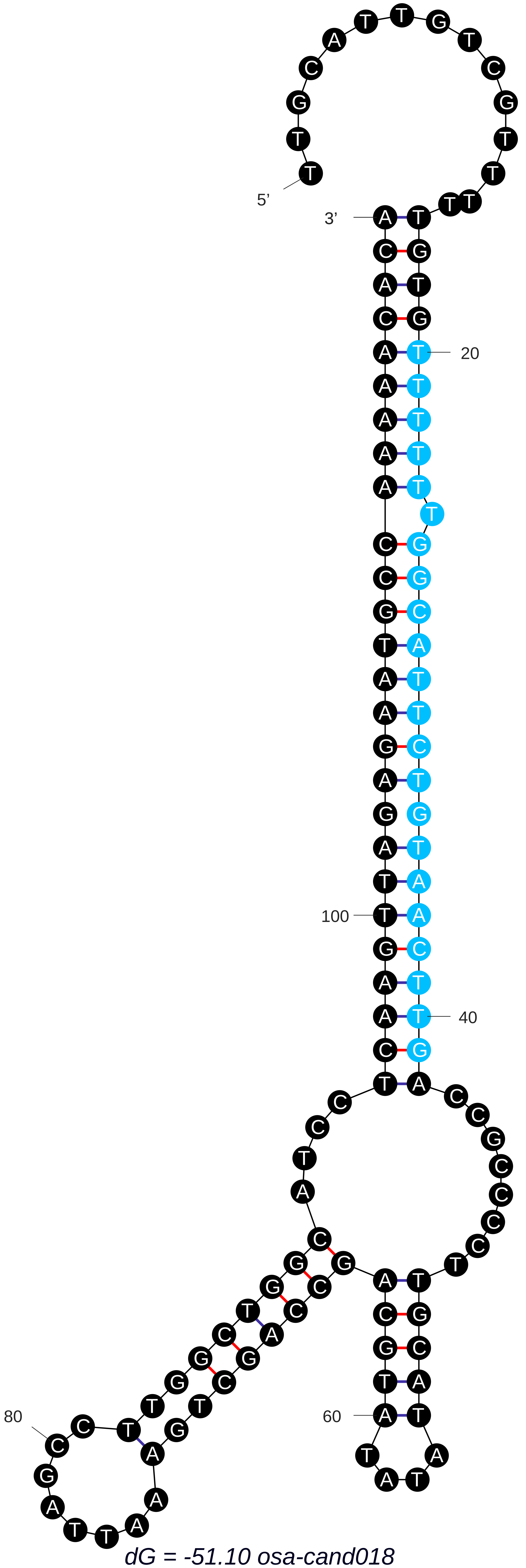

dG = -51.10 osa-cand018

Output of sir_graph (©)
mfold_util 4.6

Created Thu Jun 14 20:48:31 2012

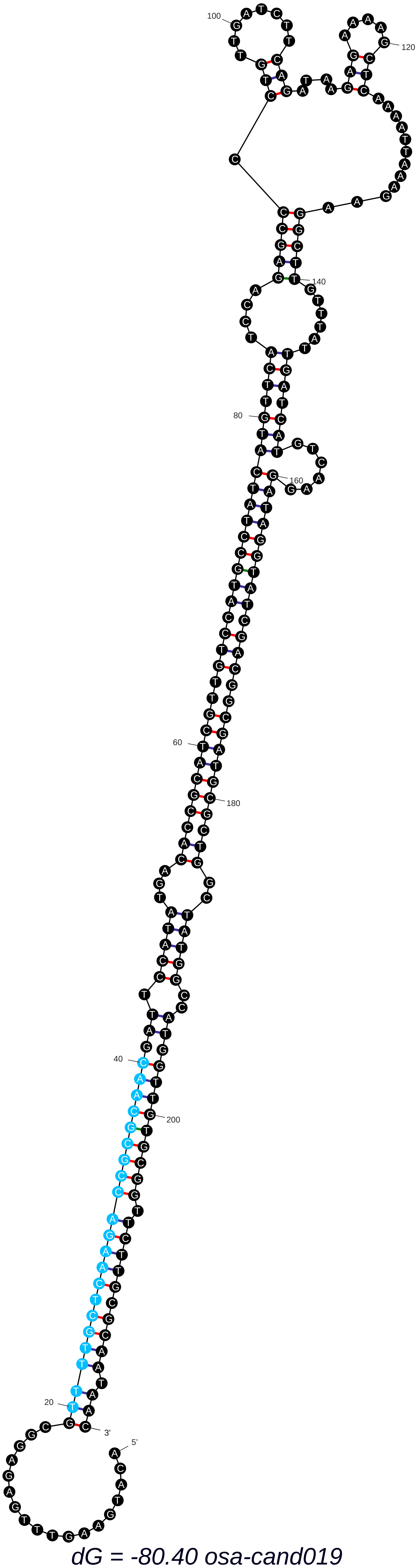

dG = -80.40 osa-cand019

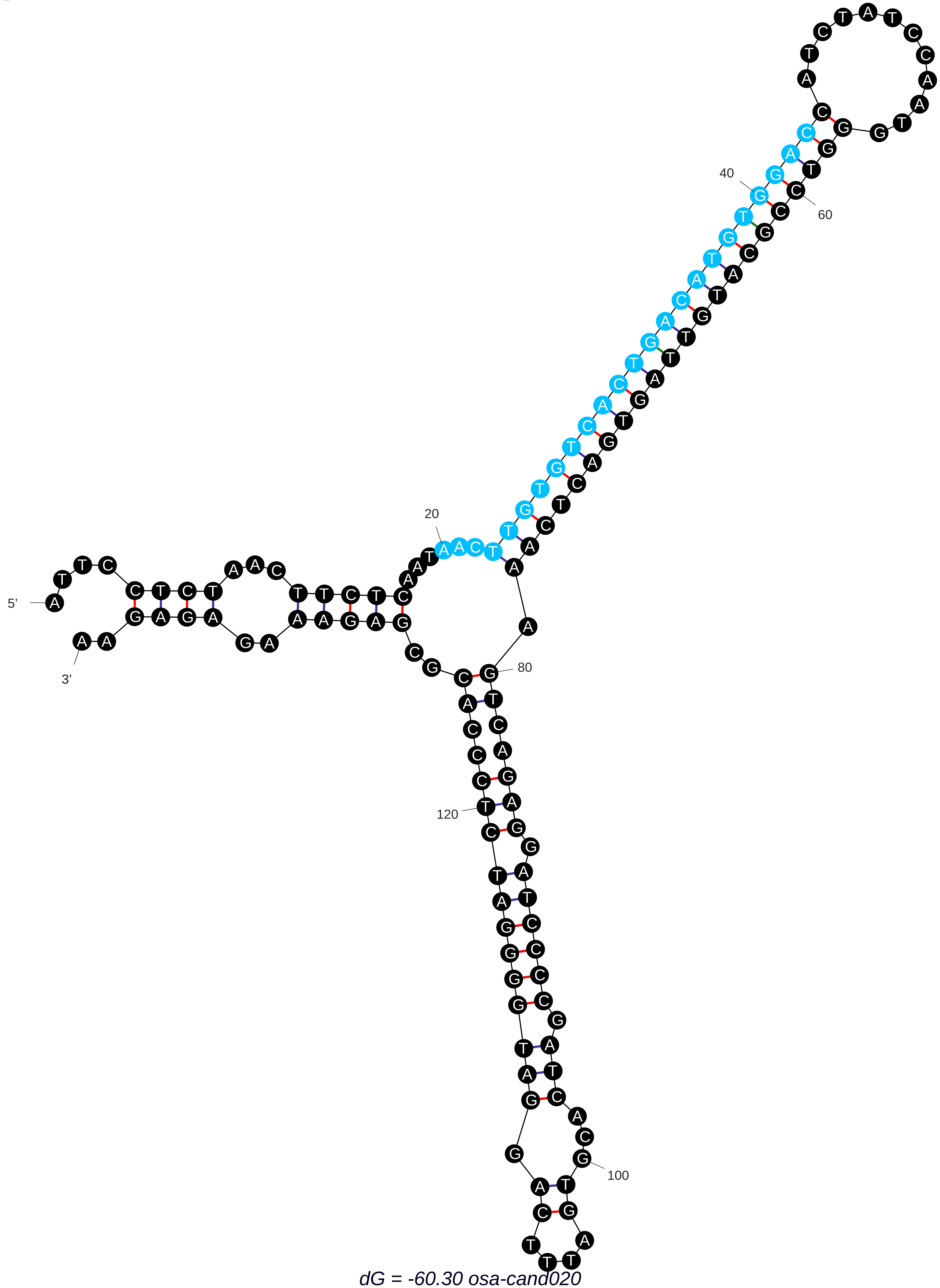

dG = -60.30 osa-cand020

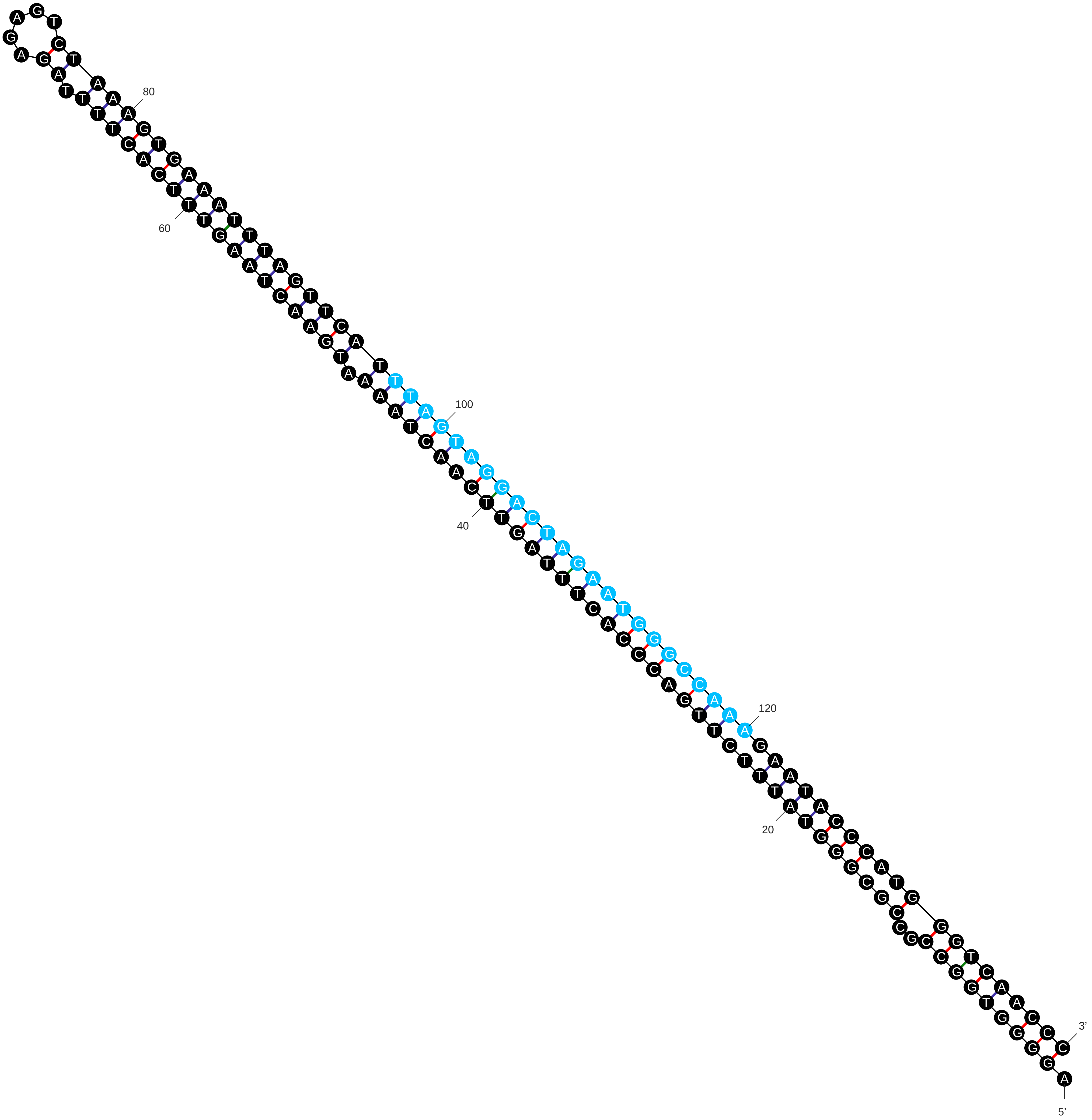

dG = -70.10 osa-cand021

Output of sir_graph (©)
mfold_util 4.6

Created Thu Jun 14 23:48:20 2012

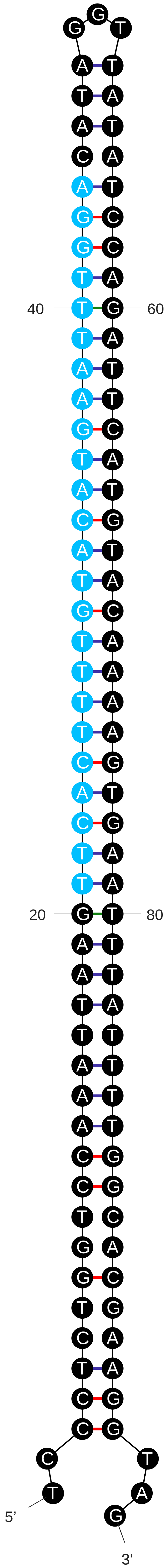

dG = -50.00 osa-cand022

Output of sir_graph (©)
mfold_util 4.6

Created Thu Jun 14 23:53:01 2012

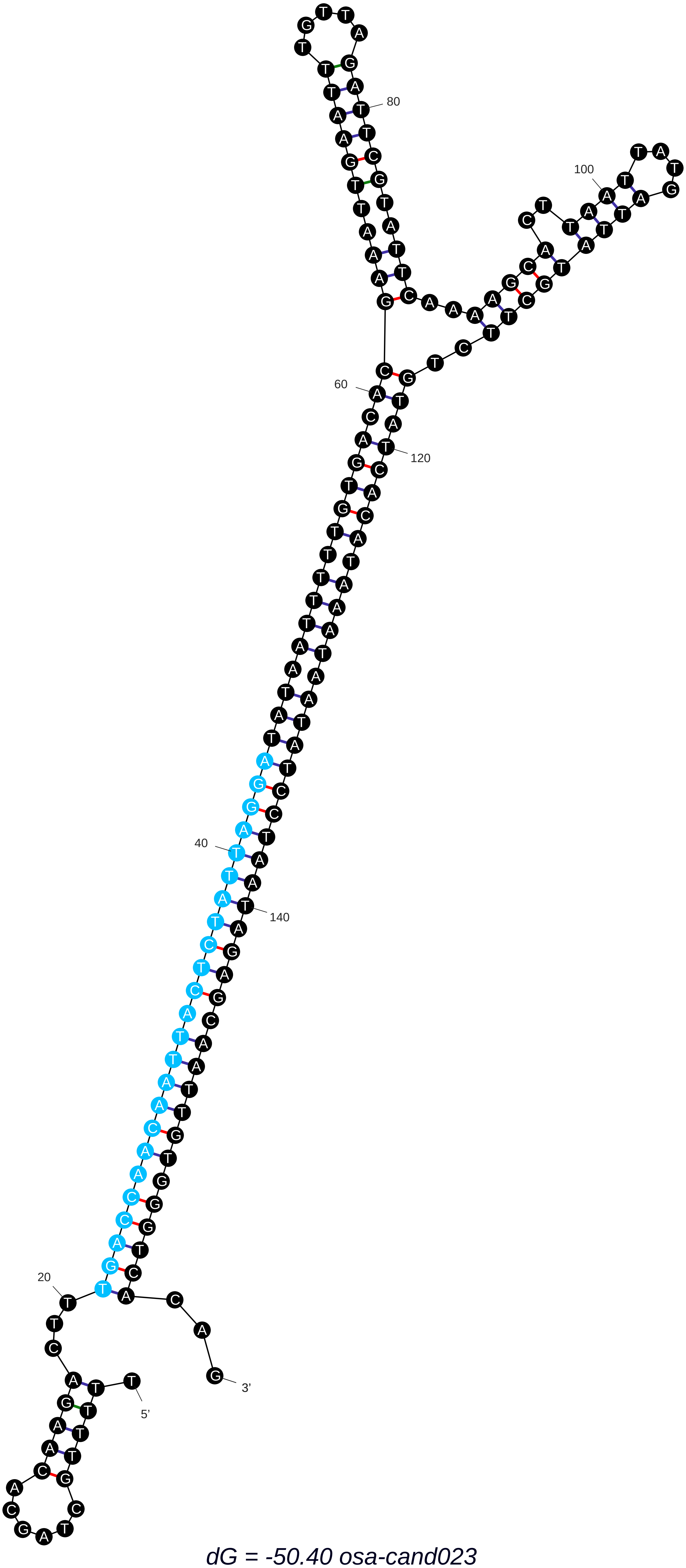

dG = -50.40 osa-cand023

Output of sir_graph (©)
mfold_util 4.6

Created Thu Jun 14 23:54:33 2012

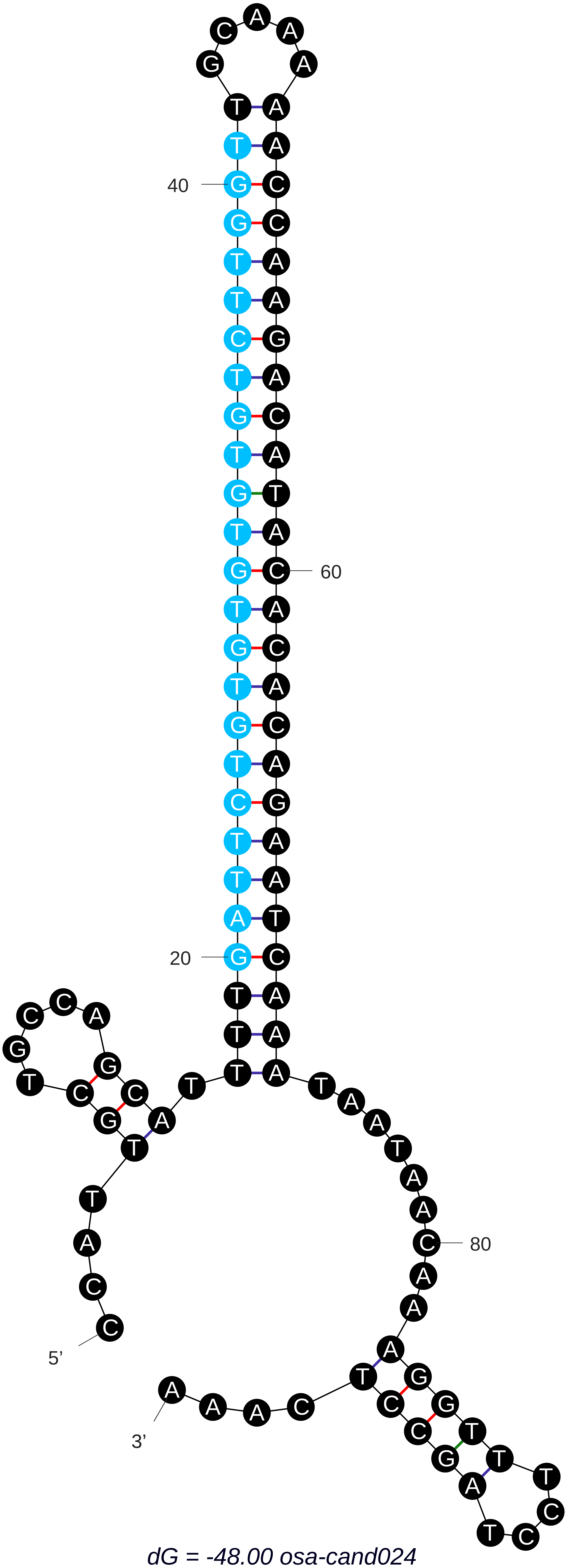

dG = -48.00 osa-cand024

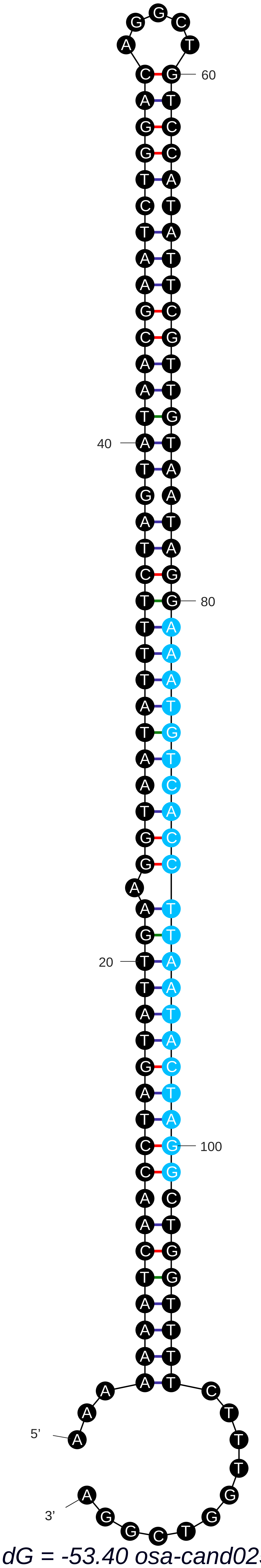

dG = -53.40 osa-cand025

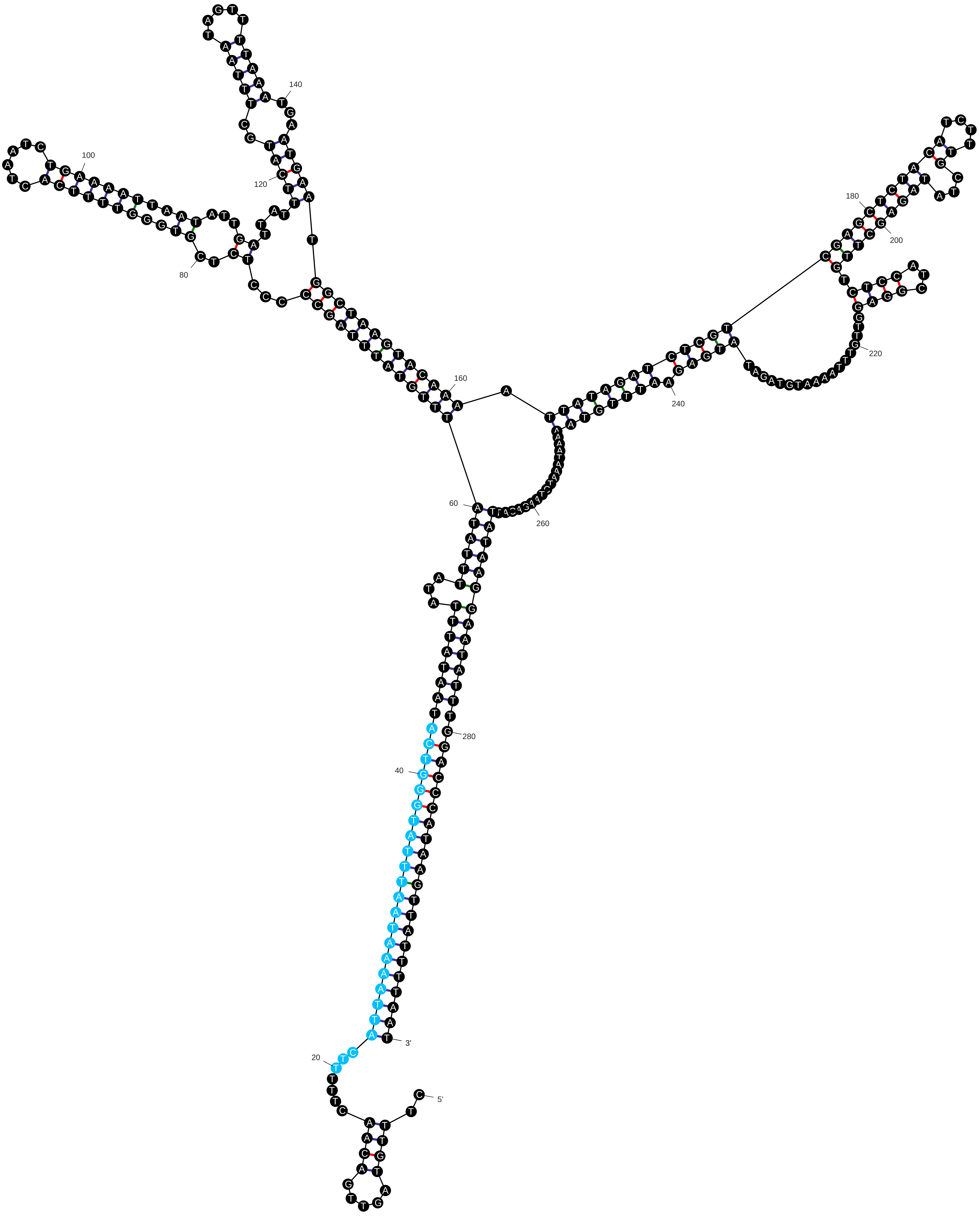

dG = -76.80 osa-cand026

Output of sir_graph (©)
mfold_util 4.6

Created Fri Jun 15 01:03:15 2012

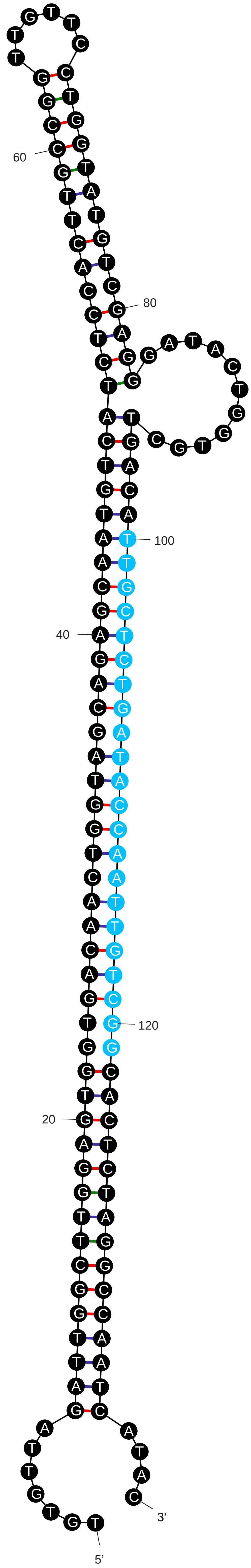

dG = -73.20 osa-cand027

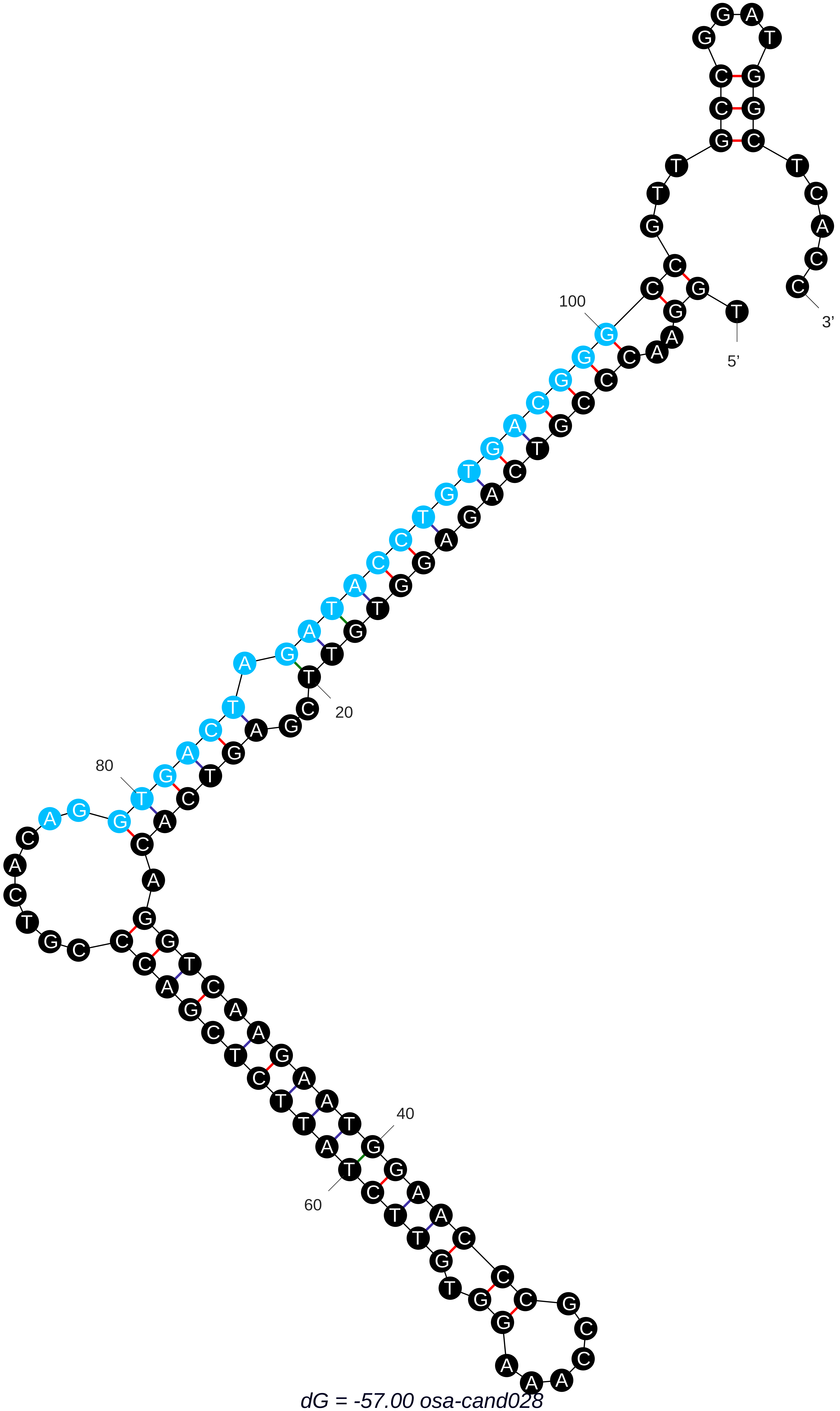

dG = -57.00 osa-cand028

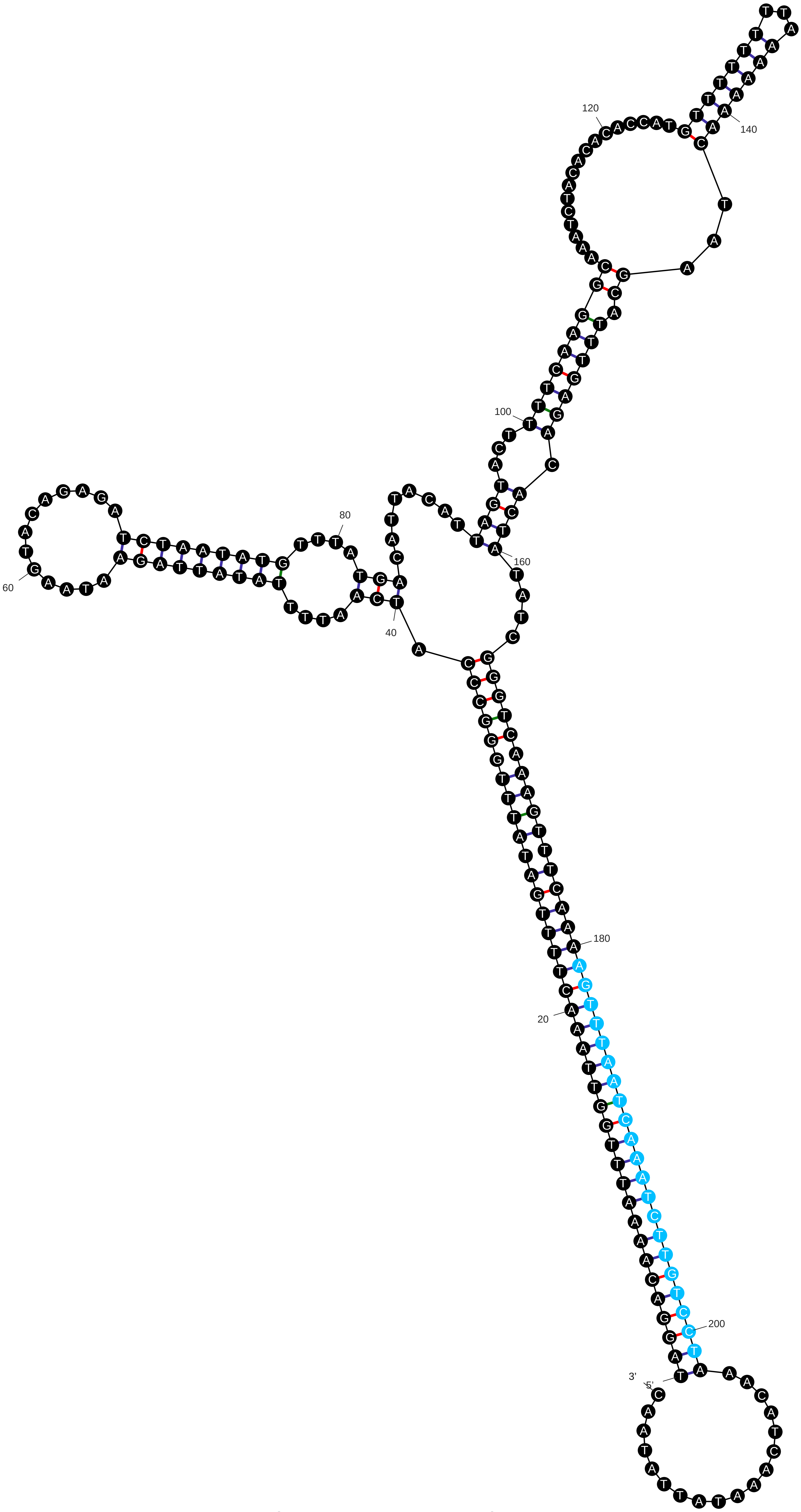

dG = -56.80 osa-cand029

Output of sir_graph (©)
mfold_util 4.6

Created Fri Jun 15 01:12:37 2012

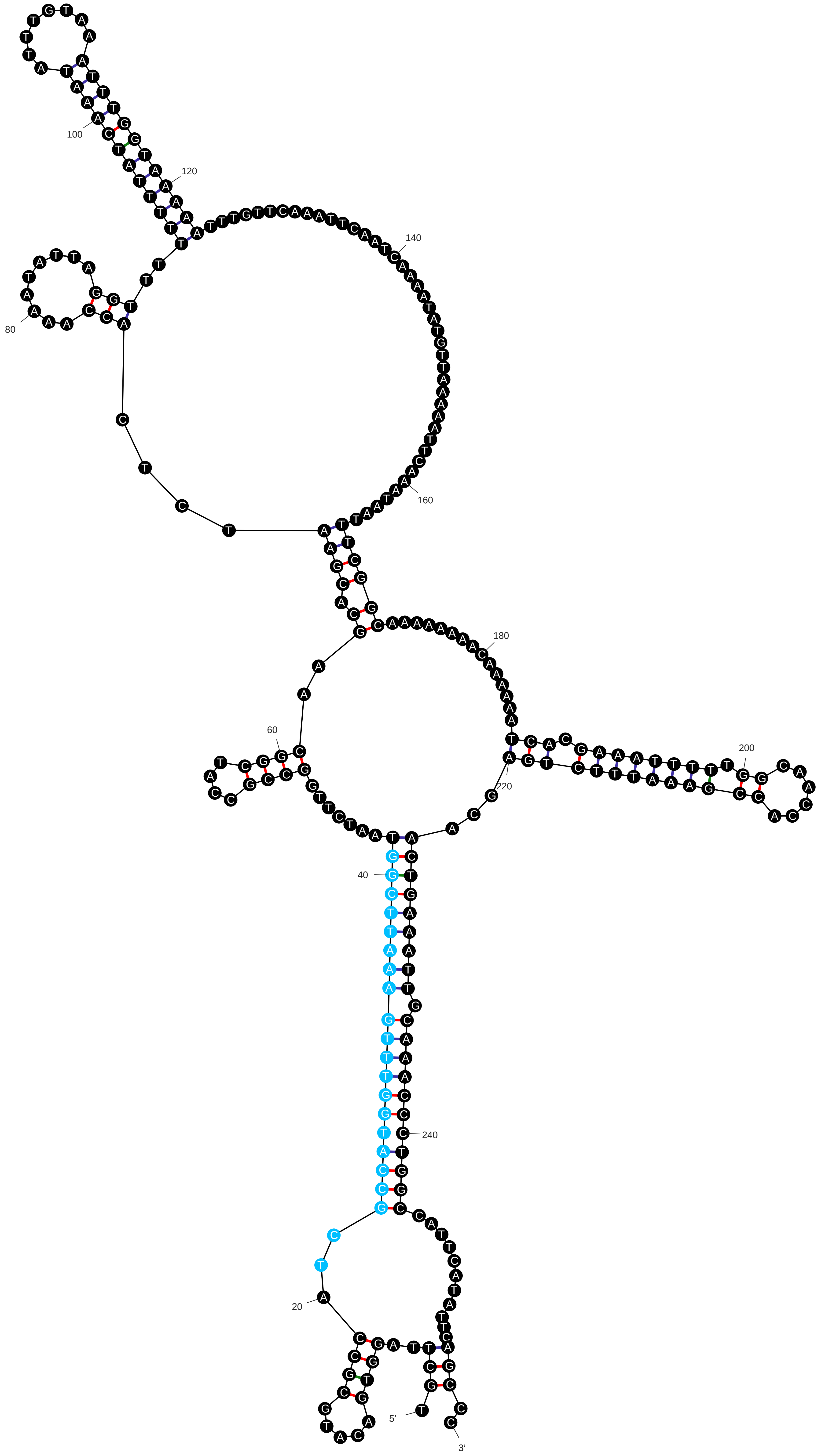

dG = -55.40 osa-cand030

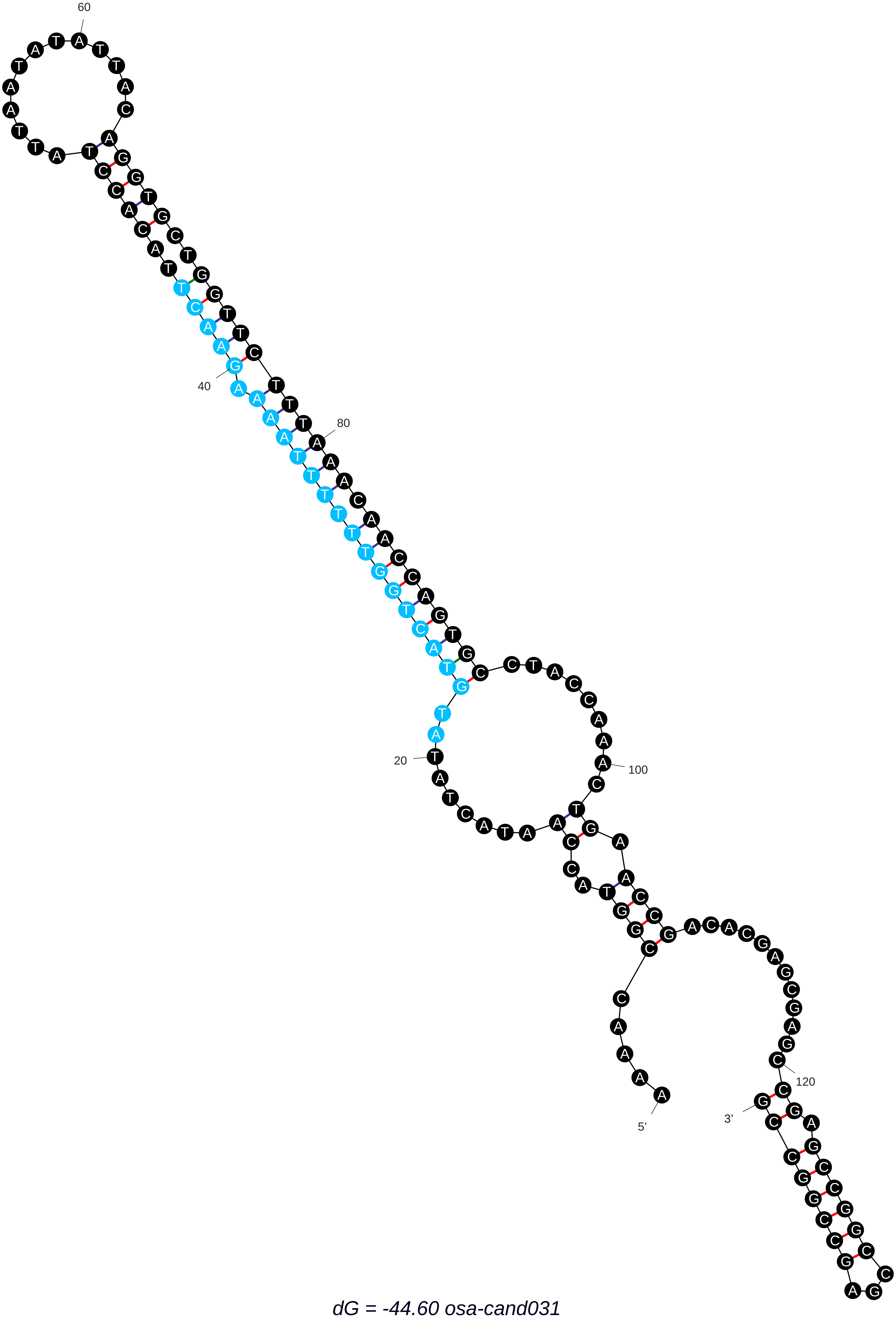

dG = -44.60 osa-cand031

Output of sir_graph (©)
mfold_util 4.6

Created Fri Jun 15 01:26:36 2012

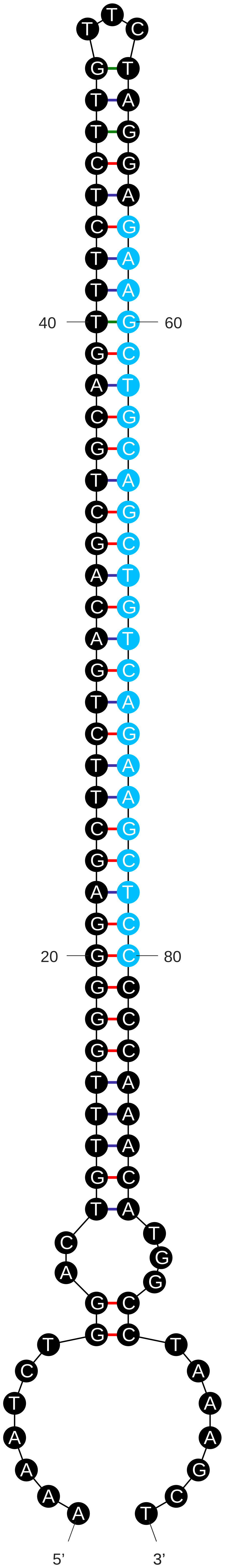

dG = -73.90 osa-cand032

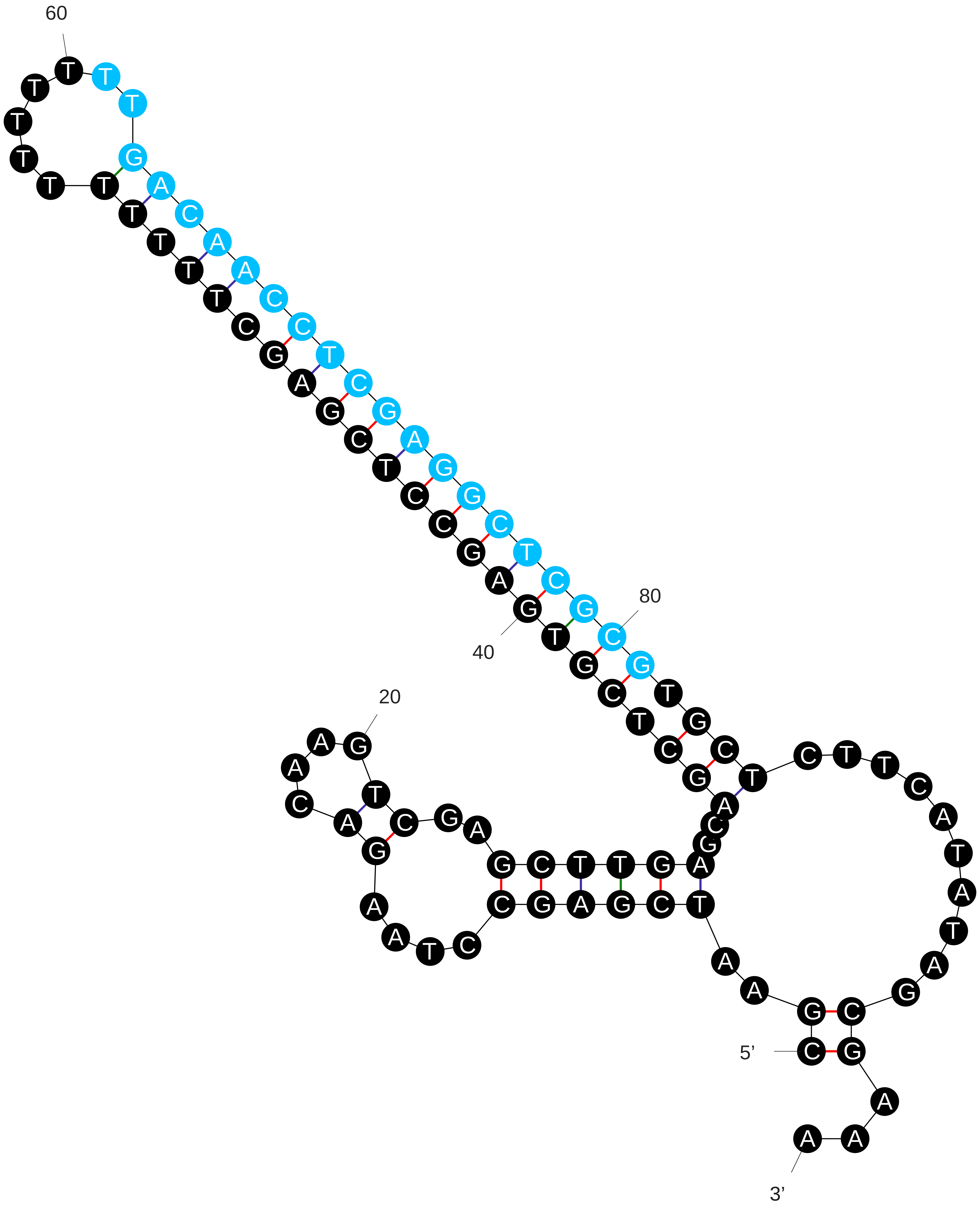

dG = -36.60 osa-cand033

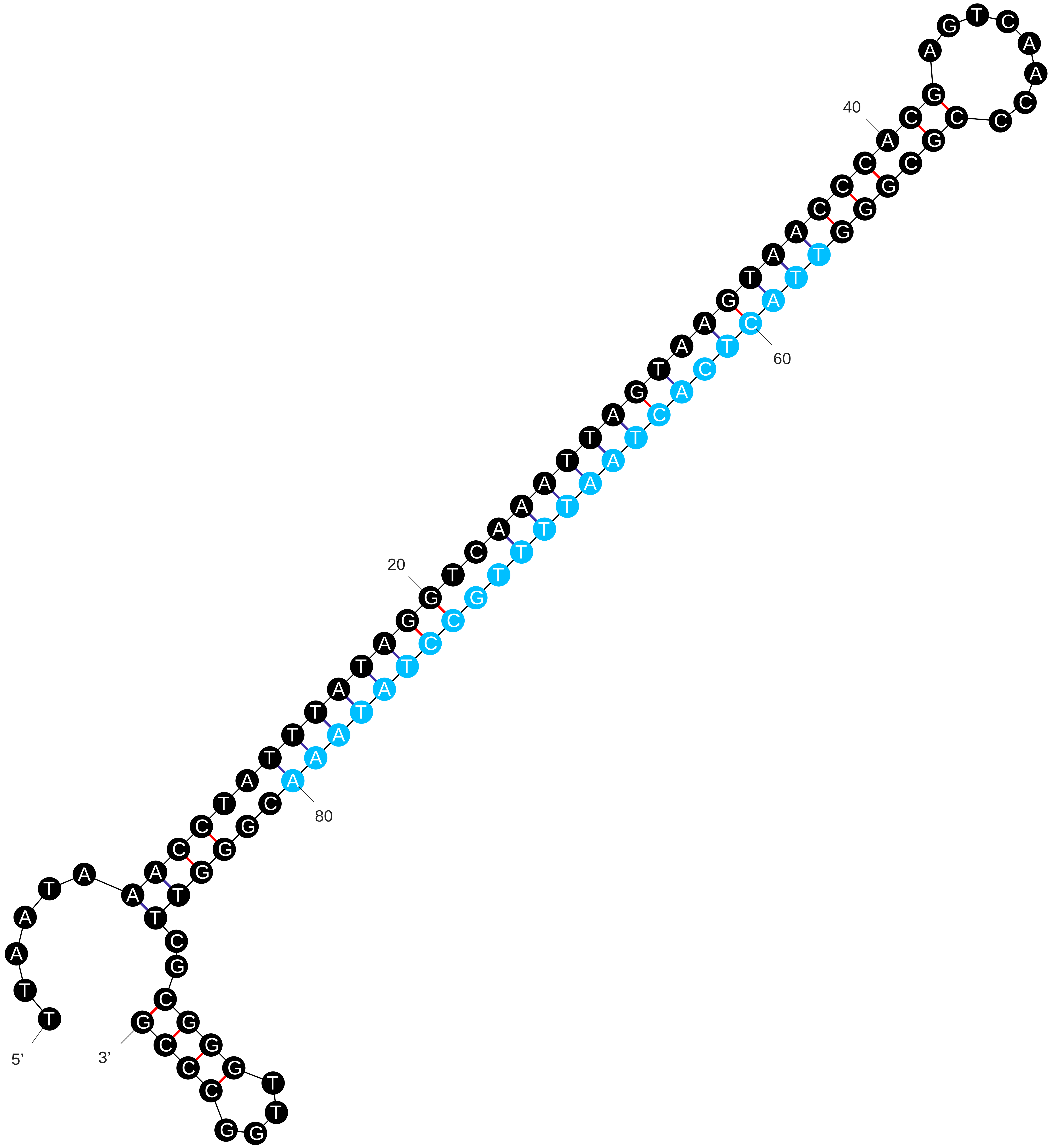

dG = -45.90 osa-cand034

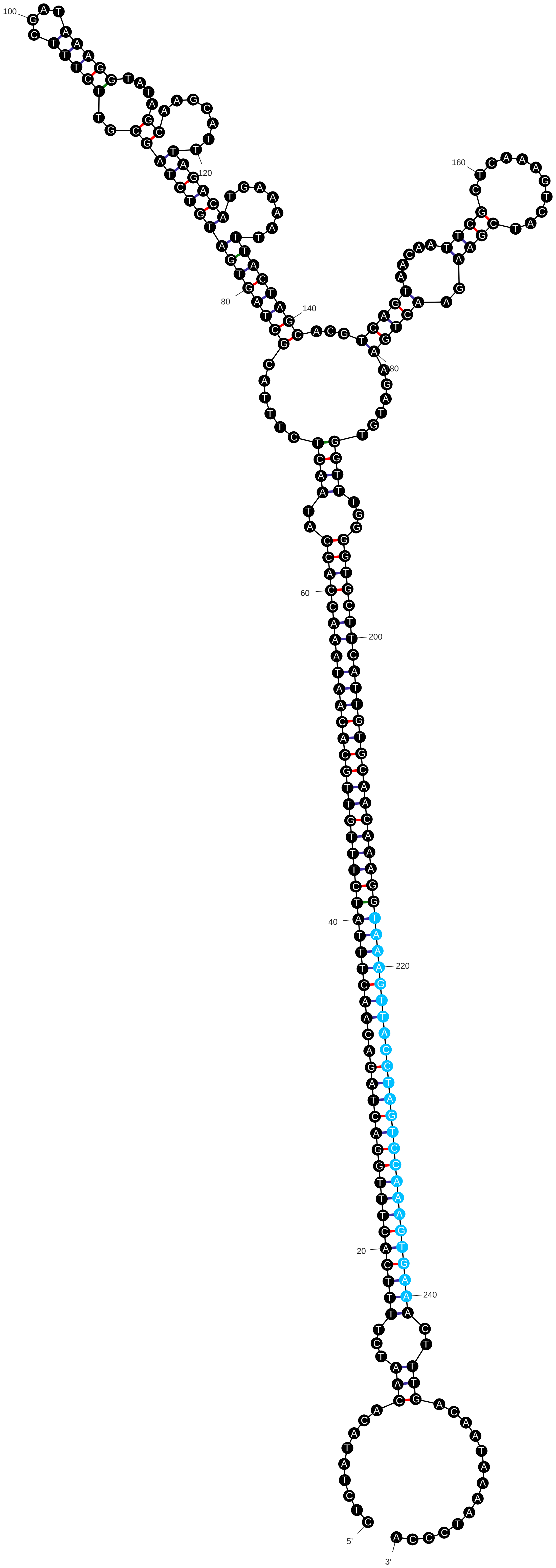

dG = -87.00 osa-cand035a

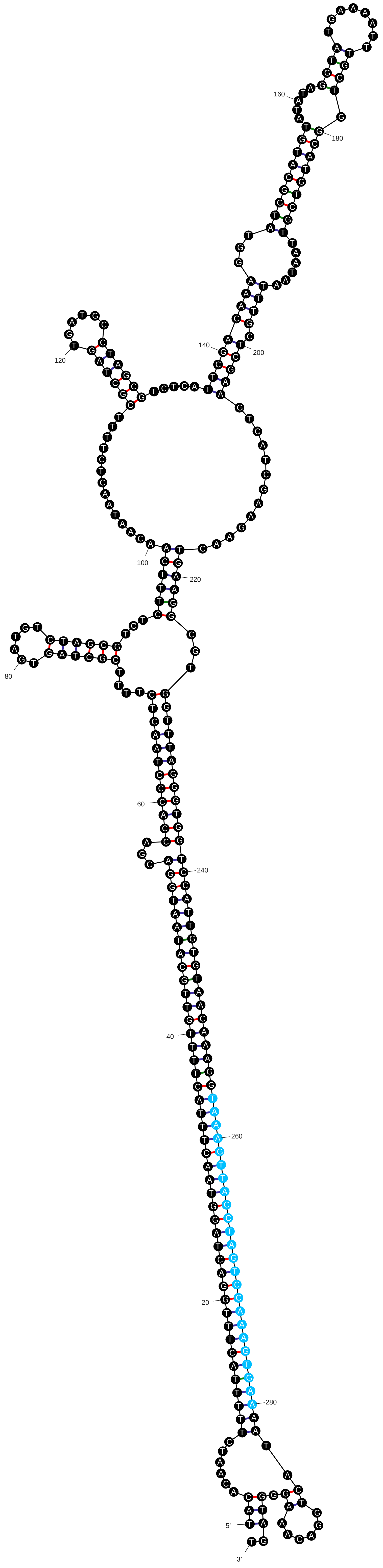

dG = -121.30 osa-cand035b

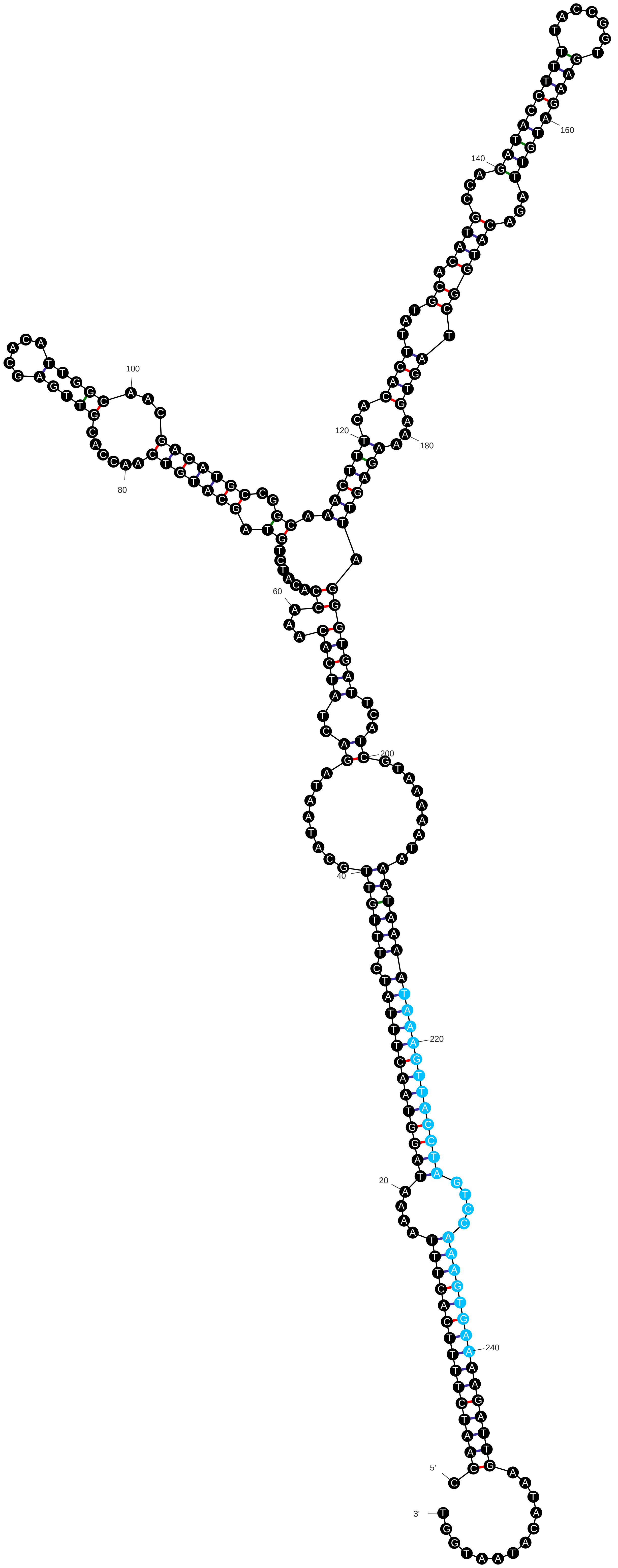

dG = -71.60 osa-cand035c

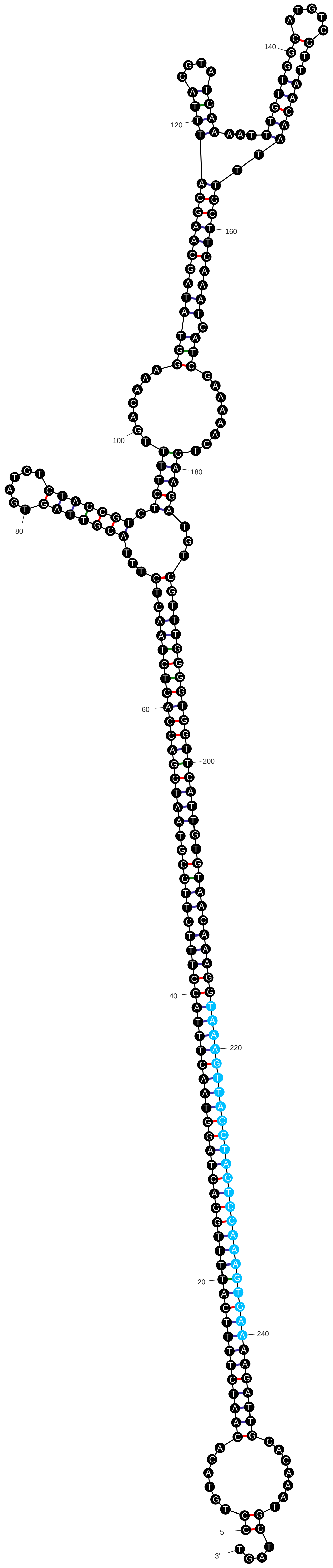

dG = -108.20 osa-cand035d

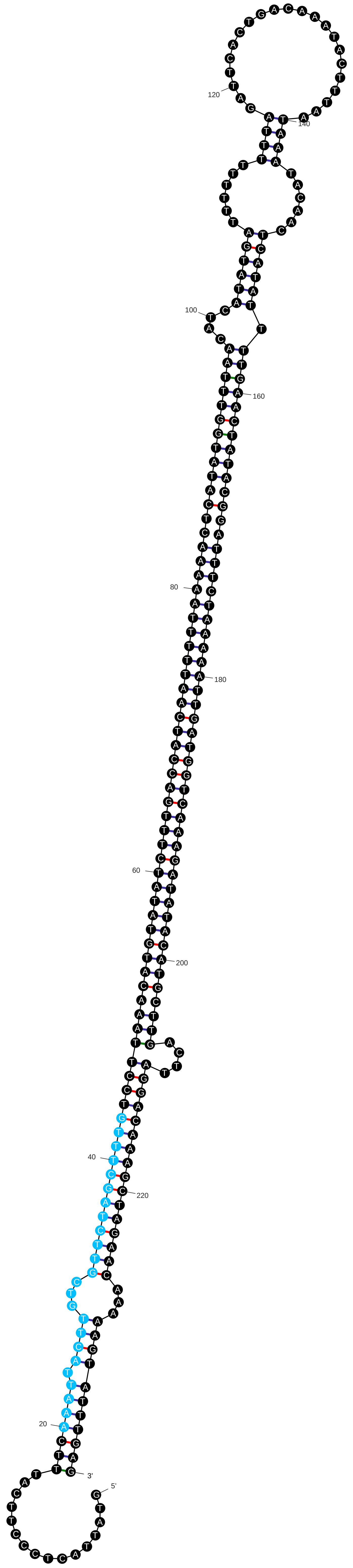

dG = -87.10 osa-cand036

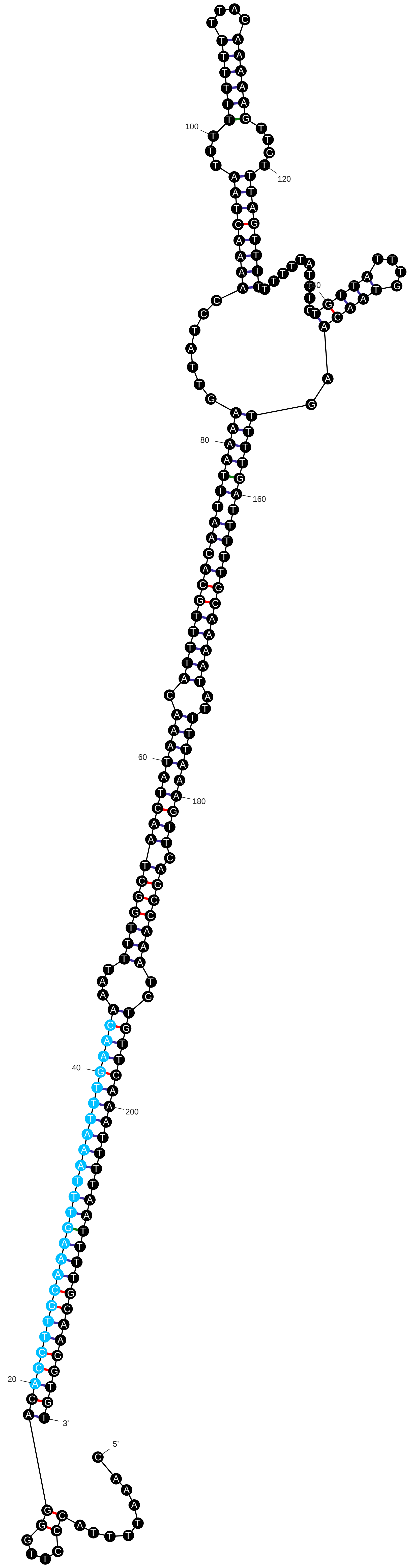

dG = -66.90 osa-cand037

Output of sir_graph (©)
mfold_util 4.6

Created Fri Jun 15 02:00:41 2012

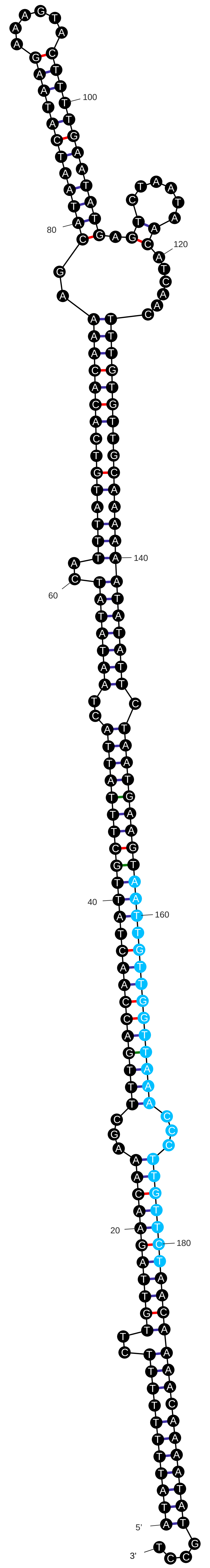

dG = -56.50 osa-cand038

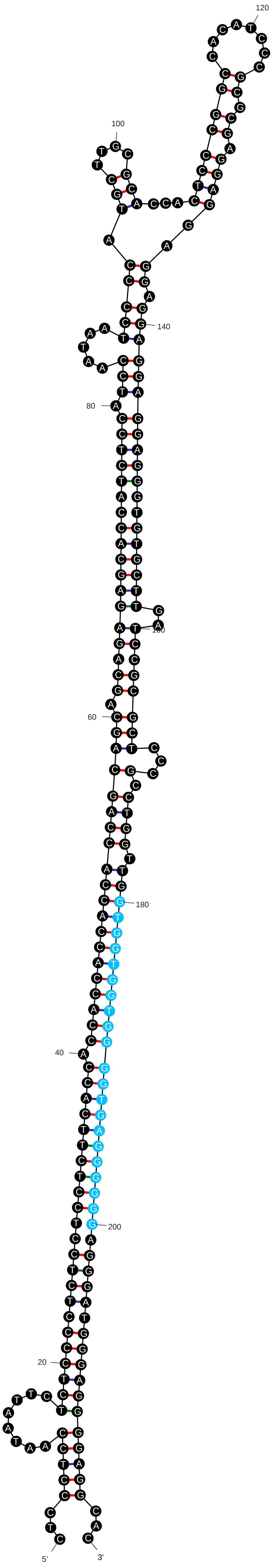

dG = -114.90 osa-cand039

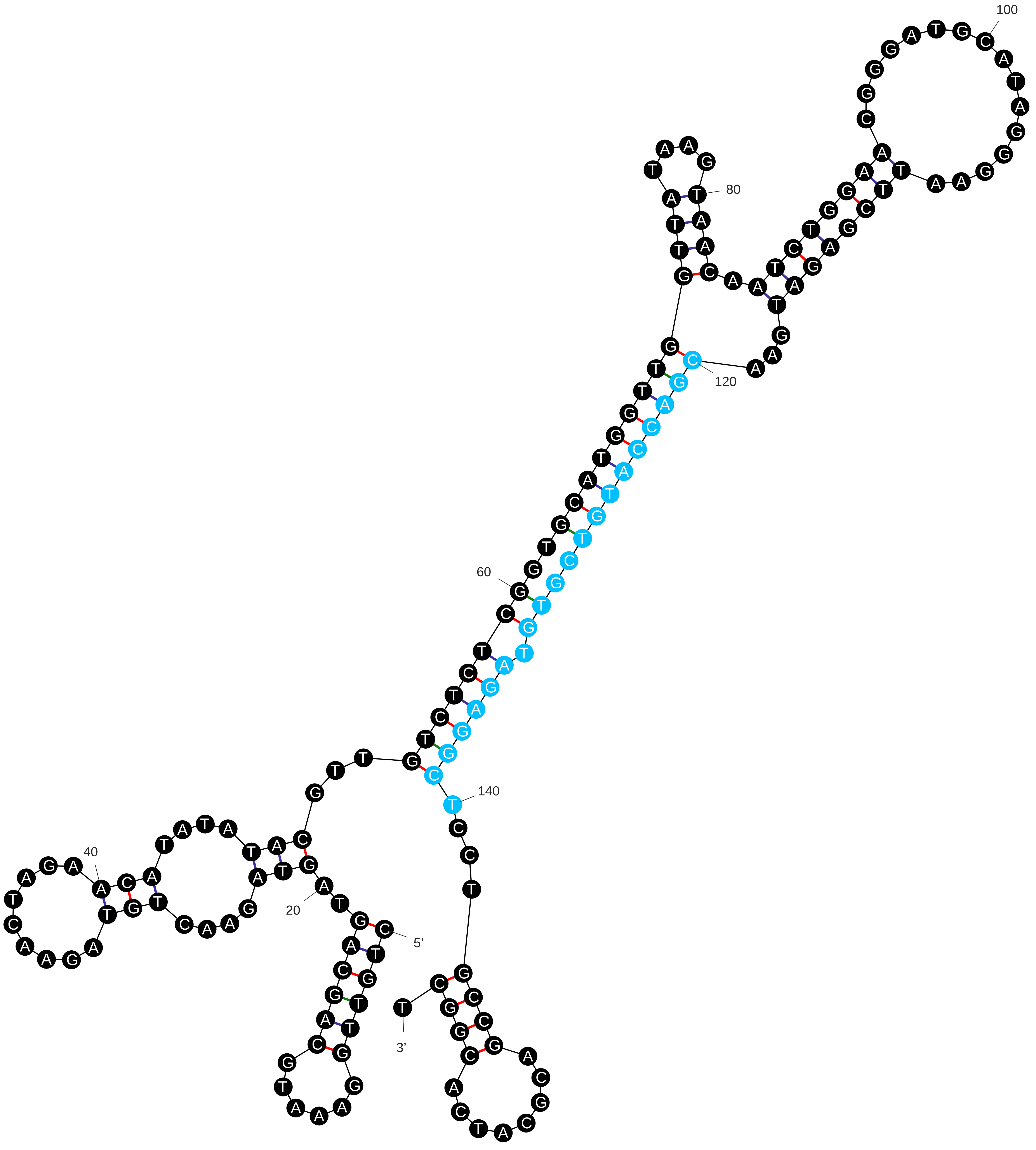

dG = -38.40 osa-cand040

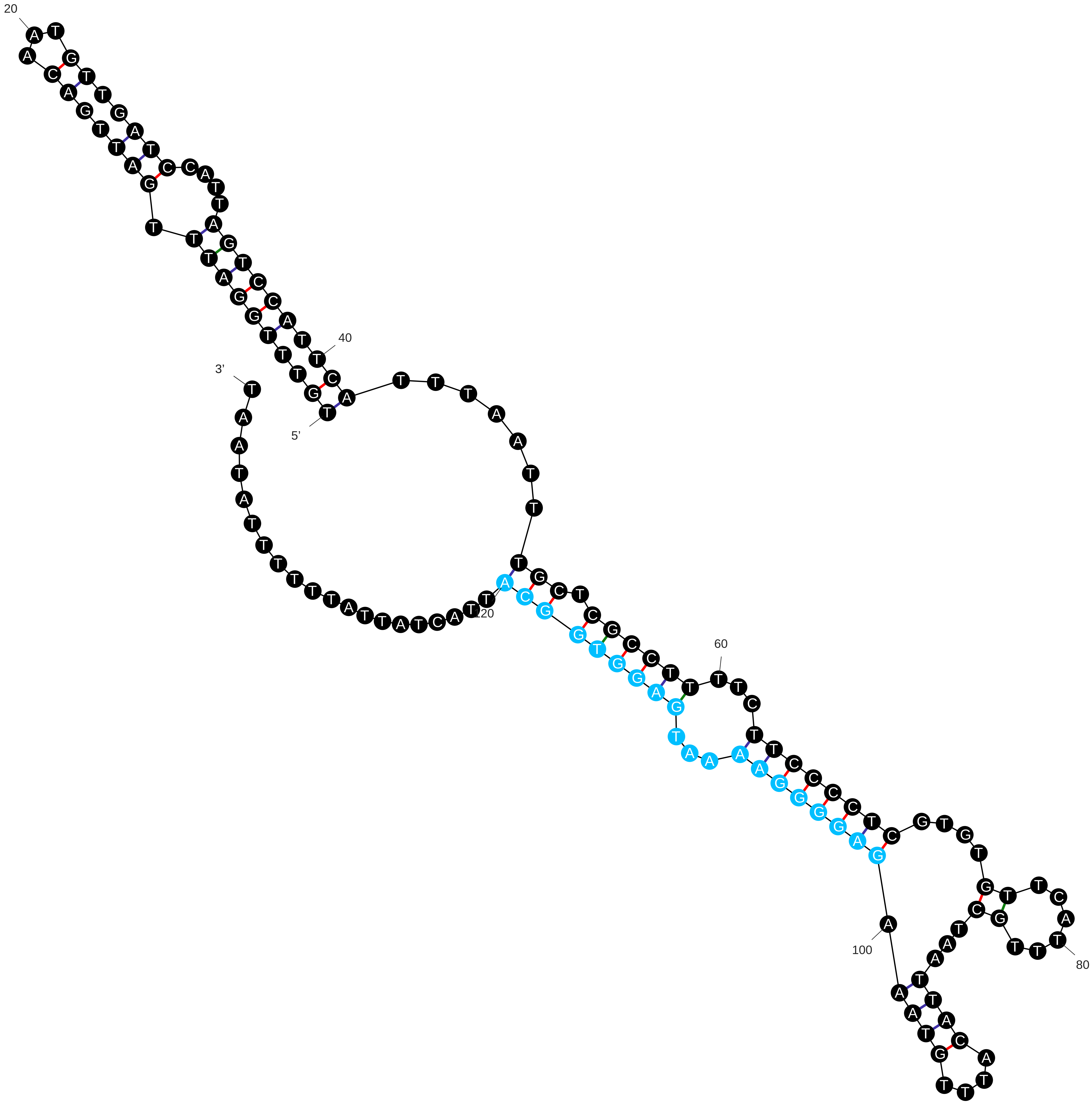

dG = -38.90 osa-cand041

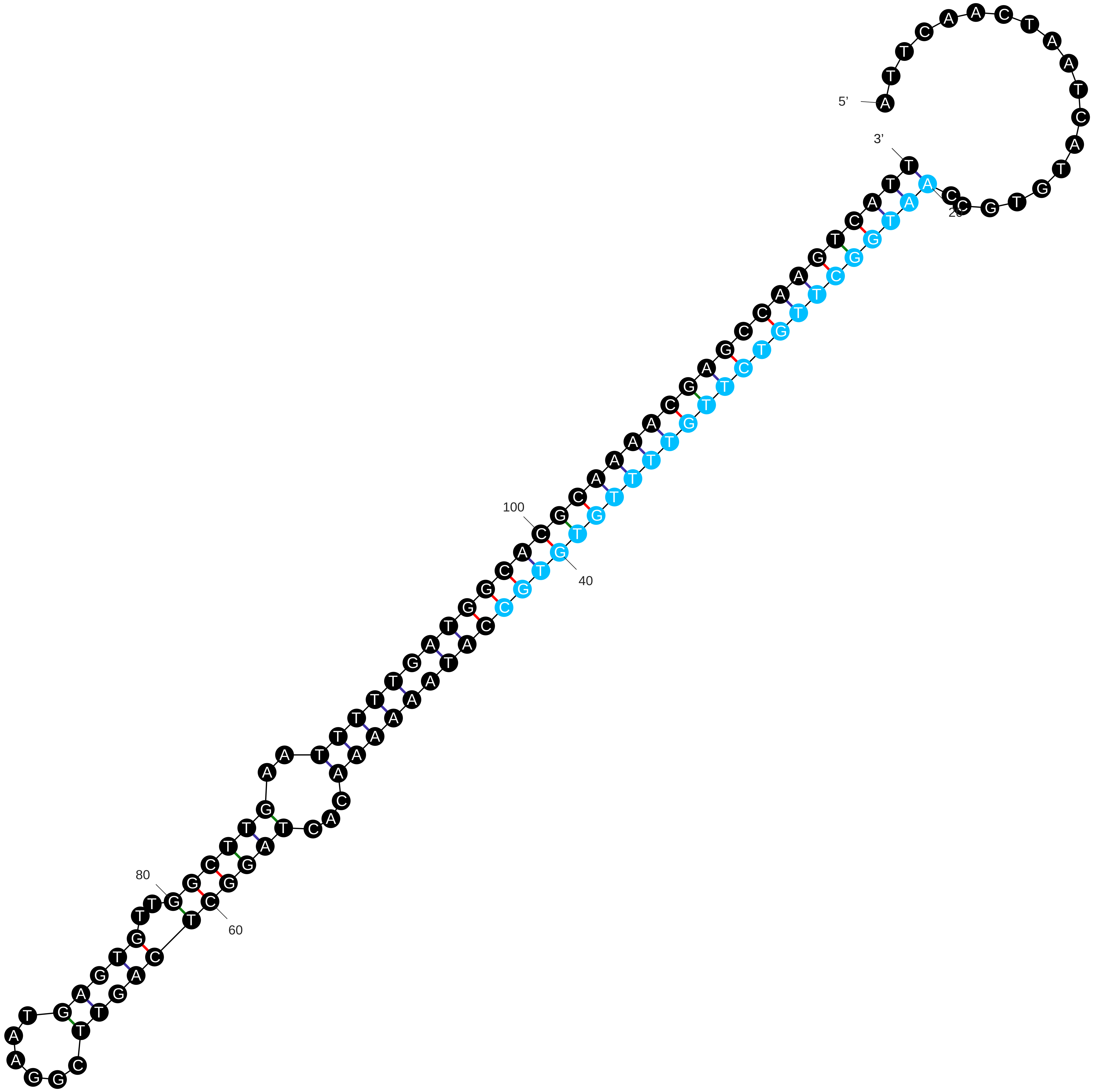

dG = -45.00 osa-cand042

Output of sir_graph (©)
mfold_util 4.6

Created Fri Jun 15 02:26:56 2012

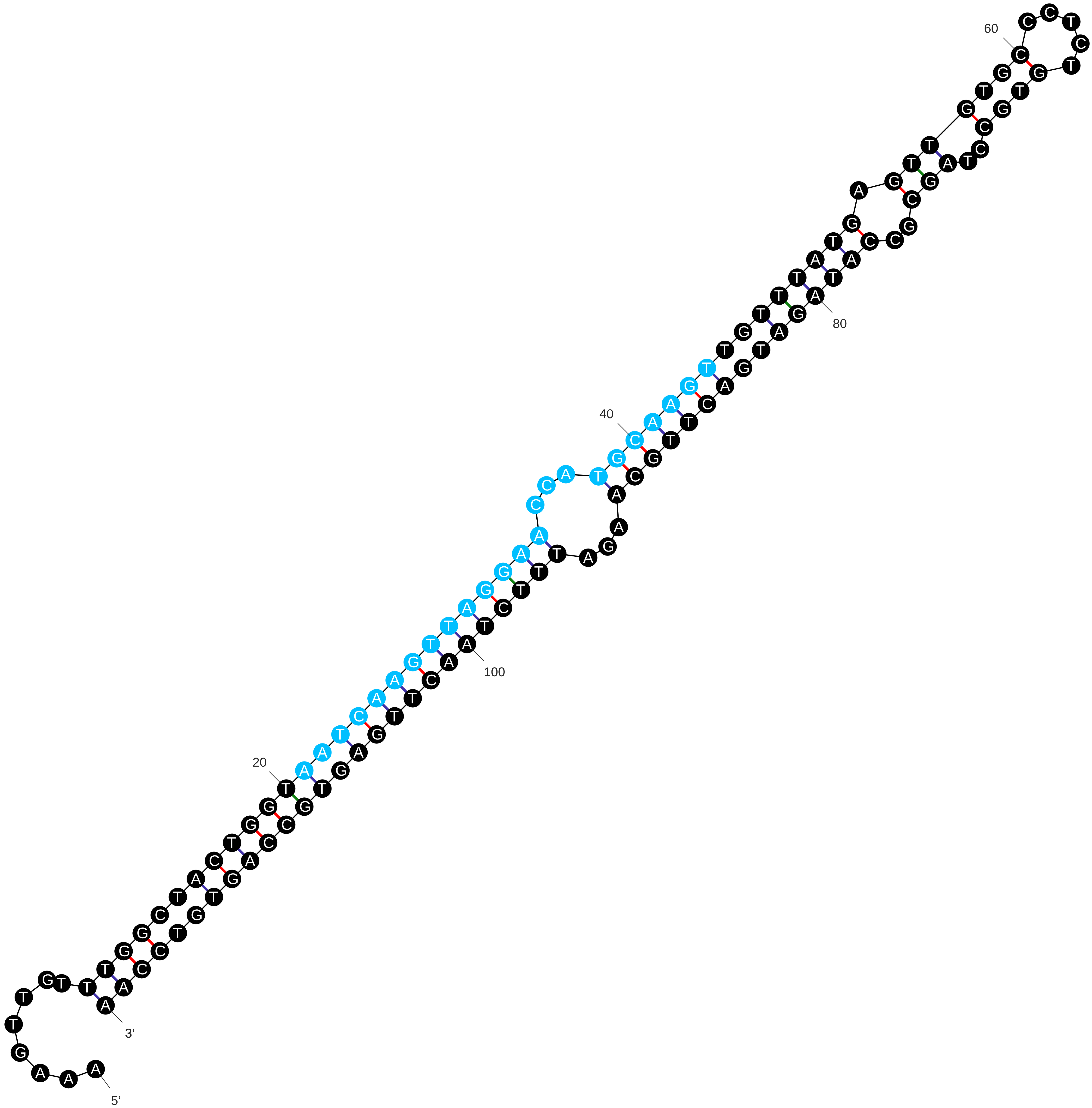

dG = -51.40 osa-cand043

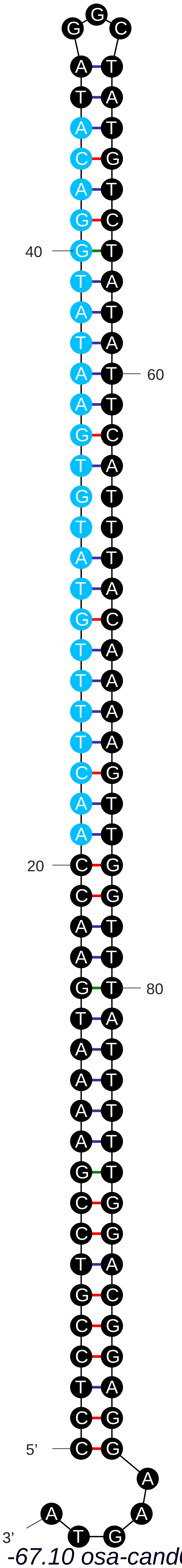

dG = -67.10 osa-cand044

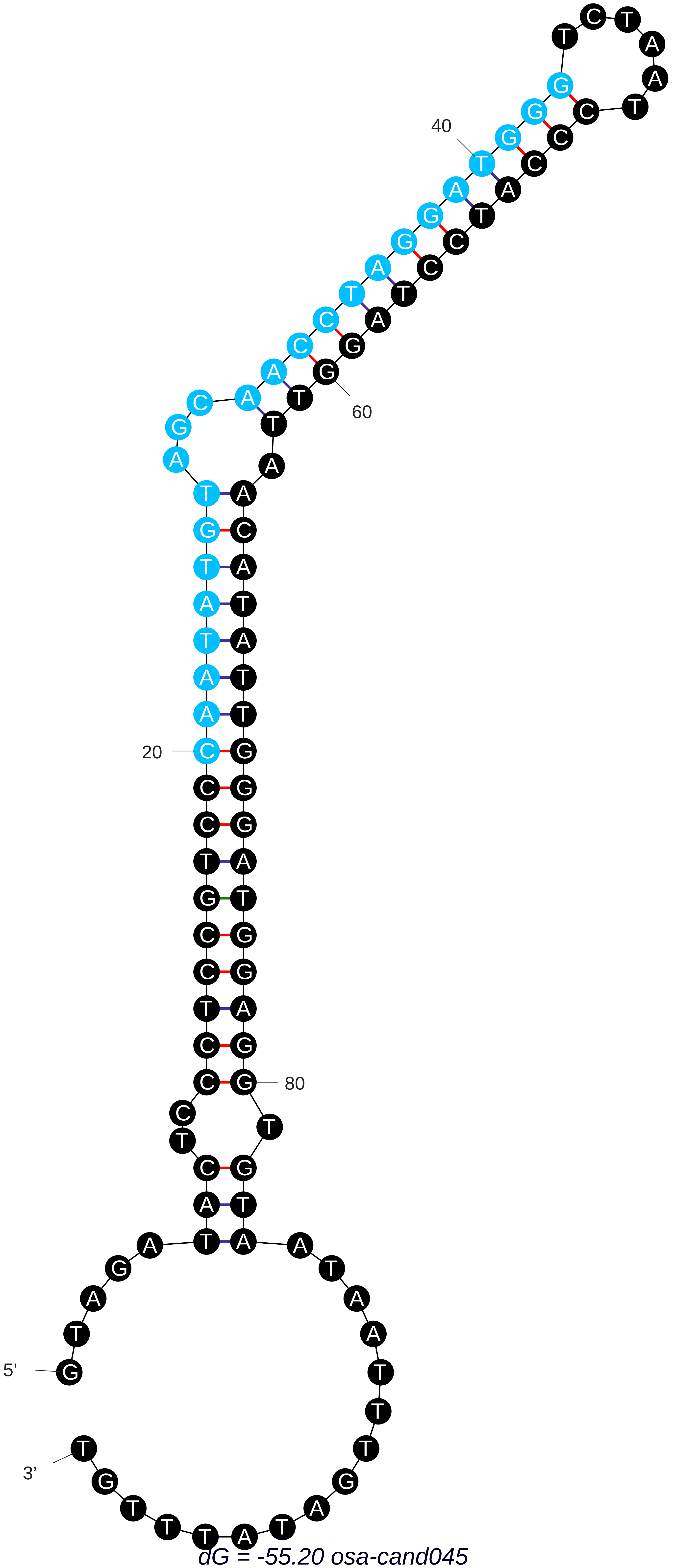

dG = -55.20 osa-cand045

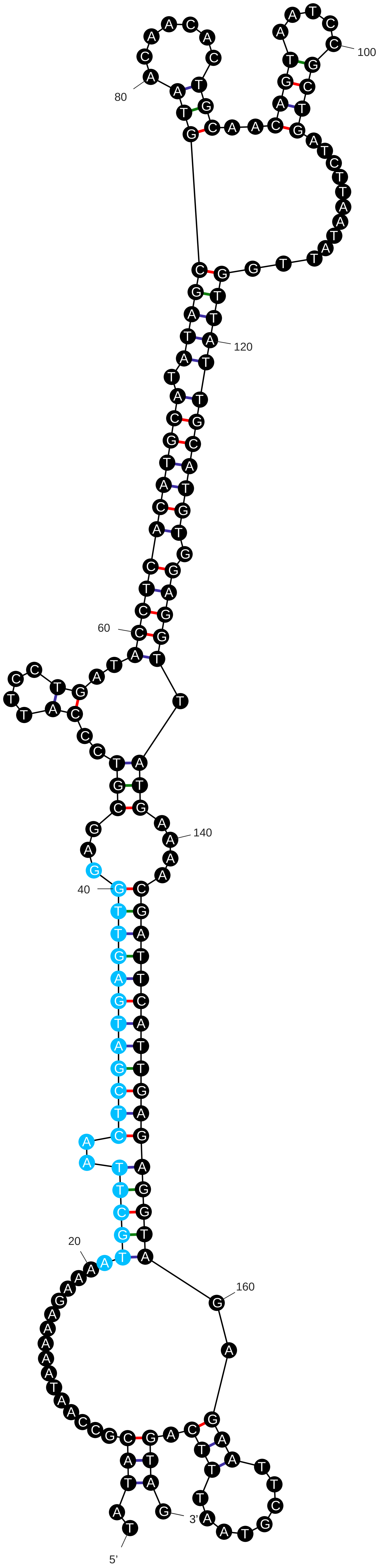

dG = -39.40 osa-cand046

Output of sir_graph (©)
mfold_util 4.6

Created Fri Jun 15 02:39:43 2012

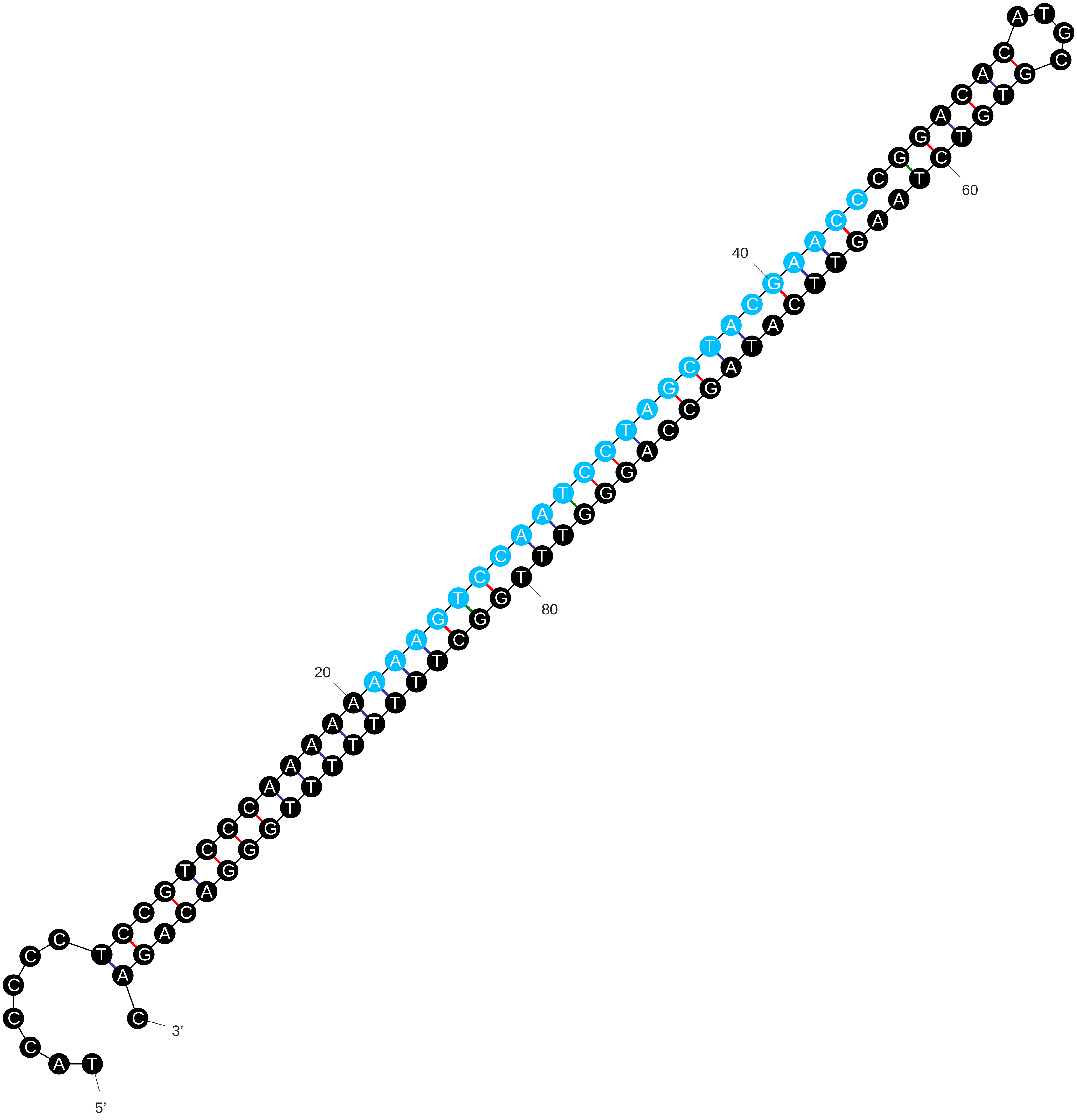

dG = -51.10 osa-cand047

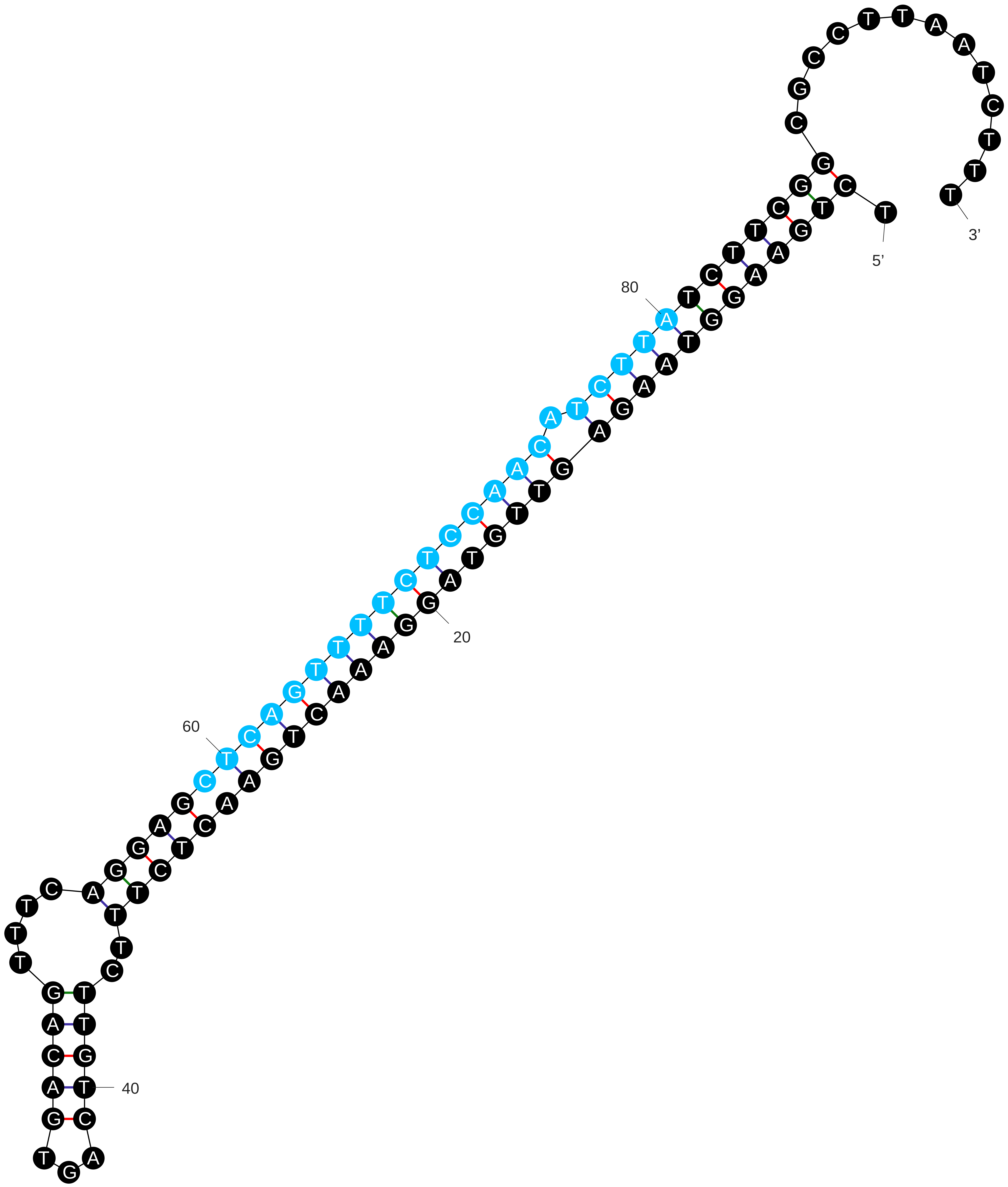

dG = -40.20 osa-cand048

Output of sir_graph (©)
mfold_util 4.6

Created Fri Jun 15 02:42:45 2012

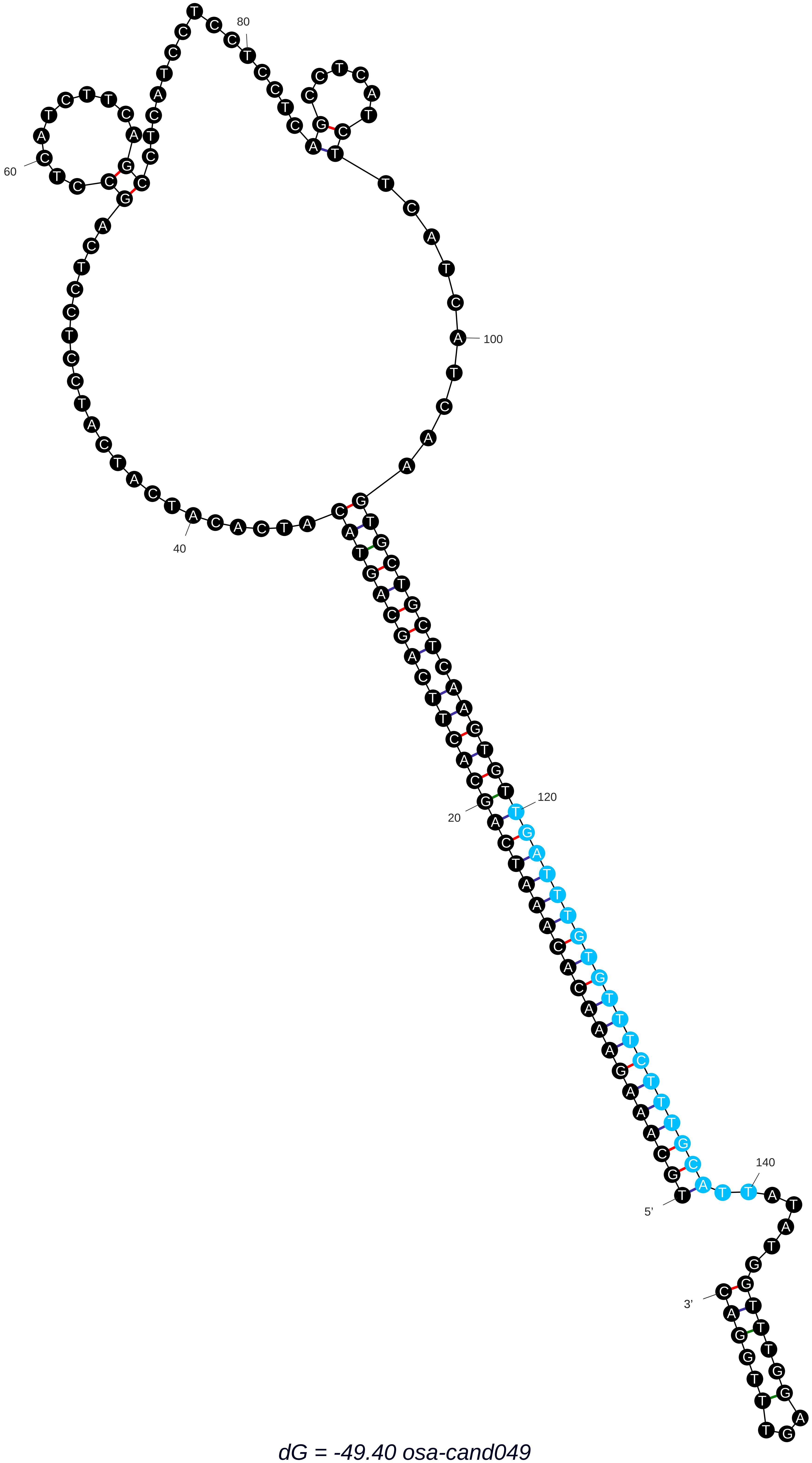

dG = -49.40 osa-cand049

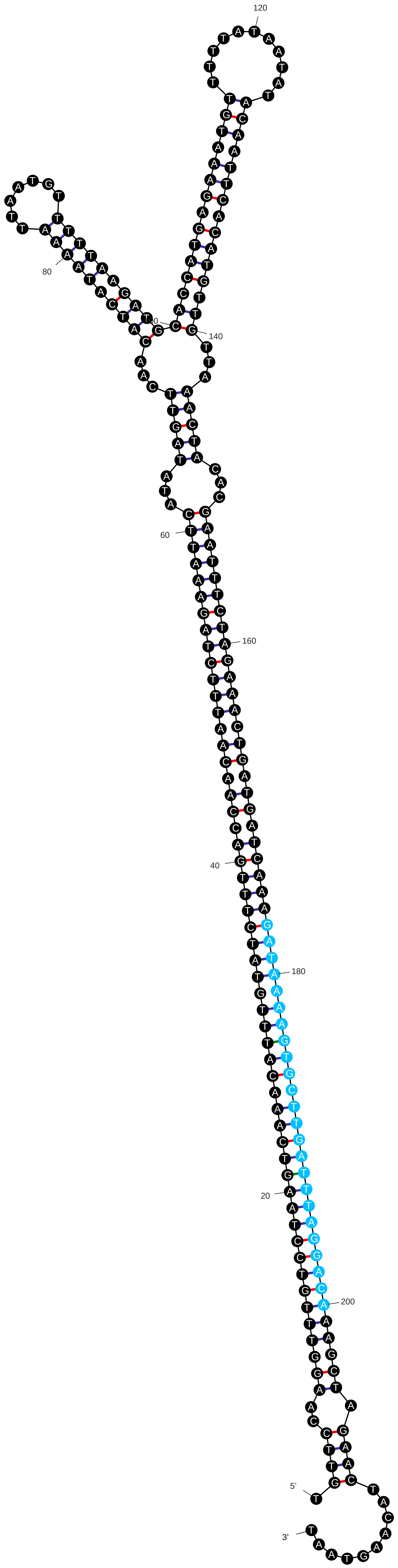

dG = -74.60 osa-cand050

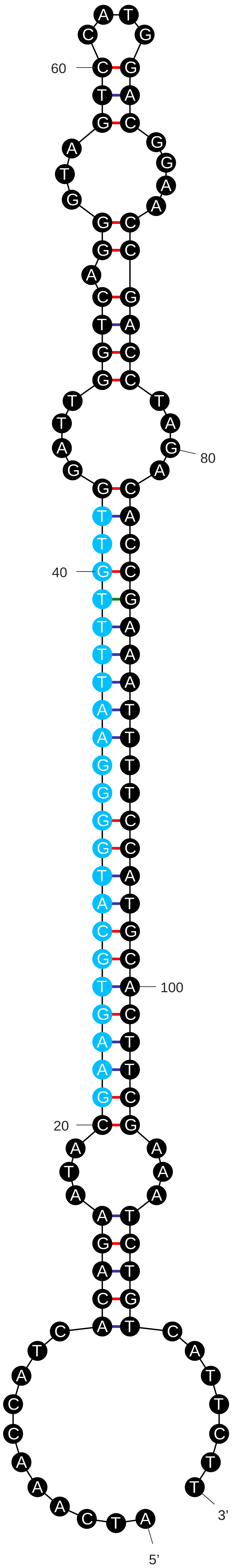

dG = -52.00 osa-cand051

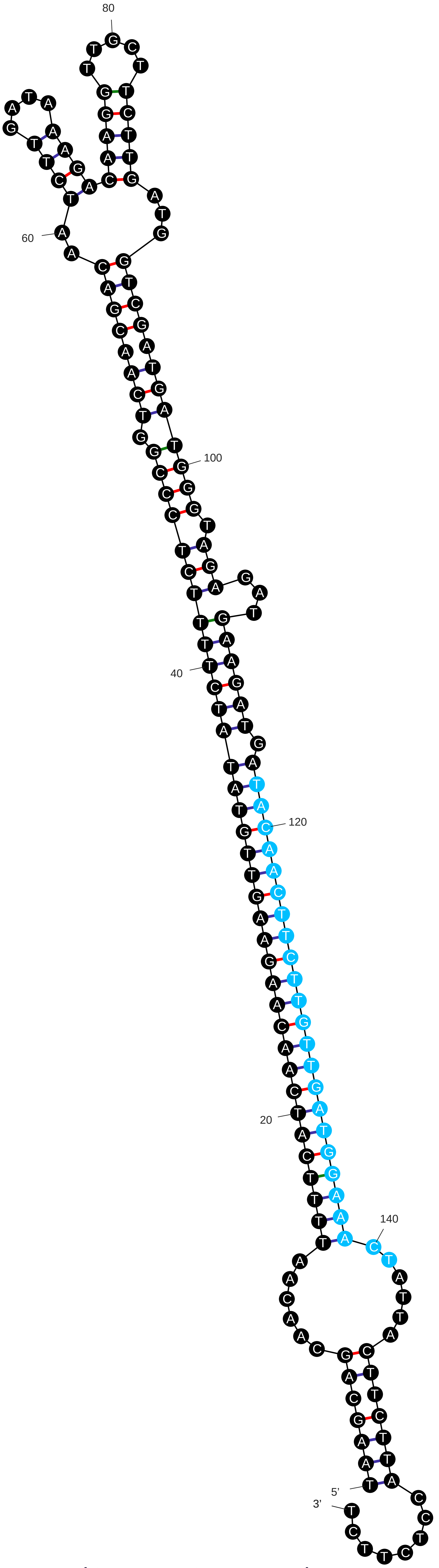

dG = -59.10 osa-cand052a

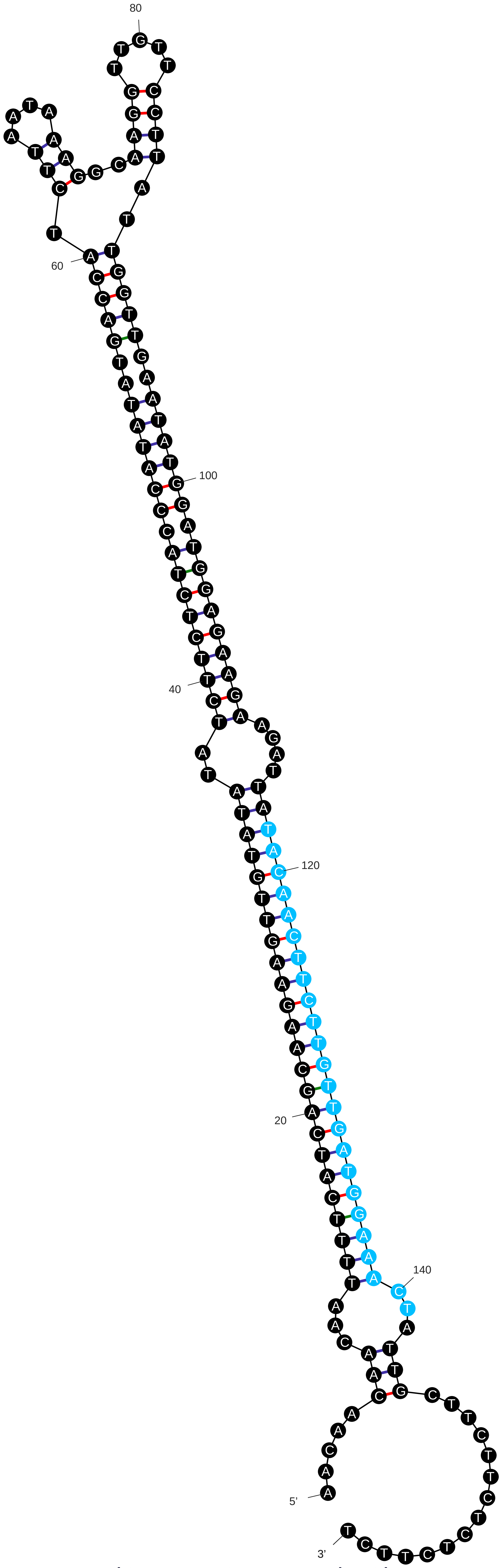

dG = -61.30 osa-cand052b

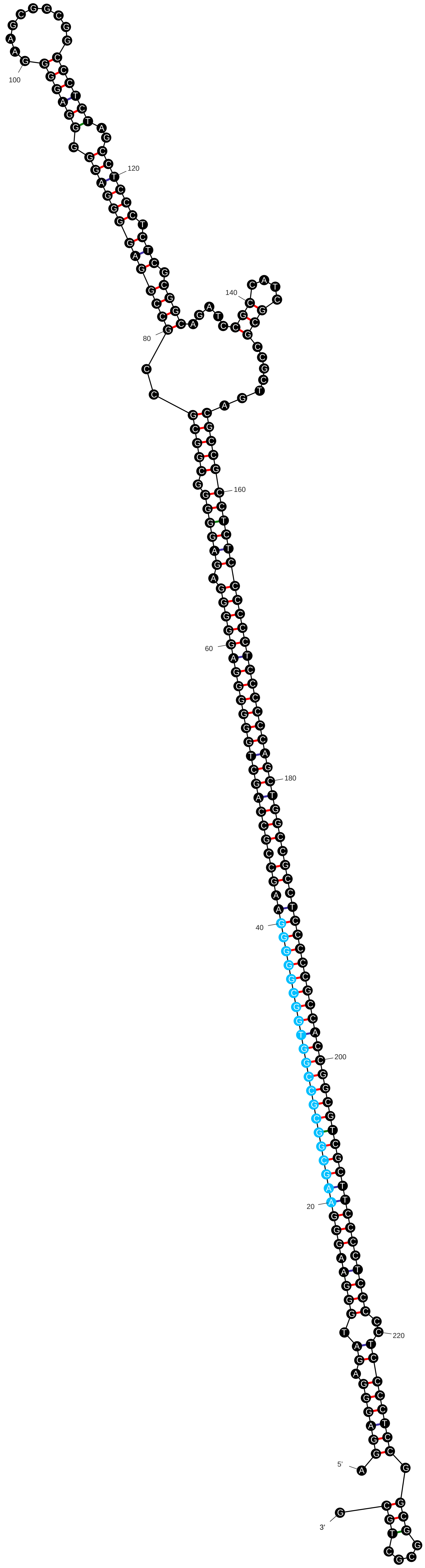

dG = -200.60 osa-cand053

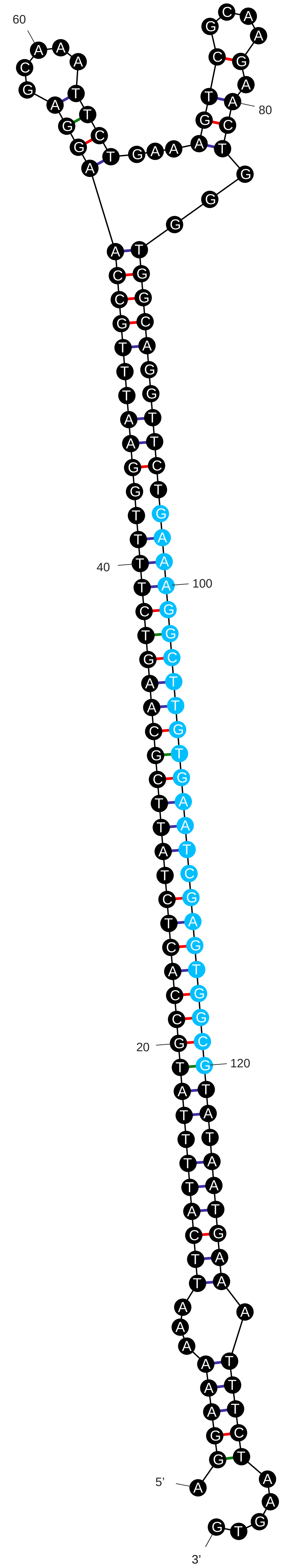

dG = -66.00 osa-cand054

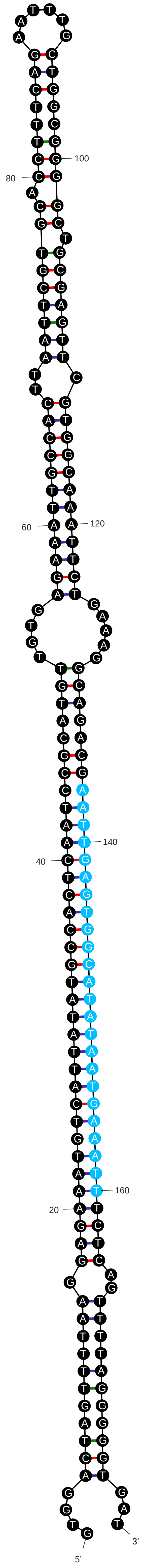

dG = -75.40 osa-cand055

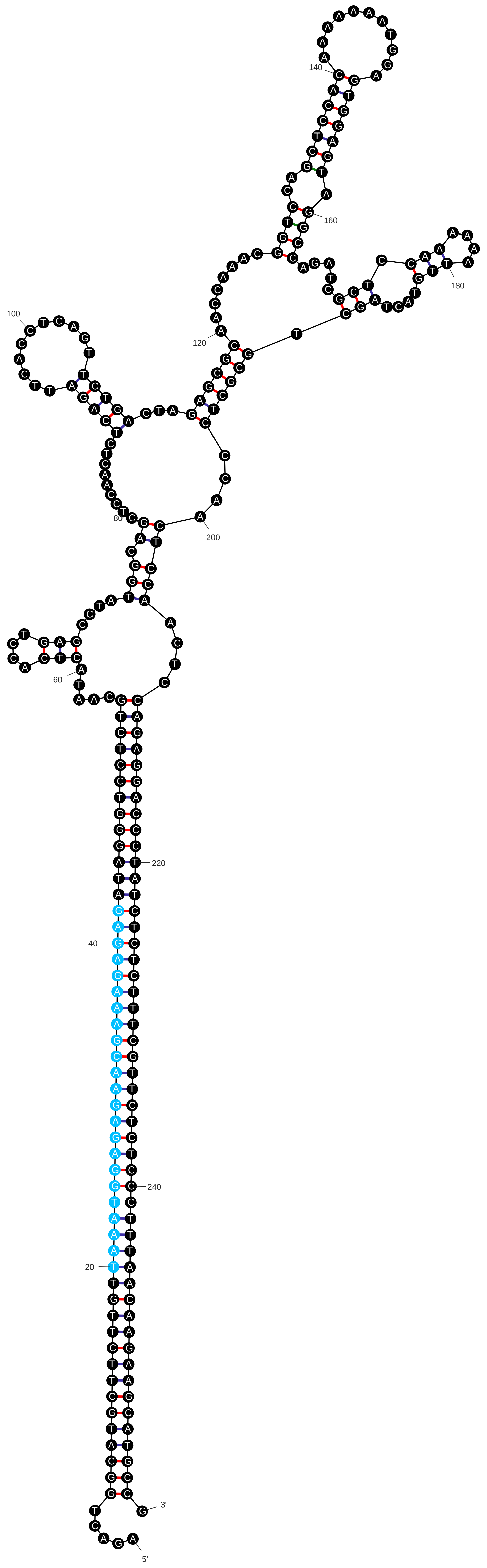

dG = -130.80 osa-cand056

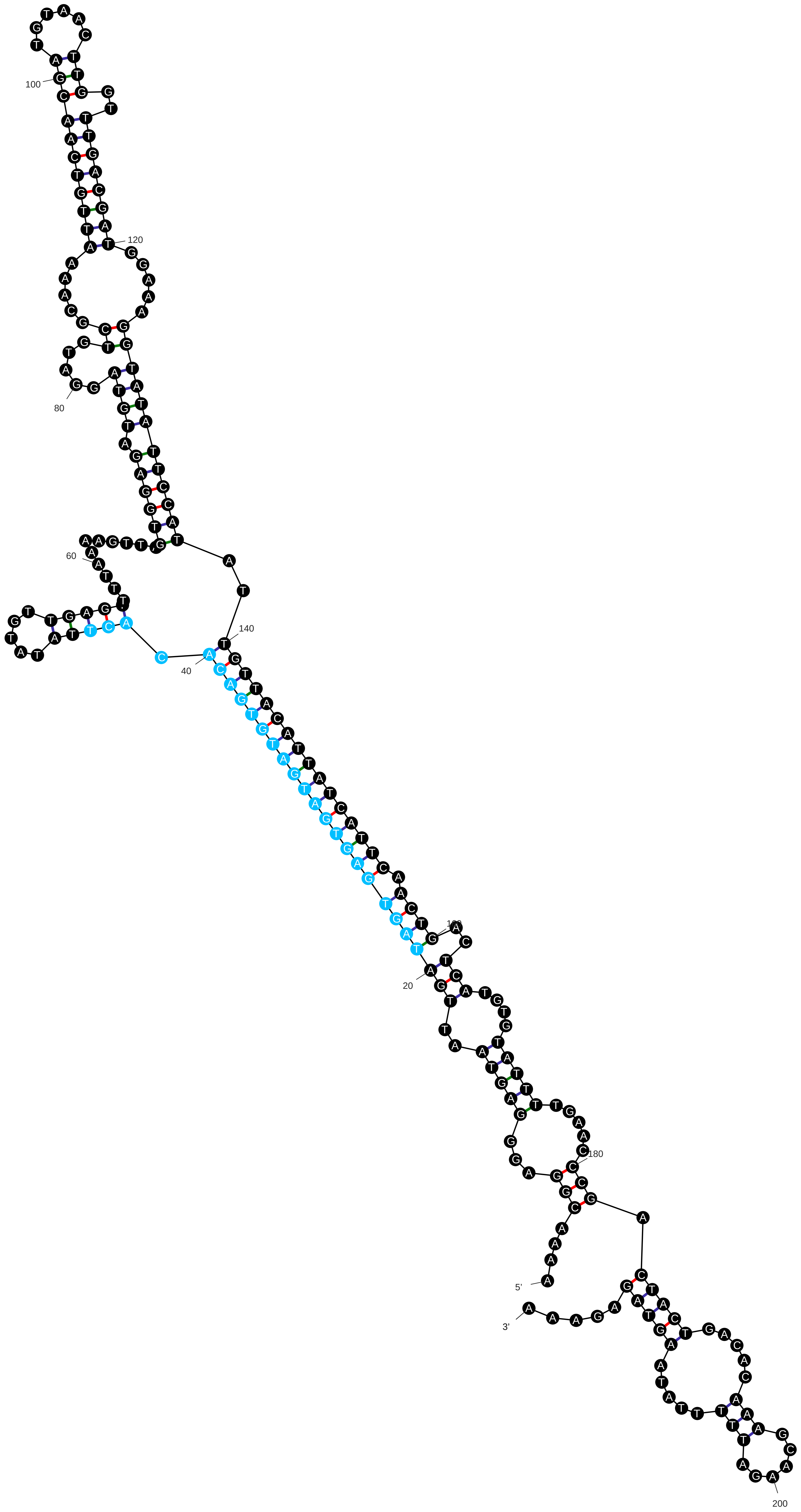

dG = -47.00 osa-cand057

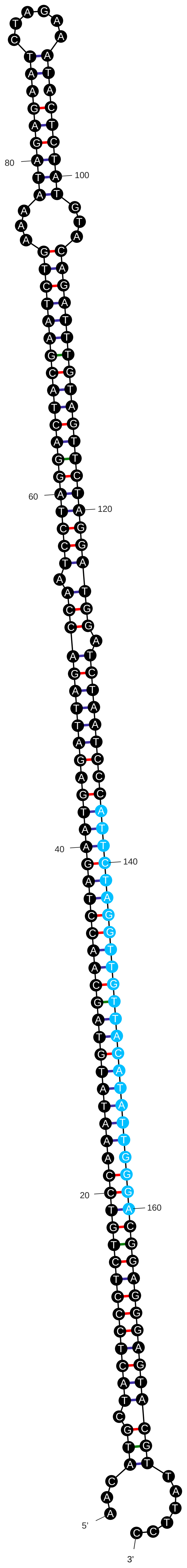

dG = -110.60 osa-cand058

Output of sir_graph (©)
mfold_util 4.6

Created Fri Jun 15 03:32:04 2012

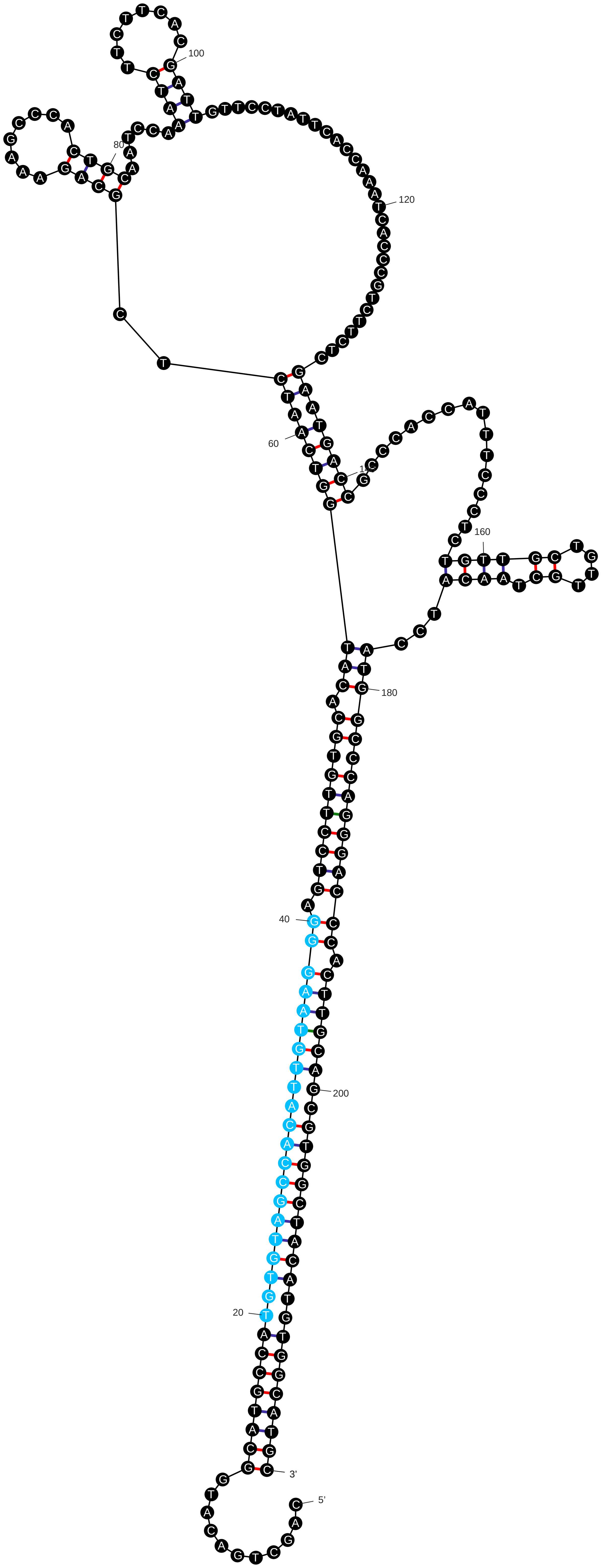

dG = -80.20 osa-cand059

Output of sir_graph (©)
mfold_util 4.6

Created Fri Jun 15 03:33:44 2012

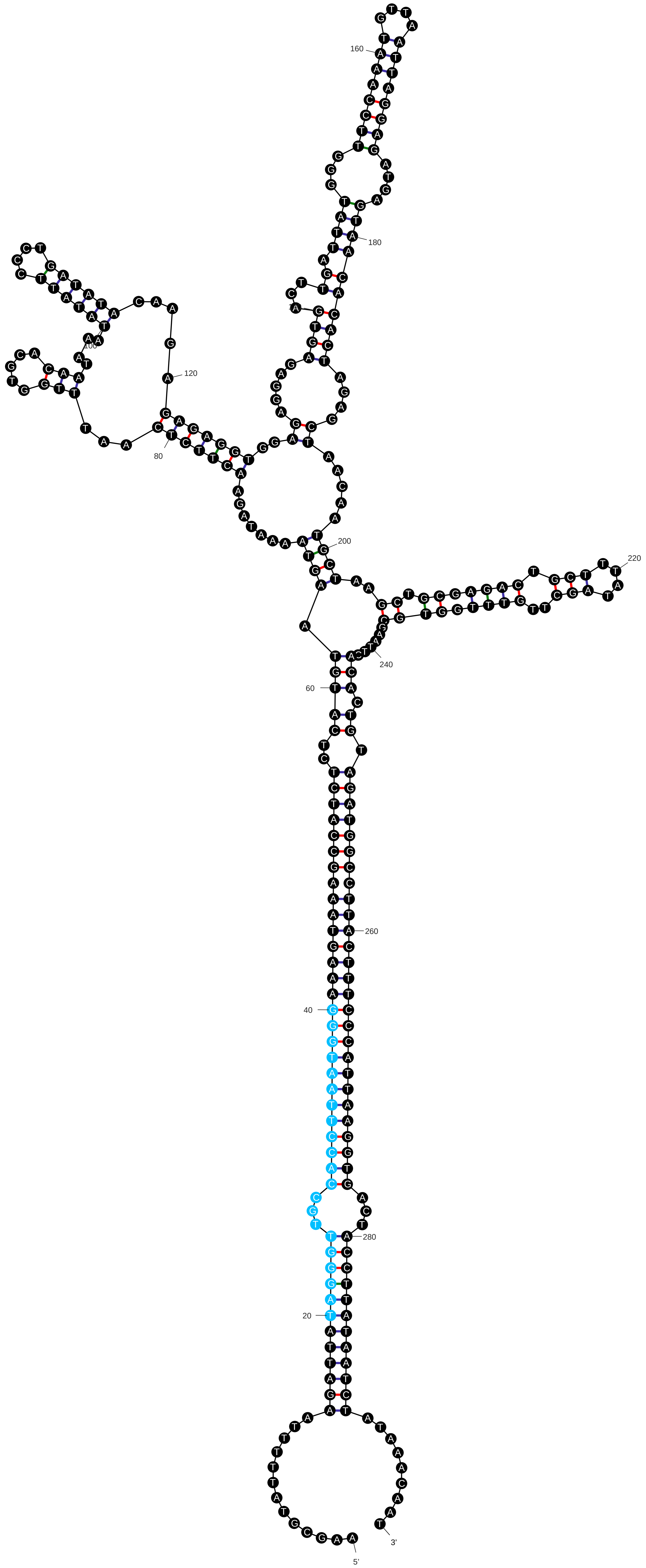

dG = -91.50 osa-cand060

Output of sir_graph (©)
mfold_util 4.6

Created Fri Jun 15 03:35:31 2012

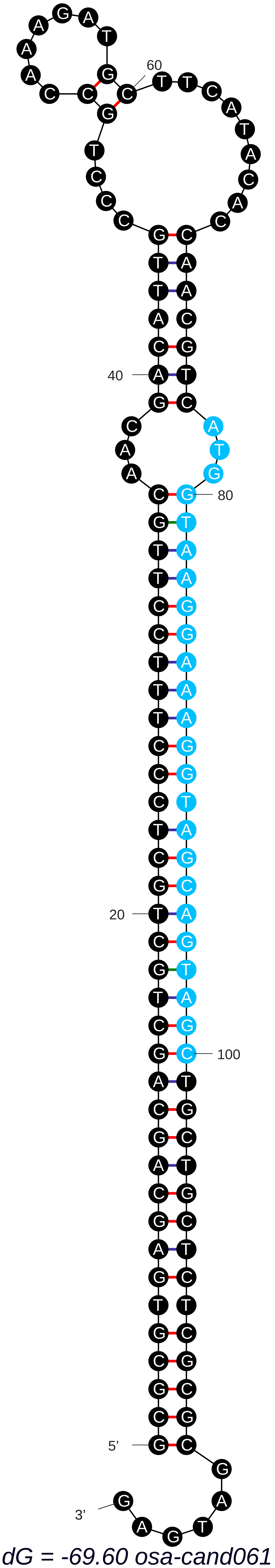

dG = -69.60 osa-cand061

Created Fri Jun 15 03:36:58 2012

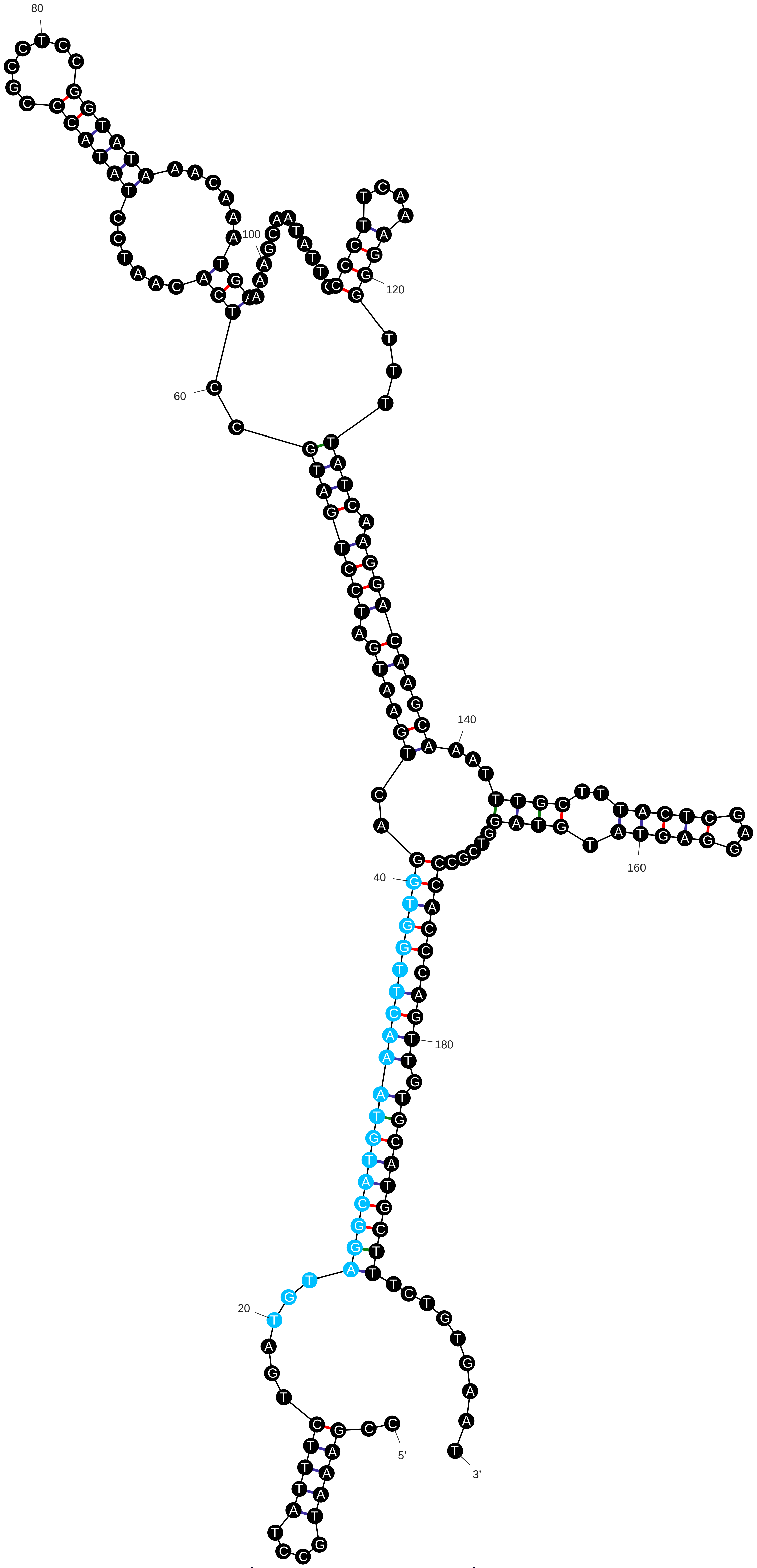

dG = -48.00 osa-cand062
