# Supplementary material for: High throughput sequencing reveals novel and abiotic stress-regulated microRNAs in the inflorescences of rice
Source: BMC Plant Biol. 2012 Aug 3;12:132. doi: 10.1186/1471-2229-12-132 (PMC3431262; doi:10.1186/1471-2229-12-132)
Supplement: Additional file 4 — Small RNA reads from four libraries that were mapped to the precursors of new candidate miRNAs. On each map, the first line contains the miRNA ID. The second line contains the miRNA precursor sequence, with mature miRNA region in red. The third line contains the notation of secondary structure with parentheses denoting base-pairing and dots denoting mismatches or bulges. The number on the right is the free energy. Every line starting from line 4 contains the sequence, mapping position, and count of a mapped unique small RNA read. [file 1471-2229-12-132-S4.pdf]

```
>osa-cand001
TCCACCATCGTCGTCGTCGTCGTCGCCACCCCTCTCCCACCATCGTCTCCGCCATCGTTGTCGCCGTCGCCTCGCGCCACCACGGTCGCTGCCGCCTCCGACTTCCCCGTCGCCTCGCGTCGTCGTCGCCGTCGCCCGCCCCCGCCACCATCTCCGGCCCCCCAAACCCTAGCCGCCGCCCGCTCGTTCCGACGGGGATTCGGAGGGGGTCGAAGGGAGGAAGAGGCGGTGGCGGTCGGCGAAGAGGACGACGATGGCGACGGCGGCAGCGGCG
....((((((((((.((.(((((((((((((((((((..((.(((.....((.(((((.((.((((......)).)).)).))))).)).(((.(((((((..(((((((((....(((.((.((....)).))..)))....((.........((((............)))).......))......))))))))).))))))).)))))).))..)).))))).)))))))).)))))).)).)))))))))))...((.((....)).)) (-133.7)
TCCACCATCGTCGTCGTCGTCG.................................................................................................................................................................................................................................................................... 1
........CGTCGTCGTCGTCGTCGCC....................................................................................................................................................................................................................................................... 1
...........................ACCCCTCTCCCACCATCGTCTC................................................................................................................................................................................................................................. 1
......................................................................................................TTCCCCGTCGCCTCGCGTCGTCGTCG.................................................................................................................................................. 1
........................................................................................................................................................................................................................................CGGTCGGCGAAGAGGACGACGA.................... 11
...........................................................................................................................................................................................................................................TCGGCGAAGAGGACGACGATG.................. 3
............................................................................................................................................................................................................................................CGGCGAAGAGGACGACGATGG................. 1
..............................................................................................................................................................................................................................................GCGAAGAGGACGACGATGGCGAC............. 1
```

```
>osa-cand002
TAGATGGACTTTCAGCACAAAAGTACATACTCACAAGCACCGAAAGAGCCCTCCCTTGTAACTCTGACATCTTTCGGTGCTTGTGACTATGTATTTTTGT
................((((((((((((((.(((((((((((((((((....................))))))))))))))))).)))))))))))))) (-42.8)
.............AGCACAAAAGTACATACTCACAAG...............................................................  2
.................CAAAAGTACATACTCACAAGCACC...........................................................  5
...................AAAGTACATACTCACAAGCACCGA.........................................................  21
....................AAGTACATACTCACAAGCACCGA.........................................................  1
....................AAGTACATACTCACAAGCACCGAA........................................................  8
.....................AGTACATACTCACAAGCACCGAAA.......................................................  2
......................GTACATACTCACAAGCACCGAAAG......................................................  1
.......................TACATACTCACAAGCACCGAAAGA.....................................................  2
.........................................................GTAACTCTGACATCTTTCGGTGC....................  1
...............................................................CTGACATCTTTCGGTGCTTG.................  1
.......................................................................TTTCGGTGCTTGTGACTATGT........  1
.......................................................................TTTCGGTGCTTGTGACTATGTATT.....  3
.........................................................................TCGGTGCTTGTGACTATGTAT......  1
.........................................................................TCGGTGCTTGTGACTATGTATTTT...  1
.............................................................................TGCTTGTGACTATGTATTTTT..  1
```

```
>osa-cand003
TTTAGGTCAACATTTGGTCCAACTTTTCAAACTTTGATCATCAATTATTTCTCAATGCTTAGTTTAAAAAGGAAAAAAATGGTAAGCATAGATTTTCCTTGAAAAGCACTATCATATTTTCTTTATGATTTTATAAACTTATTCTATTATAAAATTGATGGTCAAAATTTCAAAAGTTTG
....(..(((...)))..).(((((((.(((.(((((((((((((................((((...((((((((..(((.....)))...))))))))...))))...((((((.......))))))........................))))))))))))).))).))))))).. (-36.8)
....................AACTTTTCAAACTTTGATCA............................................................................................................................................ 1
....................AACTTTTCAAACTTTGATCATCA......................................................................................................................................... 2
....................AACTTTTCAAACTTTGATCATCAA........................................................................................................................................ 23
........................................TCAATTATTTCTCAATGCTTAGTTT................................................................................................................... 1
```

```
>osa-cand004
TGTTCATAGTTAGTATTCTATTGGATTTTCTACCATGCAATTTAAATGCCATGTCATGAGGACAACAATTGGAGACATATTTGGTCTCTCTTACAGGGCATGGGCTGCACAACTGATAGTATAAAAATGATGTTTGAATTGCATGGCAGAGGATTCAGTATCTAAGATGCCATTGCATTC
..................((((((((..(((.(((((((((((((((.(((((((..((((((......((....))......))).)))......)))))))((((((....)).)))).............))))))))))))))).)))..)))))))).....(((((....))))). (-56.1)
...............TTCTATTGGATTTTCTACCATGCA............................................................................................................................................. 21
................TCTATTGGATTTTCTACCATGCAA............................................................................................................................................ 12
.................CTATTGGATTTTCTACCATGCAAT........................................................................................................................................... 2
..................TATTGGATTTTCTACCATGCAATT.......................................................................................................................................... 16
....................TTGGATTTTCTACCATGCAATTTA........................................................................................................................................ 2
........................................................................................................................................AATTGCATGGCAGAGGATTCAGT..................... 1
........................................................................................................................................AATTGCATGGCAGAGGATTCAGTA.................... 23
.........................................................................................................................................ATTGCATGGCAGAGGATTCAGTAT................... 7
..........................................................................................................................................TTGCATGGCAGAGGATTCAGTAT................... 3
..........................................................................................................................................TTGCATGGCAGAGGATTCAGTATC.................. 1
...........................................................................................................................................TGCATGGCAGAGGATTCAGTATCT................. 2
............................................................................................................................................GCATGGCAGAGGATTCAGTATCT................. 1
.............................................................................................................................................CATGGCAGAGGATTCAGTATCT................. 2
.............................................................................................................................................CATGGCAGAGGATTCAGTATCTA................ 7
.............................................................................................................................................CATGGCAGAGGATTCAGTATCTAA............... 2
```

```
>osa-cand005a
TTAGGGCAAGATTTGGTCAAACTTTTGAAGCCTTGACCATCAATTACTTCTCAGATTCTTAGTTTAAAAACACAAAAAAATGATATGCTTAAGTTTGTCTTGAAAAGTATTATAATAATATCATAAACTTATTAGGTTTATAAACTTATTCTAACATAGAATTGATGGTCAAAGTTCTAAAAGTTTGTCCAAATCTTGTC
....(((((((((((.(((((((((..(((.(((((((((((((.((....(((.....((((((.((((........(((((((..(((((.....)))))...)))))))..(((((.........))))).)))).))))))...))).....))))))))))))))).)))..))))))))).))))))))))) (-65.1)
.....GCAAGATTTGGTCAAACTTTTGA............................................................................................................................................................................ 1
.......AAGATTTGGTCAAACTTTTGAAGC......................................................................................................................................................................... 1
................TCAAACTTTTGAAGCCTTGACCAT................................................................................................................................................................ 1
...................AACTTTTGAAGCCTTGACCATCAA............................................................................................................................................................. 11
```

```
>osa-cand005b
TTAGAGCAAGATTTGGTCAAACTTTTGAAGCCTTGACCATCAATTACTTCTCAGATTCTTAGTTTAAAGACACAAAATAATGATTTGCTTAAGTCTGTCTTGAAAAGTATTATCATAATATCATAAACTTATTAGGTTTATAAACTTATTCTAACATAGAATTGAAGGTTAAAGTTCTAAAAGTTTGTCCGAATCTTGTC
.....((((((((((.(((((((((..(((.((((((.((((((.((....(((.....((((((............(((((.(((((...((......)).)))))..)))))......((((((.....))))))))))))...))).....)))))))).)))))).)))..))))))))).))))))))))). (-55.5)
.....GCAAGATTTGGTCAAACTTTTGA.........................................................................................................................................................................      1
.......AAGATTTGGTCAAACTTTTGAAGC......................................................................................................................................................................      1
................TCAAACTTTTGAAGCCTTGACCAT.............................................................................................................................................................      1
...................AACTTTTGAAGCCTTGACCATCAA..........................................................................................................................................................      11
```

```
>osa-cand006
TCTGTTTGTACCTGTTGACTGACTCTGGTTCAACAAAACCAATTTTACTTTTGTTGAACTAGAGTGGGTCAACGGGTACCCACAGAGTAATTTGTACCCA
(((((..((((((((((((..(((((((((((((((((..........)))))))))))))))))..))))))))))))..))))).............. (-50.7)
....TTTGTACCTGTTGACTGACTC...........................................................................   1
........TACCTGTTGACTGACTCTGGTTCA....................................................................   8
.........ACCTGTTGACTGACTCTGGTTCAA...................................................................   4
...............TGACTGACTCTGGTTCAACAA................................................................   1
...............TGACTGACTCTGGTTCAACAAAAC.............................................................   2
.....................ACTCTGGTTCAACAAAACCAAT.........................................................   2
.....................................ACCAATTTTACTTTTGTTGAACTA.......................................   1
................................................TTTTGTTGAACTAGAGTGGGTCAA............................   1
.................................................TTTGTTGAACTAGAGTGGGTCAAC...........................   2
........................................................AACTAGAGTGGGTCAACGGGTAC.....................   1
........................................................AACTAGAGTGGGTCAACGGGTACC....................   39
```

```
>osa-cand007
TTAGTTCTCTAGCAGAGCTTGGTTGTACTGTATGATTGTGTCGTGTGTCCCTCATGTGCTTAGGAGCAATGTCTATAGATCAAGAACAATAAGTGATTTAGGCTGAGGAGAATTAAGTGTAATTATGTCTTCCTTAAAATATGGAGAAAGAGAAGTAGCTAGGTACTGGCCTGTTTACTTACAAAGAATATGGAACATTGGCATGCATATATGAGCATGCACAACACCATTTTTATTACTCAAAGATGATGACAACAATTTGATGCGGTATATCTGTTGC
..((((((.....)))))).((.(((((((((((((((((((((...((((((...((((....))))..(((((..(((((...........)))))))))).)))).))...............(((((((.........)))....((((((((..(((((....)))))..))))).....(((.((((....(((((((((........)))))).)))..)))).)))....)))..)))).))))).))))))..)))))))))).))..... (-68)
...................TGGTTGTACTGTATGATTGTG................................................................................................................................................................................................................................................ 66
........................................TCGTGTGTCCCTCATGTGCTT........................................................................................................................................................................................................................... 3
..................................................................................AGAACAATAAGTGATTTAGGC................................................................................................................................................................................. 2
..................................................................................AGAACAATAAGTGATTTAGGCTGA.............................................................................................................................................................................. 2
.......................................................................................................TGAGGAGAATTAAGTGTAATT............................................................................................................................................................ 1
............................................................................................................................ATGTCTTCCTTAAAATATGGA....................................................................................................................................... 1
...........................................................................................................................................................................................ATATGGAACATTGGCATGCAT........................................................................ 1
..........................................................................................................................................................................................................................................................GACAACAATTTGATGCGGTAT......... 1
```

```
>osa-cand008
AACATGTTTTGGGTTTGTCCTAAGTCAAACTTCTTCAAGTTTGACCAAGCTTATAGAAAAACATAGCAACATCTACAGCATCTAATTAGTTTCATTAAGTCCATTGAATATATTTTTATATTATATTTGTTTTGTGTTGGAAATACTGCTGAACTTTTCTATTAAGTTTGTCAAACATGGAGAAGTTTGGCTTAGGACAAACCCATGATGCCTTATAAAG
.....((..((((((((((((((((((((((((((((.(((((((.((((((((((((((...(((((.......((((((..(((...................(((((((.........))))))))))..)))))).......)))))...))))))).)))))))))))))).))))))))))))))))))))))))))))..))........... (-81.8)
.............TTTGTCCTAAGTCAAACTTCT.......................................................................................................................................................................................... 6
...............TGTCCTAAGTCAAACTTCTTC........................................................................................................................................................................................ 2
................GTCCTAAGTCAAACTTCTTCA....................................................................................................................................................................................... 1
....................TAAGTCAAACTTCTTCAAGT.................................................................................................................................................................................... 1
....................TAAGTCAAACTTCTTCAAGTT................................................................................................................................................................................... 5
..................................TCAAGTTTGACCAAGCTTATAGA................................................................................................................................................................... 1
...............................................................................................................................................................................CATGGAGAAGTTTGGCTTAGGACA..................... 1
...................................................................................................................................................................................GAGAAGTTTGGCTTAGGACAA.................... 2
...................................................................................................................................................................................GAGAAGTTTGGCTTAGGACAAACC................. 1
....................................................................................................................................................................................AGAAGTTTGGCTTAGGACAAA................... 15
```

```
>osa-cand009
TCTACTACTGTATGTAGTAGAGATACACGCACAGCAAAAAGACATAAAGGAGAAAGAACACTAGGACCTAGAAAAAAACTTTAGCGCTAAAGTTTTTTTTCTGGGTCCTAGTGCTTTTCTTCTTTATATCTTTATATCTTTTTGTTGTGCCTGTATCTCT
..((((((.....))))))((((((((.((((((((((((((.(((((((((((((..(((((((((((((((((((((((((....))))).)))))))))))))))))))).))))))))))))).........)))))))))))))).)))))))). (-81.1)
.........GTATGTAGTAGAGATACACGCACA...............................................................................................................................  1
.............GTAGTAGAGATACACGCACAGCA............................................................................................................................  1
.............GTAGTAGAGATACACGCACAGCAA...........................................................................................................................  1
..............TAGTAGAGATACACGCACAGCAA...........................................................................................................................  2
..............TAGTAGAGATACACGCACAGCAAA..........................................................................................................................  4
................GTAGAGATACACGCACAGCAA...........................................................................................................................  1
..................AGAGATACACGCACAGCAAAAAGA......................................................................................................................  3
....................AGATACACGCACAGCAAAAAGA......................................................................................................................  3
....................AGATACACGCACAGCAAAAAGAC.....................................................................................................................  1
....................AGATACACGCACAGCAAAAAGACA....................................................................................................................  114
......................ATACACGCACAGCAAAAAGACATA..................................................................................................................  2
..............................ACAGCAAAAAGACATAAAGGAGAA..........................................................................................................  1
..................................CAAAAAGACATAAAGGAGAAAGA.......................................................................................................  1
.....................................AAAGACATAAAGGAGAAAGAACAC...................................................................................................  1
..........................................CATAAAGGAGAAAGAACACTAGGA..............................................................................................  1
............................................TAAAGGAGAAAGAACACTAGGA..............................................................................................  1
............................................TAAAGGAGAAAGAACACTAGGACC............................................................................................  3
.............................................AAAGGAGAAAGAACACTAGGACCT...........................................................................................  1
..............................................AAGGAGAAAGAACACTAGGACC............................................................................................  4
..............................................................................................TTTTTTCTGGGTCCTAGTGCT.............................................  1
.........................................................................................................TCCTAGTGCTTTTCTTCTTTA..................................  5
..................................................................................................................TTTTCTTCTTTATATCTTTATATC......................  1
.....................................................................................................................................ATATCTTTTTGTTGTGCCTGTATC...  1
......................................................................................................................................TATCTTTTTGTTGTGCCTGTATCTC.  1
.......................................................................................................................................ATCTTTTTGTTGTGCCTGTATCTC.  6
........................................................................................................................................TCTTTTTGTTGTGCCTGTATC...  2
........................................................................................................................................TCTTTTTGTTGTGCCTGTATCT..  5
........................................................................................................................................TCTTTTTGTTGTGCCTGTATCTC.  7
........................................................................................................................................TCTTTTTGTTGTGCCTGTATCTCT  81
..........................................................................................................................................TTTTTGTTGTGCCTGTATCTCT  17
............................................................................................................................................TTTGTTGTGCCTGTATCTCT  1
```

```
>osa-cand010
AATCTAGAACCGGATATGACATATCCTAGTACTATGTTTAGATTCGTAGTATTGAGATGTGTCAAATCCGATCCTAGGTTGGTTTTTTATGGGACGGAGG
(((((((...(((((.(((((((((..(((((((((........)))))))))..))))))))).)))))...))))))).((..(....)..))..... (-37.5)
AATCTAGAACCGGATATGACA...............................................................................   2
AATCTAGAACCGGATATGACATAT............................................................................   58
AATCTAGAACCGGATATGACATATC...........................................................................   2
.ATCTAGAACCGGATATGACATATC...........................................................................   2
..TCTAGAACCGGATATGACATATCC..........................................................................   2
....TAGAACCGGATATGACATATC...........................................................................   1
.....AGAACCGGATATGACATATCCTAG.......................................................................   1
.......AACCGGATATGACATATCCTAGT......................................................................   4
...............ATGACATATCCTAGTACTATGTTT.............................................................   1
....................ATATCCTAGTACTATGTTTAGATT........................................................   1
...................................GTTTAGATTCGTAGTATTGAGAT..........................................   1
................................................GTATTGAGATGTGTCAAATCCGA.............................   1
....................................................TGAGATGTGTCAAATCCGATCCT.........................   1
........................................................ATGTGTCAAATCCGATCCTAGGT.....................   5
........................................................ATGTGTCAAATCCGATCCTAGGTT....................   39
........................................................ATGTGTCAAATCCGATCCTAGGTTG...................   4
.........................................................TGTGTCAAATCCGATCCTAGG......................   2
.........................................................TGTGTCAAATCCGATCCTAGGTTG...................   2
..........................................................GTGTCAAATCCGATCCTAGGTTGG..................   62
...........................................................TGTCAAATCCGATCCTAGGTTG...................   1
...........................................................TGTCAAATCCGATCCTAGGTTGG..................   1
.............................................................TCAAATCCGATCCTAGGTTGG..................   8
..............................................................CAAATCCGATCCTAGGTTGGT.................   1
..............................................................CAAATCCGATCCTAGGTTGGTTTT..............   1
.....................................................................GATCCTAGGTTGGTTTTTTATGGG.......   1
.......................................................................TCCTAGGTTGGTTTTTTATGGGA......   1
.......................................................................TCCTAGGTTGGTTTTTTATGGGAC.....   1
........................................................................CCTAGGTTGGTTTTTTATGGG.......   1
.........................................................................CTAGGTTGGTTTTTTATGGGA......   1
.........................................................................CTAGGTTGGTTTTTTATGGGACGG...   1
..........................................................................TAGGTTGGTTTTTTATGGGAC.....   3
...........................................................................AGGTTGGTTTTTTATGGGACGGAG.   1
............................................................................GGTTGGTTTTTTATGGGACGG...   1
............................................................................GGTTGGTTTTTTATGGGACGGAGG   2
.............................................................................GTTGGTTTTTTATGGGACGGA..   1
.............................................................................GTTGGTTTTTTATGGGACGGAG.   1
.............................................................................GTTGGTTTTTTATGGGACGGAGG   1
..............................................................................TTGGTTTTTTATGGGACGGAG.   2
................................................................................GGTTTTTTATGGGACGGAGG   1
```

```
>osa-cand011
TACAAATTTTGTATACACGAATACAAACTTTGCATACACGTATGCAAAGTTTGTATACATGTATGCAAAGTTTGTATACGTATATGCAAATTTTATATAT
...(((.((((((((.(((.((((((((((((((((((.((((((((...)))))))).)))))))))))))))))).))).)))))))).)))...... (-45.2)
.................CGAATACAAACTTTGCATACACGTA..........................................................   1
..................GAATACAAACTTTGCATACACGTA..........................................................   1
...................AATACAAACTTTGCATACACGT...........................................................  18
...................AATACAAACTTTGCATACACGTA..........................................................   2
...................AATACAAACTTTGCATACACGTAT.........................................................   6
...................AATACAAACTTTGCATACACGTATG........................................................   1
....................ATACAAACTTTGCATACACGT...........................................................   2
....................ATACAAACTTTGCATACACGTA..........................................................   1
....................ATACAAACTTTGCATACACGTAT.........................................................   1
....................ATACAAACTTTGCATACACGTATG........................................................   1
......................................................ATACATGTATGCAAAGTTTGTA........................   1
.......................................................TACATGTATGCAAAGTTTGT.........................   1
.......................................................TACATGTATGCAAAGTTTGTA........................  28
```

```
>osa-cand012
GGCGACCAATTACCGCCGTCATCCCTGTCCCTGCAGGCATGGGCGGCCTACAGGGACAGGGATGACGGCAGTAATTGGTCGCCCAGGTAACTAAAGTAGC
(((((((((((((.(((((((((((((((((((.((((.......)))).))))))))))))))))))).))))))))))))).................  (-74.6)
...GACCAATTACCGCCGTCATCCC...........................................................................   2
.....CCAATTACCGCCGTCATCC............................................................................   1
.....CCAATTACCGCCGTCATCCCTG.........................................................................   11
......CAATTACCGCCGTCATCCCTGT........................................................................   3
.......AATTACCGCCGTCATCCCTGTC.......................................................................   3
.........TTACCGCCGTCATCCCTGTCC......................................................................   1
..................TCATCCCTGTCCCTGCAGGCAT............................................................   1
................................................TACAGGGACAGGGATGACGGC...............................   2
....................................................GGGACAGGGATGACGGCAG.............................   1
....................................................GGGACAGGGATGACGGCAGTA...........................   1
.........................................................AGGGATGACGGCAGTAATTGGT.....................   4
.........................................................AGGGATGACGGCAGTAATTGGTC....................   4
..........................................................GGGATGACGGCAGTAATTGGTC....................   12
............................................................GATGACGGCAGTAATTGGTCGC..................   4
..............................................................TGACGGCAGTAATTGGTCGCCC................   2
```

```
>osa-cand013
CTCTGACGTACTGCCCTCTGATGTGGGTCGCTGACATATGGGCCCGCGAGCTAGGGGCCCACATGTCAGCCACTCAAAGTCAGAGGGCAGTAAGTTAGGA
.((((((.((((((((((((((.(((((.((((((((.(((((((.........))))))).)))))))).)))))..)))))))))))))).)))))).  (-68.9)
.........ACTGCCCTCTGATGTGGGTCG......................................................................   1
...................GATGTGGGTCGCTGACATATGGGC.........................................................   1
....................ATGTGGGTCGCTGACATATGGGCC........................................................   15
......................GTGGGTCGCTGACATATGGGCCC.......................................................   1
..............................................................ATGTCAGCCACTCAAAGTCAGAGGG.............   1
...............................................................TGTCAGCCACTCAAAGTCAGAGGGC............   2
```

```
>osa-cand014
ATGGTTGGGGGACGATTTTGTAATCCGATGACAAGATGAGGGACCTTGTATGTACTTTTTCCTAGTAGTAACACCGTATACTCCATTTACTTATATGGGCCAAGTGAATGCCGCGACGCGAGAGACGACAATGAAGAATACTCCATGTATTACGTCCTGGGCCAATATGAACGAATAGTGGGCCGGCCTGGCCTTTACACGCTACATAGATCATGTATCAGAGGATTAAGTTCAATGTAGGTCCCTCATCTTAACATCGAGGTTGGTTTACATCCTCGAACCATAATATGAGATGTAACG
((((((.(((((........((((((((((..((((((((((((((....((.((((..((((.(((((....(((((((...........))))))).....(((((.(((..((((((.....))..........(((((.....))))).))))((((.(((.(((......))).))).))))...))).)))))..)))))...(((.....)))..))))..))))).))....))))))))))))))..))))).)))))).......))))).))))))................ (-96.8)
............CGATTTTGTAATCCGATGACAAG.........................................................................................................................................................................................................................................................................      1
............CGATTTTGTAATCCGATGACAAGA........................................................................................................................................................................................................................................................................      1
.............GATTTTGTAATCCGATGACAAGA........................................................................................................................................................................................................................................................................      1
................TTTGTAATCCGATGACAAGATGAG....................................................................................................................................................................................................................................................................      1
..................TGTAATCCGATGACAAGATGAGGG..................................................................................................................................................................................................................................................................      1
...................GTAATCCGATGACAAGATGAGGG..................................................................................................................................................................................................................................................................      1
...................GTAATCCGATGACAAGATGAGGGA.................................................................................................................................................................................................................................................................      13
....................TAATCCGATGACAAGATGAGG...................................................................................................................................................................................................................................................................      1
....................TAATCCGATGACAAGATGAGGG..................................................................................................................................................................................................................................................................      1
....................TAATCCGATGACAAGATGAGGGA.................................................................................................................................................................................................................................................................      5
....................TAATCCGATGACAAGATGAGGGAC................................................................................................................................................................................................................................................................      2
.....................AATCCGATGACAAGATGAGGGA.................................................................................................................................................................................................................................................................      1
......................ATCCGATGACAAGATGAGGGAC................................................................................................................................................................................................................................................................      2
......................ATCCGATGACAAGATGAGGGACC...............................................................................................................................................................................................................................................................      1
......................ATCCGATGACAAGATGAGGGACCT..............................................................................................................................................................................................................................................................      1
.........................CGATGACAAGATGAGGGACC...............................................................................................................................................................................................................................................................      1
```

```
>osa-cand015a
TGAATGACACTTTCGGCTCAATAGAAGAGAGAATTTCTTTAAAAAGATTTACTTGGGTGAAGATGCAATATAATAAATTATGGTCAAAAGATTTTGAGCTGATACTAAAGTGAAGATGAAATATAATATATACTAGTGATGTACTTGGGTGTTGATAAATTTAGAGTTGCATAATTTCTAATTTTTTTACTCTAACATTATGTTTGAACTTTGAAACACGTGATTCTAGGGGTGTTCTTGTCTTTCCCAACAATTTATCTCTCCAATTGAGTCGAAAGTGTCATATAACAAATTATCAGT
...((((((((((((((((((....((((((...((((....)))).....(((((.((((((..(((((..((((((((((((..((((..((..(((((((..((.((((((((.......((((((.....((((...))))...))))))......((((((.........))))))..)))))))).))..)))).)))..))))))...)).))))))).))...)))))...))))))))))).......))))))..)))))))))))))))))))................ (-73.2)
........................................................................................................................................................................................................................................................AACAATTTATCTCTCCAATTGAGT............................ 1
................................................................................................................................................................................................................................................................ATCTCTCCAATTGAGTCGAAAGT..................... 2
................................................................................................................................................................................................................................................................ATCTCTCCAATTGAGTCGAAAGTG.................... 20
................................................................................................................................................................................................................................................................ATCTCTCCAATTGAGTCGAAAGTGT................... 2
...................................................................................................................................................................................................................................................................TCTCCAATTGAGTCGAAAGTG.................... 5
```

```
>osa-cand015b
CACTTTCGGCTCAATTGTAAAGATAAATTCTTCAAAAAGACGAGAATGCCCCTAGGGTCAAAAGCTTTTGAGCTCAAGCTTGGTACTAGGGGCATTCTCGCCCTTCTTAAGAATTCATCTCTCCAATTGAGTCGAAAGTGTCCTCTACCAAATTACCAAT
((((((((((((((((...((((.(((((((.((.(((.(((((((((((((((..(((..(((((........))))))))..)))))))))))))))..))).))))))))).))))...))))))))))))))))...................... (-72.2)
..CTTTCGGCTCAATTGTAAAGAT................................................................................................................................ 1
.............................CTTCAAAAAGACGAGAATGC............................................................................................... 1
....................................................................................................................ATCTCTCCAATTGAGTCGAAAGT..................... 2
....................................................................................................................ATCTCTCCAATTGAGTCGAAAGTG.................... 20
....................................................................................................................ATCTCTCCAATTGAGTCGAAAGTGT................... 2
.......................................................................................................................TCTCCAATTGAGTCGAAAGTG.................... 5
```

```
>osa-cand016a
CTCCGCCGCATACGCCAACGAGGAGGCCGGGACCGCCGGATCCGGTGACTCCGGCCTCCTCGCCGGCAGATCCGGCCCCTCTGAGGCGAGGGGGAGCCGG
.............(((..(((((((((((((..(((((....)))))..)))))))))))))..)))....(((((((((((......)))))).))))) (-63.6)
...............CAACGAGGAGGCCGGGACC..................................................................   1
................AACGAGGAGGCCGGGACC..................................................................   1
....................AGGAGGCCGGGACCGCCGG.............................................................   2
....................AGGAGGCCGGGACCGCCGGA............................................................   27
....................AGGAGGCCGGGACCGCCGGAT...........................................................   16
....................AGGAGGCCGGGACCGCCGGATC..........................................................   2
.......................................ATCCGGTGACTCCGGCCTCCTCG......................................   1
........................................TCCGGTGACTCCGGCCTCCTCGC.....................................   3
........................................TCCGGTGACTCCGGCCTCCTCGCC....................................   2
........................................TCCGGTGACTCCGGCCTCCTCGCCG...................................   1
.........................................CCGGTGACTCCGGCCTCCTCG......................................   1
.........................................CCGGTGACTCCGGCCTCCTCGC.....................................   2
.........................................CCGGTGACTCCGGCCTCCTCGCC....................................   3
.........................................CCGGTGACTCCGGCCTCCTCGCCG...................................   1
..........................................CGGTGACTCCGGCCTCCTCGC.....................................   1
...........................................GGTGACTCCGGCCTCCTCGCC....................................   4
...............................................ACTCCGGCCTCCTCGCCGGCAGA..............................   1
```

```
>osa-cand016b
ACCCGCCGGATCTCCTAGTGAGGAGGCCGGGACCGCCGGATCCGGTGACCCCGGTCTCCTCGCCGGCGGATCCGGCGCCCAGACGCCCACCTCCTCCGCT
...(((((((((..(..((((((((((((((..(((((....)))))..))))))))))))))..)..))))))))).......................  (-62)
....................AGGAGGCCGGGACCGCCGG.............................................................   2
....................AGGAGGCCGGGACCGCCGGA............................................................   27
....................AGGAGGCCGGGACCGCCGGAT...........................................................   16
....................AGGAGGCCGGGACCGCCGGATC..........................................................   2
........................................TCCGGTGACCCCGGTCTCCTCG......................................   1
.............................................TGACCCCGGTCTCCTCGCCGG..................................   1
...................................................CGGTCTCCTCGCCGGCGGATC............................   1
```

```
>osa-cand016c
GCGCCGTCGCCCACCTCCTCAGCTCGCACCGCAACCTCGCCGCCTCTTGCGCTCGTGTGCGCCGTCGTCGCCGCAGCTCCCTCTCCTCTACTGTGCTACCGCCGCCGCTCCCTCCCAATAAGCCGCCGGATCTCCTAGTGAGGAGGCCGGGACCGCCGGATCCGGTGACCCCGGTCTCCT
(((.((.((............((.((((((((((............)))))...))))).)))).)).)))(((((..............)))))...(((..((.(((...........))).)))))...........(((((((((((..(((((....)))))..))))))))))) (-57.4)
............................................................................................................................................AGGAGGCCGGGACCGCCGG.....................	2
............................................................................................................................................AGGAGGCCGGGACCGCCGGA....................	27
............................................................................................................................................AGGAGGCCGGGACCGCCGGAT...................	16
............................................................................................................................................AGGAGGCCGGGACCGCCGGATC..................	2
```

```
>osa-cand017
CATGGTATTGGATGTGACATTTCCTAGTACAACAAATCTGTCAGGAGCTTATCCAGATTTGTTGTATTAGGGAATGTCTCGTCCAATACTAGGTTGGTTT
..(((((((((((.(((((((((((((((((((((((((....(((.....)))))))))))))))))))))))))))).))))))))))).........  (-55.3)
...........ATGTGACATTTCCTAGTACAA....................................................................   1
...........ATGTGACATTTCCTAGTACAACAA.................................................................   1
............TGTGACATTTCCTAGTACAACA..................................................................   2
............TGTGACATTTCCTAGTACAACAAA................................................................   2
..............TGACATTTCCTAGTACAACAAATC..............................................................   1
...............GACATTTCCTAGTACAACAAATCT.............................................................   14
................ACATTTCCTAGTACAACAAATC..............................................................   1
................ACATTTCCTAGTACAACAAATCT.............................................................   1
................ACATTTCCTAGTACAACAAATCTG............................................................   5
.................CATTTCCTAGTACAACAAATCTGT...........................................................   1
...................................................TCCAGATTTGTTGTATTAGG.............................   1
...................................................TCCAGATTTGTTGTATTAGGGAAT.........................   1
....................................................CCAGATTTGTTGTATTAGGGAA..........................   1
....................................................CCAGATTTGTTGTATTAGGGAATGTCT.....................   1
......................................................AGATTTGTTGTATTAGGGAATGTC......................   5
.......................................................GATTTGTTGTATTAGGGAATGTCT.....................   17
........................................................ATTTGTTGTATTAGGGAATGT.......................   1
........................................................ATTTGTTGTATTAGGGAATGTCTC....................   70
.........................................................TTTGTTGTATTAGGGAATGTCT.....................   2
.........................................................TTTGTTGTATTAGGGAATGTCTC....................   5
.........................................................TTTGTTGTATTAGGGAATGTCTCG...................   5
..........................................................TTGTTGTATTAGGGAATGTCT.....................   4
...........................................................TGTTGTATTAGGGAATGTCTC....................   2
............................................................GTTGTATTAGGGAATGTCTCGTCC................   1
.............................................................TTGTATTAGGGAATGTCTCGTC.................   18
................................................................TATTAGGGAATGTCTCGTCCA...............   2
..................................................................TTAGGGAATGTCTCGTCCAAT.............   1
......................................................................GGAATGTCTCGTCCAATACTA.........   5
.......................................................................GAATGTCTCGTCCAATACTAGGTT.....   1
........................................................................AATGTCTCGTCCAATACTAGGTTG....   1
```

```
>osa-cand018
TTGCATTGTCGTTTTTGTGTTTTTTGGCATTCTGTAACTTGACCGCCCCTTGCATATATATGCAGCCAGCTGAAATTAGCCTTGGCTGGCATCCTCAAGTTAGAGAATGCCAAAAACACA
...............(((((((((.((((((((.((((((((........(((((....)))))((((((..(........)..))))))....)))))))).))))))))))))))))) (-51.1)
...................TTTTTTGGCATTCTGTAACTT................................................................................	1
...................TTTTTTGGCATTCTGTAACTTG...............................................................................	18
...................TTTTTTGGCATTCTGTAACTTGA..............................................................................	1
....................TTTTTGGCATTCTGTAACTTG...............................................................................	1
```

```
>osa-cand019
ACATGAAGTTTGAGAGGCGTTTTGCTCAAGACCGCGCAACGATTCCATATGACACCGCATCGTTGTCCATGCCTATCATGTTCATCCAGAGCCCCTGTTGATCTTCAGATAAGAGAAAAGCTCAAAATTAAAGAAGGCTTGTTTATTGATCATGTCAAGGATAGGTATCGACGGCGATGCGCTGGCTATGGCCATGGTTGTGCGGTTCTTGCGCAATAAC
..................(((((((.((((((((((((((.((.(((((...((.(((((((..(((.((((((((((((.(((....(((((.(((........)))....(((.....))).............)))))......))).)))......))))))))).)))..))))))).))..)))))..)).))))))))).))))).)))).))) (-80.4)
...................TTTTGCTCAAGACCGCGCAA.....................................................................................................................................................................................   1
...................TTTTGCTCAAGACCGCGCAAC....................................................................................................................................................................................   39
....................TTTGCTCAAGACCGCGCAACG...................................................................................................................................................................................   1
.........................TCAAGACCGCGCAACGATTCC..............................................................................................................................................................................   1
```

```
>osa-cand020
ATTCCTCTAACTTCTCAATAACTTGTGTCACTGACATGTGGACCATCTATCCAATGGGTCCGCATGTTAGTGACTCAAAGTCAGAGGATCCCCGATCACGTGATTTCAGGATGGGGATCTCCCACGCGAGAAAGAGAGAA
....((((...(((((......(((.((((((((((((((((((............)))))))))))))))))).))).((..(((.((((((.(((...((....)).))))))))))))..))..)))))..)))).. (-60.3)
...................AACTTGTGTCACTGACATGTGGA..........................................................................................  3
...................AACTTGTGTCACTGACATGTGGAC.........................................................................................  35
...................AACTTGTGTCACTGACATGTGGACC........................................................................................  1
....................ACTTGTGTCACTGACATGTGGACC........................................................................................  11
.....................CTTGTGTCACTGACATGTGGACC........................................................................................  1
......................TTGTGTCACTGACATGTGGACCAT......................................................................................  1
...................................ATGTGGACCATCTATCCAATGGGT.................................................................................  1
.............................................TCTATCCAATGGGTCCGCATGTT........................................................................  1
..............................................................CATGTTAGTGACTCAAAGTCAGA.......................................................  1
.................................................................GTTAGTGACTCAAAGTCAGAGGAT...................................................  1
..............................................................................AGTCAGAGGATCCCCGATCACGTG......................................  1
```

```
>osa-cand021
AGGGGTGGCCGCCGCGGGTATTTCTTGACCCACTTTAGTTCAACTAAAATGAACTAAGTTTCACTTTTAGAGAGTCTAAAGTGAAATTTAGTTCATTTAGTAGGACTAGAATGGGCCAAAGAATACCCATGGGTCAACCC
.(((.(((((..(..(((((((..(((.((((.((((((((.((((((.((((((((((((((((((.((.....)))))))))))))))))))))))))))).)))))))).)))).)))..)))))))..)))))).)))  (-70.1)
..................TATTTCTTGACCCACTTTAGTT....................................................................................................      1
.......................CTTGACCCACTTTAGTTCAACTAA.............................................................................................      1
...............................ACTTTAGTTCAACTAAAATGAA.......................................................................................      1
..............................................AAATGAACTAAGTTTCACTTTTAGA.....................................................................      1
....................................................................................AATTTAGTTCATTTAGTAGGA...................................      1
.....................................................................................ATTTAGTTCATTTAGTAGGACTAG...............................      1
..............................................................................................ATTTAGTAGGACTAGAATGGGCC.......................      1
..............................................................................................ATTTAGTAGGACTAGAATGGGCCA......................      1
...............................................................................................TTTAGTAGGACTAGAATGGGCC.......................      1
...............................................................................................TTTAGTAGGACTAGAATGGGCCA......................      1
...............................................................................................TTTAGTAGGACTAGAATGGGCCAA.....................      1
................................................................................................TTAGTAGGACTAGAATGGGCCA......................      1
................................................................................................TTAGTAGGACTAGAATGGGCCAA.....................      3
................................................................................................TTAGTAGGACTAGAATGGGCCAAA....................      18
```

```
>osa-cand022
TCCCTCTGGTCCAAATTAAGTTCACTTTTGTACATGAATTTGGACATAGGTTATATCCAGATTCATGTACAAAAGTGAATTTATTTTGGCACGAAGGTAG
..(((..(..(((((.((((((((((((((((((((((((((((.(((...))).))))))))))))))))))))))))))))).)))))..)..)))...  (-50)
..............ATTAAGTTCACTTTTGTACATGAA..............................................................   1
...............TTAAGTTCACTTTTGTACATGAAT.............................................................   1
................TAAGTTCACTTTTGTACATGAATT............................................................   3
..................AGTTCACTTTTGTACATGAATTTGG.........................................................   1
...................GTTCACTTTTGTACATGAATTTGG.........................................................   2
....................TTCACTTTTGTACATGAATT............................................................   1
....................TTCACTTTTGTACATGAATTTGG.........................................................   1
....................TTCACTTTTGTACATGAATTTGGA........................................................   30
.......................ACTTTTGTACATGAATTTGGACA......................................................   1
.......................ACTTTTGTACATGAATTTGGACAT.....................................................   4
..............................TACATGAATTTGGACATAGGT.................................................   1
..............................TACATGAATTTGGACATAGGTTATATC...........................................   1
...............................ACATGAATTTGGACATAGGTTATAT............................................   1
......................................................ATCCAGATTCATGTACAAAAGTGA......................   9
```

```
>osa-cand023
TTTTTGCTAGCACAAGACTTTGACCAACAATTACTCTATTAGGATATAATTTTTGTGACACGAAATTGAATTTGTTAGATTCGTATTCAAAAGCACTTAATTATGATTATGCTTCTGTATCACATAAATAATATCCTAATAGAGCAATTGTGGGTCACAG
.(((((......)))))...(((((.((((((.((((((((((((((.((((.(((((.(((((..((((((.....))))))..)))..(((((..((((....)))))))))..)).))))).)))).)))))))))))))).)))))).)))))...  (-50.4)
...TTGCTAGCACAAGACTTTGACCAA.....................................................................................................................................   1
...........ACAAGACTTTGACCAACAATTA...............................................................................................................................   1
...........ACAAGACTTTGACCAACAATTACT.............................................................................................................................   3
.............AAGACTTTGACCAACAATTACTCT...........................................................................................................................   1
................ACTTTGACCAACAATTACTCTATT........................................................................................................................   2
....................TGACCAACAATTACTCTATTAG......................................................................................................................   5
....................TGACCAACAATTACTCTATTAGG.....................................................................................................................   1
....................TGACCAACAATTACTCTATTAGGA....................................................................................................................   27
......................ACCAACAATTACTCTATTAGGAT...................................................................................................................   1
......................ACCAACAATTACTCTATTAGGATA..................................................................................................................   6
.........................AACAATTACTCTATTAGGATA..................................................................................................................   1
.............................ATTACTCTATTAGGATATAATT.............................................................................................................   1
..............................TTACTCTATTAGGATATAATT.............................................................................................................   1
...................................CTATTAGGATATAATTTTTGTGAC.....................................................................................................   1
............................................................................................................................TAAATAATATCCTAATAGAGC...............   1
.....................................................................................................................................TCCTAATAGAGCAATTGTGGG......   1
.....................................................................................................................................TCCTAATAGAGCAATTGTGGGTCA...   1
.......................................................................................................................................CTAATAGAGCAATTGTGGGTCACA.   5
........................................................................................................................................TAATAGAGCAATTGTGGGTCA...   1
.........................................................................................................................................AATAGAGCAATTGTGGGTCACA.   2
..........................................................................................................................................ATAGAGCAATTGTGGGTCACA.   2
```

```
>osa-cand024
CCATTGCTGCCAGCATTTTGATTCTGTGTGTGTGTCTTGGTTGCAAAAACCAAGACATACACACAGAATCAAATAATAACAAAGGTTTCCTAGCCTCAAA
....(((.....))).((((((((((((((((((((((((((.....))))))))))))))))))))))))))).........(((((....))))).... (-48)
..................TGATTCTGTGTGTGTGTCTTGGT...........................................................   1
..................TGATTCTGTGTGTGTGTCTTGGTT..........................................................   6
..................TGATTCTGTGTGTGTGTCTTGGTTGC........................................................   1
...................GATTCTGTGTGTGTGTCTTGG............................................................   5
...................GATTCTGTGTGTGTGTCTTGGT...........................................................  34
...................GATTCTGTGTGTGTGTCTTGGTT..........................................................  15
...................GATTCTGTGTGTGTGTCTTGGTTG.........................................................   2
....................ATTCTGTGTGTGTGTCTTGGT...........................................................   1
.....................TTCTGTGTGTGTGTCTTGGTT..........................................................   1
......................TCTGTGTGTGTGTCTTGGTT..........................................................   1
..............................................AAACCAAGACATACACACAGAATCA.............................   1
...............................................AACCAAGACATACACACAGAA................................   1
...............................................AACCAAGACATACACACAGAATC..............................   1
...............................................AACCAAGACATACACACAGAATCA.............................  11
...............................................AACCAAGACATACACACAGAATCAA............................   1
...............................................AACCAAGACATACACACAGAATCAAA...........................   3
................................................ACCAAGACATACACACAGAA................................   1
................................................ACCAAGACATACACACAGAATC..............................   2
................................................ACCAAGACATACACACAGAATCA.............................  13
.................................................CCAAGACATACACACAGAATCA.............................   4
.....................................................GACATACACACAGAATCA.............................   1
```

```
>osa-cand025
AAAAAAATCAACCTAGTATTGAAGGTAATATTTTCTAGTATAACGAATCTGGACAGGCTGTCCATATTCGTTGTAATAGGAAATGTCACCTTAATACTAGGCTGGTTTTCTTTGGTCGGA
...(((((((.(((((((((((.(((.((((((((((.((((((((((.(((((.....))))).)))))))))).)))))))))).)))))))))))))).))))))).......... (-53.4)
.AAAAAATCAACCTAGTATTGA.................................................................................................. 1
.AAAAAATCAACCTAGTATTGAAGG............................................................................................... 2
..AAAAATCAACCTAGTATTGAAGGT.............................................................................................. 2
......ATCAACCTAGTATTGAAGGTAATA.......................................................................................... 1
.............TAGTATTGAAGGTAATATTTTC..................................................................................... 56
..............AGTATTGAAGGTAATATTTTCT.................................................................................... 1
..........................AATATTTTCTAGTATAACGAATCT...................................................................... 3
............................TATTTTCTAGTATAACGAATCTGG.................................................................... 1
.............................ATTTTCTAGTATAACGAATCTGGA................................................................... 4
................................TTCTAGTATAACGAATCTGGA................................................................... 2
................................TTCTAGTATAACGAATCTGGACA................................................................. 1
..................................CTAGTATAACGAATCTGGACA................................................................. 1
...................................TAGTATAACGAATCTGGACAGG............................................................... 1
...................................TAGTATAACGAATCTGGACAGGCT............................................................. 1
....................................AGTATAACGAATCTGGACAGGCT............................................................. 1
......................................................AGGCTGTCCATATTCGTTGTAATA.......................................... 1
.........................................................CTGTCCATATTCGTTGTAATAGGA....................................... 2
.................................................................ATTCGTTGTAATAGGAAATGTCAC............................... 6
.............................................................................AGGAAATGTCACCTTAATACTAGG................... 1
................................................................................AAATGTCACCTTAATACTAGG................... 96
................................................................................AAATGTCACCTTAATACTAGGCT................. 1
.................................................................................AATGTCACCTTAATACTAGGCTGG............... 3
............................................................................................AATACTAGGCTGGTTTTCTTTGGT.... 2
```

```
>osa-cand026
CTTTGTAGTTGACAACTTTTTCATTAAAATAATTTATGGGTCATAATATTTATATTTATATTTGTATTTAGCCCCCTCTCGTGGGTTTTCACTAATCTGAAAATTAATATTGATTATTTCATGCTTTAATAGTTTTAAATGAATGAATGGCTAAGTACAAAATTATAGATCTCGTCGAGCTCTACATCTTTGCTATAGAGCTTGTCTCCATCGGAGGTTGTTTAAAATGTAGATATGAGAATTTGTAAAAATAAATCTAAGACATTATAAGGAATATTTGGACCCATAAGTTATTTTAAT
..((((.....)))).......((((((((((((((((((..(((((((...((((((((((((((((((...((..((..(((((((......)))))))..))...))....(((((..(((((.....)))))...))))).))))))))))))).(((((((((((((((((((((((....))...)))))))))).((((...))))..................))))).))))))))................)))))))))))))..)))))))))))))))))))) (-76.8)
................TTTTTCATTAAAATAATTTATGGG..............................................................................................................................................................................................................................................................        4
.................TTTTCATTAAAATAATTTATGGGT..............................................................................................................................................................................................................................................................        1
...................TTCATTAAAATAATTTATGGGTCA...........................................................................................................................................................................................................................................................        11
....................TCATTAAAATAATTTATGGGT.............................................................................................................................................................................................................................................................        1
......................ATTAAAATAATTTATGGGTCATA.........................................................................................................................................................................................................................................................        5
```

```
>osa-cand027
TGTGTTAGATTGGCTTGGAGTGGTGACAACTGGTAGCAGAGCAATGTCATCTCCACTTGCCGGTTGTTCCTGGTATGTCGAGGGATACTGGTGCTGACATTGCTCTGATACCAATTGTCGGCACTCTAGGCCAATCATAC
.......((((((((((((((..(((((.(((((.((((((((((((((((((.((.((((((......)))))).)).))))...........)))))))))))))).))))).)))))..))))))))))))))....  (-73.2)
TGTGTTAGATTGGCTTGGAGT.......................................................................................................................     9
...GTTAGATTGGCTTGGAGTGGT.....................................................................................................................     88
...GTTAGATTGGCTTGGAGTGGTGAC..................................................................................................................     2
....TTAGATTGGCTTGGAGTGGT.....................................................................................................................     45
....TTAGATTGGCTTGGAGTGGTGA...................................................................................................................     1
.........TTGGCTTGGAGTGGTGACAAC..............................................................................................................     3
.........TTGGCTTGGAGTGGTGACAACT.............................................................................................................     1
..............TTGGAGTGGTGACAACTGGTA.........................................................................................................     3
...............TGGAGTGGTGACAACTGGTA.........................................................................................................     2
...............TGGAGTGGTGACAACTGGTAG........................................................................................................     5
......................GTGACAACTGGTAGCAGAGCAATG..............................................................................................     1
.......................TGACAACTGGTAGCAGAGCA.................................................................................................     1
.......................TGACAACTGGTAGCAGAGCAA................................................................................................     28
.......................TGACAACTGGTAGCAGAGCAATG..............................................................................................     1
........................GACAACTGGTAGCAGAGCAAT...............................................................................................     3
........................GACAACTGGTAGCAGAGCAATG..............................................................................................     2
........................GACAACTGGTAGCAGAGCAATGTC............................................................................................     4
.........................ACAACTGGTAGCAGAGCAATG..............................................................................................     1
.........................ACAACTGGTAGCAGAGCAATGTCA...........................................................................................     1
..........................CAACTGGTAGCAGAGCAATGT.............................................................................................     3
..........................CAACTGGTAGCAGAGCAATGTC............................................................................................     1
...........................AACTGGTAGCAGAGCAATGTC............................................................................................     18
...........................AACTGGTAGCAGAGCAATGTCA...........................................................................................     1
...........................AACTGGTAGCAGAGCAATGTCATC.........................................................................................     28
............................ACTGGTAGCAGAGCAATGTC............................................................................................     1
............................ACTGGTAGCAGAGCAATGTCA...........................................................................................     1
............................ACTGGTAGCAGAGCAATGTCATC.........................................................................................     1
..............................TGGTAGCAGAGCAATGTCATCTCC......................................................................................     1
.................................TAGCAGAGCAATGTCATCTCC......................................................................................     1
.....................................................................................TACTGGTGCTGACATTGCTCTGAT...............................     1
........................................................................................TGGTGCTGACATTGCTCTGAT...............................     3
............................................................................................GCTGACATTGCTCTGATACCAATT........................     1
..............................................................................................TGACATTGCTCTGATACCAAT.........................     3
..............................................................................................TGACATTGCTCTGATACCAATT........................     3
..............................................................................................TGACATTGCTCTGATACCAATTGT......................     6
.................................................................................................CATTGCTCTGATACCAATTGTCGG...................     5
..................................................................................................ATTGCTCTGATACCAATTGTC.....................     7
..................................................................................................ATTGCTCTGATACCAATTGTCGG...................     1
..................................................................................................ATTGCTCTGATACCAATTGTCGGC..................     7
...................................................................................................TTGCTCTGATACCAATTGTCG....................     20
...................................................................................................TTGCTCTGATACCAATTGTCGG...................     71
...................................................................................................TTGCTCTGATACCAATTGTCGGCA.................     1
....................................................................................................TGCTCTGATACCAATTGTC.....................     3
....................................................................................................TGCTCTGATACCAATTGTCG....................     8
....................................................................................................TGCTCTGATACCAATTGTCGG...................     2326
....................................................................................................TGCTCTGATACCAATTGTCGGC..................     1
.....................................................................................................GCTCTGATACCAATTGTCGG...................     4
.....................................................................................................GCTCTGATACCAATTGTCGGC..................     2
.......................................................................................................TCTGATACCAATTGTCGGCAC................     1
.......................................................................................................TCTGATACCAATTGTCGGCACT...............     1
........................................................................................................CTGATACCAATTGTCGGCACTC..............     1
.........................................................................................................TGATACCAATTGTCGGCACTC..............     4
............................................................................................................TACCAATTGTCGGCACTCTAG...........     1
...............................................................................................................CAATTGTCGGCACTCTAGG..........     1
................................................................................................................AATTGTCGGCACTCTAGGCCAATC....     5
```

```
>osa-cand028
TGGAACCCGTCAGAGGTGTTCGAGTCACAGGTCAAGAATGGAACCCGCCAAAGGTGTTCTATTCTCGACCCGTCACAGGTGACTAGATACCTGTGACGGGCCGTTGCCGGATGGCTCACC
.((..(((((((.(((((((..((((((.((((.((((((((((((......)).)))))))))).))))........)))))).))))))).)))))))))...(((....))).....  (-57)
............................................................................AGGTGACTAGATACCTGTGACGGG....................        58
.............................................................................GGTGACTAGATACCTGTGACGG.....................        1
.............................................................................GGTGACTAGATACCTGTGACGGG....................        1
.............................................................................GGTGACTAGATACCTGTGACGGGC...................        13
..............................................................................GTGACTAGATACCTGTGACGGGC...................        1
..............................................................................GTGACTAGATACCTGTGACGGGCC..................        1
...............................................................................TGACTAGATACCTGTGACGGGC...................        2
...............................................................................TGACTAGATACCTGTGACGGGCC..................        1
................................................................................GACTAGATACCTGTGACGGG....................        2
```

```
>osa-cand029
TAGGACAAAATTTGGTTAAACTTTTGATATTTGGGCCCATCAATTTTATATTAGAATAAGTACAGAGATCTAATATGTTTATGACATTACATTAGTACTTTTCAAGGCAAATCTACACACACCATGTTTTTTTTAAAAAAACTAAGCATTTGAGACACTATATCGGGTCAAAGTTTCAAAAGTTTAATCAAATCTTGTCCTAAACATCAAATATTATAAC
((((((((.((((((((((((((((((.((((.(((((.(((....(((((((((.............)))))))))....)))........((((...(((((((((.................(((((((...)))))))...)).))))))).))))....))))).)))).)))))))))))))))))).)))))))).................. (-56.8)
....................................................................................................................................................................................AGTTTAATCAAATCTTGTCCT...................   10
```

```
>osa-cand030
TGCTTAGGTGACATGCGCCATCGCCATGGTTTGAAATTCGGTAATCTTGGCCGCCATCGGCAAGCACGAATCTCACCAAAATATTAGGTTTTTTTTATCAAATATTTGTAAATTTGGTAAAAATTTGTTCAAATTCAATCAAAATATGTTAAAAATTCAAATAATTTCGGCAAAAAAAACAAAAAATCACGAAATTTTTGGCAACCACCGAAATTTCTGAGCAACTGAAATTGCAAACCCTGGCCATTCATATTCAGCCC
.(((..((((.....))))...((((.((((((((.((((((.......((((....))))..((.((((....(((.........)))..((((((((((((........))))))))))))........................................))))))..............(((.((((((((.((......)))))))))))))...)))))).)).)))))).))))..........)))..  (-55.4)
TGCTTAGGTGACATGCGCCAT.......................................................................................................................................................................................................................................................     1
....................TCGCCATGGTTTGAAATTCGG...................................................................................................................................................................................................................................     23
.........................................TAATCTTGGCCGCCATCGGCA.............................................................................................................................................................................................................     2
.................................................................................TTAGGTTTTTTTTATCAAATA.....................................................................................................................................................................     2
.....................................................................................................TTTGTAAATTTGGTAAAAATT.................................................................................................................................................     1
..............................................................................................................TTGGTAAAAATTTGTTCAAAT........................................................................................................................................     1
```

```
>osa-cand031
AAAACCGGTACCAATACTATATGTACTGGTTTTTTAAAAGAACTTACACCTATTAATATATTACAGGTGCTGGTTCTTTAAACAACCAGTGCCTACCAAACTGAACCGACACGAGCGAGCCGAGCCGGCCGAGCCGGCCG
.....((((..((.........(((((((((.((((((.(((((..(((((.............)))))..))))))))))).))))))))).........)).))))............((.((((((...)))))))) (-44.6)
...ACCGGTACCAATACTATATGTACT.................................................................................................................      1
..................ATATGTACTGGTTTTTTAAAAGAA..................................................................................................      55
...................TATGTACTGGTTTTTTAAAAGAAC.................................................................................................      1
....................ATGTACTGGTTTTTTAAAAGAA..................................................................................................      1
....................ATGTACTGGTTTTTTAAAAGAACT................................................................................................      12
......................GTACTGGTTTTTTAAAAGAACTTA..............................................................................................      2
.............................TTTTTTAAAAGAACTTACACC..........................................................................................      1
..............................TTTTTAAAAGAACTTACACCTATT......................................................................................      1
................................TTTAAAAGAACTTACACCTATTAA....................................................................................      1
...................................AAAAGAACTTACACCTATTAATAT.................................................................................      1
....................................AAAGAACTTACACCTATTAATATAT...............................................................................      1
......................................AGAACTTACACCTATTAATATATT..............................................................................      1
.........................................ACTTACACCTATTAATATATTACA...........................................................................      1
.........................................................ATATTACAGGTGCTGGTTCT...............................................................      1
.....................................................................................CCAGTGCCTACCAAACTGAACCGA...............................      2
......................................................................................CAGTGCCTACCAAACTGAACCGAC..............................      1
................................................................................................CAAACTGAACCGACACGAGCGAGC....................      1
.................................................................................................AAACTGAACCGACACGAGCGAGCC...................      1
...................................................................................................ACTGAACCGACACGAGCGAGCCGA.................      2
......................................................................................................GAACCGACACGAGCGAGCCGAGCC..............      1
........................................................................................................ACCGACACGAGCGAGCCGAGCCGG............      2
```

```
>osa-cand032
AAAATCTGGACTGTTTGGGGGAGCTTCTGACAGCTGCAGTTTCTCTTGTTCTAGGAGAAGCTGCAGCTGTCAGAAGCTCCCCCAAACATGGCCTAAAGCT
.......((..(((((((((((((((((((((((((((((((((((((...)))))))))))))))))))))))))))))))))))))...)).......  (-73.9)
......TGGACTGTTTGGGGGAGCTTC.........................................................................  3
................GGGGGAGCTTCTGACAGCTGCAGT............................................................  1
.....................AGCTTCTGACAGCTGCAGTT...........................................................  1
.....................AGCTTCTGACAGCTGCAGTTTC.........................................................  1
.....................AGCTTCTGACAGCTGCAGTTTCT........................................................  6
.....................AGCTTCTGACAGCTGCAGTTTCTC.......................................................  86
.....................AGCTTCTGACAGCTGCAGTTTCTCT......................................................  1
......................GCTTCTGACAGCTGCAGTTTCT........................................................  3
......................GCTTCTGACAGCTGCAGTTTCTCT......................................................  26
.......................CTTCTGACAGCTGCAGTTTCTC.......................................................  7
........................TTCTGACAGCTGCAGTTTCTC.......................................................  4
........................TTCTGACAGCTGCAGTTTCTCT......................................................  2
........................TTCTGACAGCTGCAGTTTCTCTT.....................................................  1
........................TTCTGACAGCTGCAGTTTCTCTTG....................................................  1
.........................TCTGACAGCTGCAGTTTCTCT......................................................  1
...........................TGACAGCTGCAGTTTCTCTTG....................................................  3
...........................TGACAGCTGCAGTTTCTCTTGTT..................................................  2
..................................................CTAGGAGAAGCTGCAGCTGTC.............................  1
.....................................................GGAGAAGCTGCAGCTGTCAGAAGC.......................  1
......................................................GAGAAGCTGCAGCTGTCAGA..........................  1
.......................................................AGAAGCTGCAGCTGTCAGAA.........................  1
.......................................................AGAAGCTGCAGCTGTCAGAAGC.......................  2
.......................................................AGAAGCTGCAGCTGTCAGAAGCT......................  3
.......................................................AGAAGCTGCAGCTGTCAGAAGCTC.....................  149
........................................................GAAGCTGCAGCTGTCAGAAGCT......................  6
........................................................GAAGCTGCAGCTGTCAGAAGCTC.....................  6
........................................................GAAGCTGCAGCTGTCAGAAGCTCC....................  299
........................................................GAAGCTGCAGCTGTCAGAAGCTCCC...................  1
.........................................................AAGCTGCAGCTGTCAGAA.........................  1
.........................................................AAGCTGCAGCTGTCAGAAGCT......................  3
.........................................................AAGCTGCAGCTGTCAGAAGCTC.....................  5
.........................................................AAGCTGCAGCTGTCAGAAGCTCC....................  1
.........................................................AAGCTGCAGCTGTCAGAAGCTCCC...................  1
..........................................................AGCTGCAGCTGTCAGAAGCTCCCC..................  2
...........................................................GCTGCAGCTGTCAGAAGCTCCCC..................  1
...........................................................GCTGCAGCTGTCAGAAGCTCCCCC.................  2
.............................................................TGCAGCTGTCAGAAGCTCCCCCA................  1
```

```
>osa-cand033
CGAATCGAGCCTAAGACAAGTCGAGCTTGAGCAGCTCGTGAGCCTCGAGCTTTTTTTTTTTTGACAACCTCGAGGCTCGCGTGCTCTTCATATAGCGAAA
((..((((((....((....))..))))))..(((.(((((((((((((.((.((.......)).)).))))))))))))).)))..........))... (-36.6)
..AATCGAGCCTAAGACAAGTCGA............................................................................   1
.......AGCCTAAGACAAGTCGAGCTTGAG.....................................................................   1
............................................................TTGACAACCTCGAGGCTCGCG...................   24
```

```
>osa-cand034
TTAATAAACCTATTTATAGGTCAAATTAGTAAGTAACCCACGAGTCAACCCGCGGGTTACTCACTAATTTTGCCTATAAACGGGTTCGCGGGTTGGCCCG
......((((..((((((((..((((((((.((((((((.((........)).)))))))).))))))))..))))))))..))))..((((....)))) (-45.9)
...........ATTTATAGGTCAAATTAGTAAGT..................................................................   1
...........ATTTATAGGTCAAATTAGTAAGTA.................................................................   1
..............TATAGGTCAAATTAGTAAGTAACC..............................................................   5
................................GTAACCCACGAGTCAACCCGCGGG............................................   1
..............................................AACCCGCGGGTTACTCACTAATTT..............................   1
........................................................TTACTCACTAATTTTGCCTATA......................   2
........................................................TTACTCACTAATTTTGCCTATAA.....................   1
........................................................TTACTCACTAATTTTGCCTATAAA....................   14
............................................................TCACTAATTTTGCCTATAAACGG.................   1
.............................................................CACTAATTTTGCCTATAAACGGGT...............   2
..............................................................ACTAATTTTGCCTATAAACGGGTT..............   2
..................................................................ATTTTGCCTATAAACGGGTTCGCG..........   1
```

```
>osa-cand035a
CTCTATACACAATCTTTTCACTTTGGACTAGACAACTTTATCTTTGTTGCACAATAAACCACCATAACTCTTTACGCTAGTGATGTCTAGCGTTCTTTCGATAAAGGTATAGCAAGCATTTAGACATGAAAATTTACTAGCACGTCAGTAACAATTCGCTCAAAGTCATCGAAGAACTGAAGATGTGGTTTGGGGTGCTTCATTGTGCAACAAAGGTAAAGTTACCTAGTCCAAAGTGAAACTTTGACAATAAAATCCCA
.........(((...((((((((((((((((..((((((((((((((((((((((.((.((((..((((......((((((((((((((((..(((((....)))))....)).......)))))).......))))))))...(((((.....((((...........))))..)))))......))))...)))).)).))))))))))))))))))))))..))))))))))))))))..)))..............  (-87)
.........CAATCTTTTCACTTTGGACTA......................................................................................................................................................................................................................................      1
..........AATCTTTTCACTTTGGACTAGACA..................................................................................................................................................................................................................................      3
...........ATCTTTTCACTTTGGACTAGACAA.................................................................................................................................................................................................................................      1
..............TTTTCACTTTGGACTAGACAACTT..............................................................................................................................................................................................................................      2
...............TTTCACTTTGGACTAGACAACTTT.............................................................................................................................................................................................................................      1
.......................................ATCTTTGTTGCACAATAAACCACC.....................................................................................................................................................................................................      2
....................................................AATAAACCACCATAACTCTTTACG........................................................................................................................................................................................      2
...............................................................................GTGATGTCTAGCGTTCTTTCGATA.............................................................................................................................................................      1
.................................................................................................................................AAATTTACTAGCACGTCAGTA..............................................................................................................      1
..................................................................................................................................AATTTACTAGCACGTCAGTAACAA..........................................................................................................      1
..................................................................................................................................................................AAGTCATCGAAGAACTGAAGATGT..........................................................................      1
.......................................................................................................................................................................ATCGAAGAACTGAAGATGTGGTTT.....................................................................      1
......................................................................................................................................................................................................TTCATTGTGCAACAAAGGTAAAG.......................................      1
.......................................................................................................................................................................................................................GTAAAGTTACCTAGTCCAAAGTGA.....................      1
........................................................................................................................................................................................................................TAAAGTTACCTAGTCCAAAGTGA.....................      2
........................................................................................................................................................................................................................TAAAGTTACCTAGTCCAAAGTGAA....................     14
.........................................................................................................................................................................................................................AAAGTTACCTAGTCCAAAGTG......................      1
.........................................................................................................................................................................................................................AAAGTTACCTAGTCCAAAGTGA.....................     52
.........................................................................................................................................................................................................................AAAGTTACCTAGTCCAAAGTGAAA...................      2
..........................................................................................................................................................................................................................AAGTTACCTAGTCCAAAGTGA.....................      3
..........................................................................................................................................................................................................................AAGTTACCTAGTCCAAAGTGAA....................     27
```

```
>osa-cand035b
TACACAATCTTTTTACTTTGGACTAGGTAACTTTACTTTTGTTGCATAATGGACGACCACCCTAACTCTTTTCGCTAGTGATGTCTAGCGTCTCTTTCAACAATAACTCTTTTCGCTAGTGATGCCTAGCGTCTCATTCGACAAAGGTATGGCATGTATATAGGTATGAAAATTTGCTGGCATGTCGTTAATAATTTGCTCGAAGTCATCGAAGAACTGAAGGCGTGGTTTAGGGTGGTCCATTGTGTAACAAAGGTAAAGTTACCTAGTCCAAAGTGAAAATACTGGACAAAGGGTAGT
(((......((((((((((((((((((((((((((((((((((((((((((((...(((((((((..(....((((((......))))))...((((((..............((((((......)))))).....(((((((((...(((((((((.....((((........)))).)))))))))......)))).))))).............))))))...)..)))))))))))))))))))))))))))))))))))))))))))))))))))))..((......)).))).. (-121.3)
.ACACAATCTTTTTACTTTGGACTA................................................................................................................................................................................................................................................................................... 3
...ACAATCTTTTTACTTTGGACTAGG................................................................................................................................................................................................................................................................................. 2
.......TCTTTTTACTTTGGACTAGGTAAC............................................................................................................................................................................................................................................................................. 1
............TTACTTTGGACTAGGTAACTTTAC........................................................................................................................................................................................................................................................................ 1
.............................ACTTTACTTTTGTTGCATAATGGA....................................................................................................................................................................................................................................................... 1
..................................ACTTTTGTTGCATAATGGACGACC.................................................................................................................................................................................................................................................. 1
....................................TTTTGTTGCATAATGGACGACCAC................................................................................................................................................................................................................................................ 1
...............................................AATGGACGACCACCCTAACTCTTT..................................................................................................................................................................................................................................... 1
................................................................ACTCTTTTCGCTAGTGATGTCTAG.................................................................................................................................................................................................................... 1
.................................................................................................................................CGTCTCATTCGACAAAGGTATGGC................................................................................................................................................... 1
...............................................................................................................................................AAGGTATGGCATGTATATAGGTAT..................................................................................................................................... 1
....................................................................................................................................................................TATGAAAATTTGCTGGCATGTCGT................................................................................................................ 1
...................................................................................................................................................................................................TTGCTCGAAGTCATCGAAGAACT.................................................................................. 1
...............................................................................................................................................................................................................................................................GTAAAGTTACCTAGTCCAAAGTGA..................... 1
................................................................................................................................................................................................................................................................TAAAGTTACCTAGTCCAAAGTGA..................... 2
................................................................................................................................................................................................................................................................TAAAGTTACCTAGTCCAAAGTGAA.................... 14
.................................................................................................................................................................................................................................................................AAAGTTACCTAGTCCAAAGTG...................... 1
.................................................................................................................................................................................................................................................................AAAGTTACCTAGTCCAAAGTGA..................... 52
.................................................................................................................................................................................................................................................................AAAGTTACCTAGTCCAAAGTGAAA................... 2
..................................................................................................................................................................................................................................................................AAGTTACCTAGTCCAAAGTGA..................... 3
..................................................................................................................................................................................................................................................................AAGTTACCTAGTCCAAAGTGAA.................... 27
......................................................................................................................................................................................................................................................................TACCTAGTCCAAAGTGAAAAT................. 2
................................................................................................................................................................................................................................................................................AAAGTGAAAATACTGGACAAAGGG.... 1
```

```
>osa-cand035c
CCAATCTTTTCACTTTAAAATAGGTAACTTTATCTTTGTTGCATAATAGACTATCACAAACCACATCTGTAGCATGTCAACCACGTTGAGCACATTGGCAACGACATGCCGGCAAACTTTCACACTTATGCACATGCCAGATACCTTTTACCGGTGAAGATGTTAGACATGGCTAGTGAAAAGAGTTAGGGTGATTCATCGTAAAAATAAATAAAATAAAGTTACCTAGTCCAAAGTGAAAAGATTGAATACATAATGGT
.((((((((((((((....((((((((((((.((((((........((..(((((...((......((.(((((((......((..(.....)..))...)))))))..)).((((((..((((...((.((((...((((.((((.......)))).))))...)))))).))))...)))))).)))))))...))........))))))))))))))))))....)))))))))))))))).............  (-71.6)
........................................................................................................................................................................................................................TAAAGTTACCTAGTCCAAAGTGA.....................      2
........................................................................................................................................................................................................................TAAAGTTACCTAGTCCAAAGTGAA....................      14
.........................................................................................................................................................................................................................AAAGTTACCTAGTCCAAAGTG......................      1
.........................................................................................................................................................................................................................AAAGTTACCTAGTCCAAAGTGA.....................      52
.........................................................................................................................................................................................................................AAAGTTACCTAGTCCAAAGTGAAA...................      2
..........................................................................................................................................................................................................................AAGTTACCTAGTCCAAAGTGA.....................      3
..........................................................................................................................................................................................................................AAGTTACCTAGTCCAAAGTGAA....................      27
...........................................................................................................................................................................................................................AGTTACCTAGTCCAAAGTGAAAAG.................      2
............................................................................................................................................................................................................................GTTACCTAGTCCAAAGTGAAAAGA................      3
..............................................................................................................................................................................................................................TACCTAGTCCAAAGTGAAAAGA................      2
...............................................................................................................................................................................................................................ACCTAGTCCAAAGTGAAAAGAT...............      1
............................................................................................................................................................................................................................................TGAAAAGATTGAATACATAATGGT      1
```

```
>osa-cand035d
CCTGTACACAATCTTTTCATTTTGGACTAGGTAACTTTACCTTTCTTGCGTAATGGACCACTCTAACTCTTTACGTTAGTGATGTCTAGCGTCTCTTTTGACAAAGGTATAGCAAGCATTTAGGTATGAAAATTTGTTGGCATGTCGTTAACAATTTGCTTGAAATCATCGAAAAACTGAAGATGTGGTTTGGGGTGGTTCATTGTGTAACAAAGGTAAAGTTACCTAGTCCAAAGTGAAAAGATTGGACAAATGGTAGT
((......((((((((((((((((((((((((((((((((((((.((((..(((((((((((((((..(...(((((((......))))))).(((((.......(((((..((((((((((....))))...(((((..(.....)..)))))..))))))..)).)))........)))))...)..)))))))))))))))..)))).))))))))))))))))))))))))))))))))))))).......))....  (-108.2)
.....ACACAATCTTTTCATTTTGGACT........................................................................................................................................................................................................................................       1
.............TTTTCATTTTGGACTAGGTAACTT...............................................................................................................................................................................................................................       1
..............TTTCATTTTGGACTAGGTAACTTT..............................................................................................................................................................................................................................       1
................TCATTTTGGACTAGGTAACTTTAC............................................................................................................................................................................................................................       1
.................CATTTTGGACTAGGTAACTTTACC...........................................................................................................................................................................................................................       1
....................TTTGGACTAGGTAACTTTACCTTT........................................................................................................................................................................................................................       1
.....................TTGGACTAGGTAACTTTACCTTTC.......................................................................................................................................................................................................................       1
............................AGGTAACTTTACCTTTCTTGCGTA................................................................................................................................................................................................................       1
...................................TTTACCTTTCTTGCGTAATGGACC.........................................................................................................................................................................................................       1
.................................................................................ATGTCTAGCGTCTCTTTTGACA.............................................................................................................................................................       1
...........................................................................................TCTCTTTTGACAAAGGTATAGCAA.................................................................................................................................................       1
.....................................................................................................................................TTGTTGGCATGTCGTTAACAATT........................................................................................................       1
.......................................................................................................................................................................................................................GTAAAGTTACCTAGTCCAAAGTGA.....................       1
........................................................................................................................................................................................................................TAAAGTTACCTAGTCCAAAGTGA.....................       2
........................................................................................................................................................................................................................TAAAGTTACCTAGTCCAAAGTGAA....................       14
.........................................................................................................................................................................................................................AAAGTTACCTAGTCCAAAGTG......................       1
.........................................................................................................................................................................................................................AAAGTTACCTAGTCCAAAGTGA.....................       52
.........................................................................................................................................................................................................................AAAGTTACCTAGTCCAAAGTGAAA...................       2
..........................................................................................................................................................................................................................AAGTTACCTAGTCCAAAGTGA.....................       3
..........................................................................................................................................................................................................................AAGTTACCTAGTCCAAAGTGAA....................       27
...........................................................................................................................................................................................................................AGTTACCTAGTCCAAAGTGAAAAG.................       2
............................................................................................................................................................................................................................GTTACCTAGTCCAAAGTGAAAAGA................       3
..............................................................................................................................................................................................................................TACCTAGTCCAAAGTGAAAAGA................       2
...............................................................................................................................................................................................................................ACCTAGTCCAAAGTGAAAAGAT...............       1
```

```
>osa-cand036
GTATTACTCCCTTCATTTCAAATTACTTGTCGTTCTAGCTTTGTCCTTAAACATGTATATCTTTGACCATCAATTTTTAAAAACTCATATGGTTTAACATCATATGATTTTTTTTTAGATTCACTGACAAATACTTTAATAAATACAACTCATATTTTGAACTATACGGATTTCTAAAAATTGATGGTCAAAGATATACATGCTTGACTTAGGACAAAGCTAGAACAAAAAGTATTTGAG
................(((((((.((((...(((((((((((((((((((.((((((((((((((((((((((((((((.(((..(.((((((((((....((((((......((((......................))))......)))))).)))))))))).)..))).)))))))))))))))))))))))))))).)))....))))))))))))))))...))))))))))) (-87.1)
...............TTTCAAATTACTTGTCGTTCTAG..........................................................................................................................................................................................................          1
...............TTTCAAATTACTTGTCGTTCTAGC.........................................................................................................................................................................................................          3
.................TCAAATTACTTGTCGTTCTAG..........................................................................................................................................................................................................          1
...................AAATTACTTGTCGTTCTAGCTTTG.....................................................................................................................................................................................................          17
....................AATTACTTGTCGTTCTAGCTTTG.....................................................................................................................................................................................................          1
..........................................................................................................................................................................TTTCTAAAAATTGATGGTCAAAGA..............................................          1
```

```
>osa-cand037
CAAATTTTACCCTTGGGACACCTTGCAAAGTTTAAATTTGAACAAATTTTGGCTAACTATAAACATTTTGCACAATTTAAAAGTTATCCAAAACTAATTTTTTTTTTTTACAAAAAGTTGTTTAGTTTTTTTTTTATTTCTGTTATTTGTAACAAGTTTTGATTTTTGCAAAATATTTTAAAGTTCAGCCAAATGTGTTCAAATTTTAATTTTGCAAGGTGT
.........((....))(((((((((((((((.(((((((((((...(((((((((((.((((.((((((((.((.((((((.......((((((((...((((((....))))))....))))))))..........(((((....)))))..))))))).)).)))))))))..))))).))))).)))))))..))))))))))).)))))))))))))))) (-66.9)
...................ACCTTGCAAAGTTTAAATTTGAAC...................................................................................................................................................................................   13
...................ACCTTGCAAAGTTTAAATTTGAACA..................................................................................................................................................................................   1
......................TTGCAAAGTTTAAATTTGAACA..................................................................................................................................................................................   1
...................................................................................................................................................................................................TGTTCAAATTTTAATTTTGCAAGG...   1
......................................................................................................................................................................................................TCAAATTTTAATTTTGCAAGGT..   1
......................................................................................................................................................................................................TCAAATTTTAATTTTGCAAGGTGT   12
```

```
>osa-cand038
ATATTTTTTTTCTTGTTAGAACAAAGCTTTGACCAACTATTGCTTTATTACTAATATATCATTTATGTCACACAAAAGCATAATCATAAGAAAGTACTTTTGAATATGAGTCTAATAACATCAACTTTGTGTTGCAAAAAAATATATTCTAATGAAGTAATTGTTGGTTAAACCCTTGTTCTAACAAAACAAAATATGCCT
((((((.(((..((((((((((...((((((((((.((((((((((((..(((((((..(((.((..(((((((..((((.(((.(((.....))).))).)))).((......))......)))))))..)).)))))))))).))))))))))))).))))))))))...))))))))))))))).)))))))....  (-56.5)
...................AACAAAGCTTTGACCAACTAT.........................................................................................................................................................     1
...................AACAAAGCTTTGACCAACTATTGC......................................................................................................................................................     7
...........................................................................................................................................AATTGTTGGTTAAACCCTTGTTCT...................     11
```

```
>osa-cand039
CTCCCTCCAATAATTCTCTCCCCTCTCCTCCTCTTCACCACCACCACCACCACCAGCAGCAGCAGAGAGCACCATCTCCATCCAATAATCCCCATGCTTGCGCACCACTCCCGGCCACATCCCGCGAGGAGGAGGAGGAGGAGGAGGGTGTGCTTGATCCGCGCTCCCGCCTGGTTGGTGGTGGTGGGGTGAGGGGGGAGGGATGGGAGGGGAGGCAC
...(((((........((((((.((((..((((((((((.((((((((((((((((((((.((.((((((((..(((((.(((.....(((((.(((....)))...((((((((........)).)).))))..)).)))))))))))..)))))..)).)))))...).)))).))))))))))))))))))))))..)))).)))))))))))... (-114.9)
..............................CTCTTCACCACCACCACCACCA......................................................................................................................................................................   21
..............................CTCTTCACCACCACCACCACCAC.....................................................................................................................................................................   1
...............................TCTTCACCACCACCACCACCA......................................................................................................................................................................   1
.............................................................................................................................GAGGAGGAGGAGGAGGAGGA.........................................................................   1
.............................................................................................................................GAGGAGGAGGAGGAGGAGGAGGG......................................................................   1
...............................................................................................................................GGAGGAGGAGGAGGAGGA.........................................................................   1
...............................................................................................................................GGAGGAGGAGGAGGAGGAGG.......................................................................   1
...............................................................................................................................GGAGGAGGAGGAGGAGGAGGG......................................................................   1
.................................................................................................................................................................................GTGGTGGTGGGGTGAGGGG......................   1
.................................................................................................................................................................................GTGGTGGTGGGGTGAGGGGG.....................   1
.................................................................................................................................................................................GTGGTGGTGGGGTGAGGGGGG....................   21
........................................................................................................................................................................................TGGGGTGAGGGGGGAGGGATGGG...........   1
```

```
>osa-cand040
CTGTTGGAAATGCAGCAGTAGTAGAACTGTAGAACTAGAACATATATACGTTGTCTCTCGGTGCATGGTTGGTTATAAGTAACAATCTGGAACGGGATGCATAGGGAATTCGAGATGAACGACCATGTCGTGTAGAGGCTCCTGCCGACGCATCACGGCT
((((((......))))))..(((....(((.........)))....)))...((((((((..(((((((((((((....)))).((((.(((................))).))))...)))))))))..)).))))))....((((........)))). (-38.4)
.......................................................................................................................CGACCATGTCGTGTAGAGGCT....................   12
```

```
>osa-cand041
TGTTTGGATTTGATTGACAATGTTGATCCATTAGTCCATTCATTTAATTTGCTCGCCTTTTCTTCCCCTCGTGTGTTCATTTGCTAATTACATTTGTAAAGAGGGGAAAATGAGGTGGCATTACTATTATTTTTTATAAT
((..((((((.(((..((...))..)))....))))))..)).......(((.((((((...((((((((....((......))...((((....)))).))))))))...)))))))))....................  (-38.9)
........TTTGATTGACAATGTTGATCCATT............................................................................................................      1
.................................................TGCTCGCCTTTTCTTCCCCTCG.....................................................................      1
..................................................GCTCGCCTTTTCTTCCCCTCG.....................................................................      1
...................................................CTCGCCTTTTCTTCCCCTCG.....................................................................      1
....................................................TCGCCTTTTCTTCCCCTCGT....................................................................      1
..........................................................TTTCTTCCCCTCGTGTGTTCATT...........................................................      1
..................................................................................................AAGAGGGGAAAATGAGGTGGCATTACT...............      1
...................................................................................................AGAGGGGAAAATGAGGTGGC.....................      1
...................................................................................................AGAGGGGAAAATGAGGTGGCAT...................      1
....................................................................................................GAGGGGAAAATGAGGTGGC.....................      1
....................................................................................................GAGGGGAAAATGAGGTGGCA....................     11
....................................................................................................GAGGGGAAAATGAGGTGGCAT...................      1
```

```
>osa-cand042
ATTCAACTAATCATGTGCCAATGGCTTGTCTTGTTTTGTGTGCCATAAAAAACACTAGGCTCAGTTCGGAATGAGTGTTGGCTTGAATTTTTGATGGCACGCAAAACGAGCCAAGTCATT
...................(((((((((.(((((((((((((((((.(((((...((((((((.((......)).))..))))))..))))).))))))))))))))))).))))))))) (-45)
..................CAATGGCTTGTCTTGTTTTGTGTG..............................................................................        13
..................CAATGGCTTGTCTTGTTTTGTGTGCCA...........................................................................        1
...................AATGGCTTGTCTTGTTTTGTGT...............................................................................        1
...................AATGGCTTGTCTTGTTTTGTGTG..............................................................................        5
...................AATGGCTTGTCTTGTTTTGTGTGC.............................................................................        225
....................ATGGCTTGTCTTGTTTTGTGT...............................................................................        1
....................ATGGCTTGTCTTGTTTTGTGTGC.............................................................................        2
....................ATGGCTTGTCTTGTTTTGTGTGCC............................................................................        2
.....................TGGCTTGTCTTGTTTTGTGTGCC............................................................................        1
.....................TGGCTTGTCTTGTTTTGTGTGCCA...........................................................................        1
.......................GCTTGTCTTGTTTTGTGTGC.............................................................................        1
.......................GCTTGTCTTGTTTTGTGTGCCATAA........................................................................        1
............................TCTTGTTTTGTGTGCCATAAAA......................................................................        1
.............................CTTGTTTTGTGTGCCATAAAAAAC...................................................................        1
....................................TGTGTGCCATAAAAAACACTAGGC............................................................        1
......................................................................................ATTTTTGATGGCACGCAAAACGAG..........        1
.........................................................................................TTTGATGGCACGCAAAACGAGCCA.......        1
................................................................................................GCACGCAAAACGAGCCAAGTCATT        1
..................................................................................................ACGCAAAACGAGCCAAGTCATT        1
```

```
>osa-cand043
AAAGTTGTTTGGCTACTGGTAATCAAGTTAGGAACCATGCAAGTTGTTTATGAGTTGTGCCCTCTGTGCCTAGCGCCATAGATGACTTGCAAGATTTCTAACTTGAGTGCCAGTGTCCAA
........((((..(((((((.((((((((((((...(((((((..((((((.((((..(.....)..)..)))..))))))..)))))))...)))))))))))).)))))))..))))  (-51.4)
.......TTTGGCTACTGGTAATCAAGT............................................................................................        1
........TTGGCTACTGGTAATCAAGTTAGG........................................................................................        2
............CTACTGGTAATCAAGTTAGGA.......................................................................................        3
....................AATCAAGTTAGGAACCATGCAAG.............................................................................        1
....................AATCAAGTTAGGAACCATGCAAGT............................................................................        67
....................AATCAAGTTAGGAACCATGCAAGTT...........................................................................        1
.....................ATCAAGTTAGGAACCATGCAAGTT...........................................................................        3
........................AAGTTAGGAACCATGCAAGTTGTT........................................................................        1
.........................AGTTAGGAACCATGCAAGTTG..........................................................................        1
.........................AGTTAGGAACCATGCAAGTTGTTT.......................................................................        1
...............................................................................AGATGACTTGCAAGATTTCTAACT.................        4
.................................................................................ATGACTTGCAAGATTTCTAACTTG...............        22
.....................................................................................CTTGCAAGATTTCTAACTTGAGTG...........        1
......................................................................................TTGCAAGATTTCTAACTTGAGTG...........        11
......................................................................................TTGCAAGATTTCTAACTTGAGTGC..........        17
.......................................................................................TGCAAGATTTCTAACTTGAGT............        2
.........................................................................................CAAGATTTCTAACTTGAGT............        1
```

```
>osa-cand044
CCTCCGTCCGAAAATGAACCAACTTTTGTATGTGAATATGGACATAGGCTATGTCTATATTCATTTACAAAAGTTGGTTTATTTTTGGACGGAGGAAGTA
((((((((((((((((((((((((((((((..((((((((((((((...))))))))))))))..))))))))))))))))))))))))))))))..... (-67.1)
.....GTCCGAAAATGAACCAACTTTTGT.......................................................................  1
.........GAAAATGAACCAACTTTTGTATGT...................................................................  1
..........AAAATGAACCAACTTTTGTATGTG..................................................................  4
.............ATGAACCAACTTTTGTATGTG..................................................................  1
..............TGAACCAACTTTTGTATGTGAATA..............................................................  1
................AACCAACTTTTGTATGTGAATA..............................................................  1
................AACCAACTTTTGTATGTGAATATG............................................................  11
....................AACTTTTGTATGTGAATATGGA..........................................................  1
....................AACTTTTGTATGTGAATATGGACA........................................................  101
.....................ACTTTTGTATGTGAATATGGACAT.......................................................  2
............................TATGTGAATATGGACATAGGC...................................................  1
....................................................GTCTATATTCATTTACAAAAGTT.........................  1
.....................................................TCTATATTCATTTACAAAAGTTGG.......................  2
.........................................................TATTCATTTACAAAAGTTGGTT.....................  2
.........................................................TATTCATTTACAAAAGTTGGTTTA...................  1
...........................................................TTCATTTACAAAAGTTGGTTTATT.................  1
................................................................TTACAAAAGTTGGTTTATTTT...............  2
....................................................................AAAAGTTGGTTTATTTTTGGA...........  1
.....................................................................AAAGTTGGTTTATTTTTGGAC..........  1
......................................................................AAGTTGGTTTATTTTTGGACG.........  1
.........................................................................TTGGTTTATTTTTGGACGGAG......  3
```

```
>osa-cand045
GTAGATACTCCCTCCGTCCCAATATGTAGCAACCTAGGATGGGTCTAATCCCATCCTAGGTTAACATATTGGGATGGAGGTGTAATAATTTGATATTTGT
.....(((..(((((((((((((((((...(((((((((((((......))))))))))))).))))))))))))))))).)))................ (-55.2)
......ACTCCCTCCGTCCCAATATGTAGC......................................................................  1
............TCCGTCCCAATATGTAGCAACC..................................................................  1
............TCCGTCCCAATATGTAGCAACCTA................................................................  5
..............CGTCCCAATATGTAGCAACCTAGG..............................................................  1
...............GTCCCAATATGTAGCAACCTAGGA.............................................................  1
................TCCCAATATGTAGCAACCTAGGA.............................................................  2
................TCCCAATATGTAGCAACCTAGGAT............................................................  6
.................CCCAATATGTAGCAACCTAGGAT............................................................  1
.................CCCAATATGTAGCAACCTAGGATG...........................................................  3
..................CCAATATGTAGCAACCTAGGA.............................................................  2
..................CCAATATGTAGCAACCTAGGAT............................................................  2
..................CCAATATGTAGCAACCTAGGATGG..........................................................  1
...................CAATATGTAGCAACCTAGGATG...........................................................  1
...................CAATATGTAGCAACCTAGGATGG..........................................................  1
...................CAATATGTAGCAACCTAGGATGGG.........................................................  29
....................AATATGTAGCAACCTAGGATG...........................................................  2
....................AATATGTAGCAACCTAGGATGG..........................................................  1
....................AATATGTAGCAACCTAGGATGGGT........................................................  27
.....................ATATGTAGCAACCTAGGATGGG.........................................................  2
.....................ATATGTAGCAACCTAGGATGGGT........................................................  3
.....................ATATGTAGCAACCTAGGATGGGTC.......................................................  3
......................TATGTAGCAACCTAGGATGGG.........................................................  2
```

```
>osa-cand046
TATACGCCAATAAAAAGAAAATGCTTAACTCGATGAGTTGGAGCGTCCCATTCCTGATACCTCACATGCATATAGCGTAACAACACTGCAACAGTAATCCGCTGATCTTAATATTGGTTATTGCATGTGGAGGTTATGAAAACGATTCATTGAGAGGTAGAGAATTCGTAATTTCAGTAG
..(((................(((((..((((((((((((...(((..((....))..((((((((((((.((((((((.......)))..((((.....))))............)))))))))))).))))).)))....)))))))))))))))))..(((........))).))). (-39.4)
...................AATGCTTAACTCGATGAGTTGG...........................................................................................................................................         1
....................ATGCTTAACTCGATGAGTTGG...........................................................................................................................................         20
```

```
>osa-cand047
TACCCCCTCCGTCCCAAAAAAAAGTCCAATCCTAGCTACGAACCCGGACACATGCGTGTCTAAGTTCATAGCCAGGGTTTGGCTTTTTTTTGGGACAGAC
.......((.((((((((((((((((.((((((.((((.((((..((((((....))))))..)))).)))).)))))).)))))))))))))))).)). (-51.1)
..............CAAAAAAAAGTCCAATCCTAGCT..............................................................  1
................AAAAAAAGTCCAATCCTAGCT..............................................................  1
................AAAAAAAGTCCAATCCTAGCTA.............................................................  1
................AAAAAAAGTCCAATCCTAGCTACG...........................................................  1
.................AAAAAAGTCCAATCCTAGCTACGA..........................................................  5
..................AAAAAGTCCAATCCTAGCTACGAA.........................................................  4
...................AAAAGTCCAATCCTAGCTACGA..........................................................  2
...................AAAAGTCCAATCCTAGCTACGAAC........................................................  8
...................AAAAGTCCAATCCTAGCTACGAACC.......................................................  6
....................AAAGTCCAATCCTAGCTACGAACC.......................................................  32
.....................AAGTCCAATCCTAGCTACGAACC.......................................................  1
.....................AAGTCCAATCCTAGCTACGAACCC......................................................  2
...........................AATCCTAGCTACGAACCCGGACAC...............................................  3
```

```
>osa-cand048
TCTGAAGGTAAGAGTTGTAGGAAACTGAACTCTTTCTTGTCAGTGACAGTTTCAGGAGCTCAGTTTTCTCCAACATCTTATCTTCGGCGCCTTAATCTTT
.((((((((((((((((.((((((((((.(((((..(((((...)))))....))))).)))))))))).)))).)))))))))))))............. (-40.2)
..........................................................CTCAGTTTTCTCCAACATCTT.....................   1
..........................................................CTCAGTTTTCTCCAACATCTTA....................   29
```

```
>osa-cand049
TGCAAAGAAACACAAATCAGCACTTCAGCAGTACATCACATCATCATCCTCCTCAGCCTCATCTTCAGCCTCATCCTCCTCCTCAGCCTCATCTTCATCATCAAGTGCTGCTCAAGTGTTGATTTGTGTTTCTTTGCATTATATGGTTTGGAGTTTGGAC
((((((((((((((((((((((((.((((((((.....................((..........))...............((......))..........)))))))).))))))))))))))))))))))))).......(((..(...)..))) (-49.4)
.......................................................................................................................TGATTTGTGTTTCTTTGCATT....................   45
```

```
>osa-cand050
TGTTCCAAGGTTTGTCCTAAGTCAAACATTTGTATCTTTGACCAACAATTTCTAGAAATTCATATAGTTCAACATCATAAAATTAATGTTTTTAAGATGCACCATGAGAAATGTTTTTATAATATACAATTCACATGTTGTTAAACTACACGAATTTCTAGAAACTGATGATCAAAGATAAAAGTGCTTGATTTAGGACAAAGCTAGAACTACAAGTAAT
.((((..((.(((((((((((((((.(((((.(((((((((.((.((.(((((((((((((...(((((...((((.(((((.......))))).))))((.((((.(((.(((...........))).))).)))).))...)))))...))))))))))))).)).)).))))))))).))))).))))))))))))))).)).)))).......... (-74.6)
........................................................................................................................................................AATTTCTAGAAACTGATGATCAAA............................................ 4
.............................................................................................................................................................................AAAGATAAAAGTGCTTGATTTAGG....................... 1
..............................................................................................................................................................................AAGATAAAAGTGCTTGATTTAGGA...................... 1
................................................................................................................................................................................GATAAAAGTGCTTGATTTAGGACA.................... 16
...................................................................................................................................................................................AAAAGTGCTTGATTTAGGACA.................... 1
```

```
>osa-cand051
ATCAAACCATCACAGAATACGAAGTGCATGGGGAATTTTGTTGGATTGGTCAGGGTAGTCCATGGACGGAACCGACCTAGACACCGAAATTTTCCATGCACTTCGAAATCTGTCATTCTT
...........(((((...((((((((((((..(((((((.((....((((.((...(((....)))....))))))....)).)))))))..))))))))))))...)))))....... (-52)
....................GAAGTGCATGGGGAATTTTGTT..............................................................................         53
........................................................AGTCCATGGACGGAACCGACCTAG........................................         1
.........................................................GTCCATGGACGGAACCGACCTAGACA.....................................         1
.............................................................ATGGACGGAACCGACCTAGACACC...................................         1
....................................................................................CGAAATTTTCCATGCACTTCGA..............         8
.......................................................................................AAATTTTCCATGCACTTCGAAA..........         1
```

```
>osa-cand052a
TAAGCAGCAACAATTTTCATCAACAAGAAGTTGTATATCTTTTCTCCCGGTCAACGACAATCTTGATAAAGACAAGGTTGCTTCTTGATGGTCGATGATGGGTAGAGATGAAGATGATACAACTTCTTGTTGATGGAAACTATTACTTCTTACCTCTTCT
((((.((......((((((((((((((((((((((((((((((((((((.(((.((((..((((....))))(((((.....)))))...)))).))))))).)))...)))))).))))))))))))))))))))))).......)).)))).......  (-59.1)
..AGCAGCAACAATTTTCATCAACAA......................................................................................................................................  2
.......CAACAATTTTCATCAACAAGAAGT.................................................................................................................................  1
...........AATTTTCATCAACAAGAAGTTGTA.............................................................................................................................  1
............................AGTTGTATATCTTTTCTCCCGGTC............................................................................................................  1
....................................ATCTTTTCTCCCGGTCAACGACAA....................................................................................................  1
....................................................AACGACAATCTTGATAAAGACAAG....................................................................................  1
.....................................................ACGACAATCTTGATAAAGACAAGG...................................................................................  1
..............................................................TTGATAAAGACAAGGTTGCTTCTT..........................................................................  1
...................................................................AAAGACAAGGTTGCTTCTTGATGG.....................................................................  2
....................................................................AAGACAAGGTTGCTTCTTGATGGT....................................................................  1
.....................................................................AGACAAGGTTGCTTCTTGATGGTC...................................................................  1
........................................................................CAAGGTTGCTTCTTGATGGTCG..................................................................  1
........................................................................CAAGGTTGCTTCTTGATGGTCGAT................................................................  1
..........................................................................AGGTTGCTTCTTGATGGTCGAT................................................................  1
...........................................................................GGTTGCTTCTTGATGGTCGATGAT.............................................................  1
.............................................................................TTGCTTCTTGATGGTCGATGATG............................................................  1
....................................................................................TTGATGGTCGATGATGGGTAGAGA....................................................  3
.....................................................................................TGATGGTCGATGATGGGTAGA......................................................  1
.....................................................................................TGATGGTCGATGATGGGTAGAGAT...................................................  4
........................................................................................TGGTCGATGATGGGTAGAGATGAA................................................  1
.............................................................................................GATGATGGGTAGAGATGAAGATG............................................  1
.............................................................................................GATGATGGGTAGAGATGAAGATGA...........................................  1
..............................................................................................ATGATGGGTAGAGATGAAGATGAT..........................................  2
...............................................................................................TGATGGGTAGAGATGAAGATGATA.........................................  1
..................................................................................................TGGGTAGAGATGAAGATGATACA.......................................  1
...................................................................................................GGGTAGAGATGAAGATGATACAAC.....................................  1
.....................................................................................................GTAGAGATGAAGATGATACAACT....................................  1
...........................................................................................................ATGAAGATGATACAACTTCTTGTT.............................  1
.............................................................................................................GAAGATGATACAACTTCTTGTTGA...........................  1
..............................................................................................................AAGATGATACAACTTCTTGTTGAT..........................  2
................................................................................................................GATGATACAACTTCTTGTTGATGG........................  1
.................................................................................................................ATGATACAACTTCTTGTTGATGG........................  1
.................................................................................................................ATGATACAACTTCTTGTTGATGGA.......................  2
..................................................................................................................TGATACAACTTCTTGTTGATGGAA......................  1
...................................................................................................................GATACAACTTCTTGTTGATGGAAA.....................  4
....................................................................................................................ATACAACTTCTTGTTGATGGAAAC....................  1
.....................................................................................................................TACAACTTCTTGTTGATGGAAA.....................  1
.....................................................................................................................TACAACTTCTTGTTGATGGAAACT...................  140
......................................................................................................................ACAACTTCTTGTTGATGGAAACT...................  1
..........................................................................................................................CTTCTTGTTGATGGAAACTATTAC..............  1
```

```
>osa-cand052b
AACAACAACAATTTTCATCAGCAAGAAGTTGTATATATCTTCTCTACCCATATATGACCATCTTAATAAAGGCAAGGTTGTTCCTTATTGGTTGAATATGGATGGAGAAGAAGATTATACAACTTCTTGTTGATGGAAACTATTGCTTCTTCTCTCTTCT
.....(((...((((((((((((((((((((((((..(((((((((.((((((..(((((.(((....)))..((((.....))))..)))))..)))))).)))))))))....))))))))))))))))))))))))...)))............... (-61.3)
...........TTTTCATCAGCAAGAAGTTGT................................................................................................................................ 2
...........TTTTCATCAGCAAGAAGTTGTA............................................................................................................................... 1
...........TTTTCATCAGCAAGAAGTTGTATA............................................................................................................................. 7
..........................................................CATCTTAATAAAGGCAAGGTTGTT.............................................................................. 1
........................................................................................TGGTTGAATATGGATGGAGAAGA................................................. 1
...........................................................................................TTGAATATGGATGGAGAAGAAGAT............................................. 1
.............................................................................................GAATATGGATGGAGAAGAAGATT............................................ 1
..............................................................................................................AAGATTATACAACTTCTTGTTGAT.......................... 1
....................................................................................................................ATACAACTTCTTGTTGATGGAAAC.................... 1
.....................................................................................................................TACAACTTCTTGTTGATGGAAA..................... 1
.....................................................................................................................TACAACTTCTTGTTGATGGAAACT................... 140
......................................................................................................................ACAACTTCTTGTTGATGGAAACT................... 1
```

```
>osa-cand053
AGGAGGGAGATGGAAGGGAAGCGGCGCCGGTGGCGGGGGAAGCCGCCAGCTGGGGGAGGGGGAGAGGGGGCGGCGCCGCCGGAGGGGAGGGGGAGGGGAAGCGGCGGCCCTCTAGCCTCCCTCTCGCGGCAGATCCGCCATCGCGCCGCTGACGCCGCCTCTCCCCCCTCCCCCCAGCTGGCCGCCTCCCCCGCCACCGGCGTCGCTTCCCCTCCCCCTCCCCTCCGGCGGCGCTGCG
.(((((.((.((((.((((((((((((((((((((((((.((.(((((((((((((((((((.((((((.(((((..(((((((((((((.((((((..........))))))..)))))).))).)))).....(((....))).......)))))))))))))))))))))))))))))).)).))))))))))))))))))))))))).))))..)))))))).(((....))). (-200.6)
.................GAAGCGGCGCCGGTGGCGGG......................................................................................................................................................................................................... 1
.................GAAGCGGCGCCGGTGGCGGGG........................................................................................................................................................................................................ 39
..................AAGCGGCGCCGGTGGCGGGG........................................................................................................................................................................................................ 2
..................AAGCGGCGCCGGTGGCGGGGG....................................................................................................................................................................................................... 121
...................AGCGGCGCCGGTGGCGGGGG....................................................................................................................................................................................................... 1
...................AGCGGCGCCGGTGGCGGGGGA...................................................................................................................................................................................................... 14
........................................................................GCGCCGCCGGAGGGGAGGGGG................................................................................................................................................. 1
...........................................................................CCGCCGGAGGGGAGGGGGAGG.............................................................................................................................................. 2
...........................................................................CCGCCGGAGGGGAGGGGGAGGG............................................................................................................................................. 1
..............................................................................CCGGAGGGGAGGGGGAGGGGA........................................................................................................................................... 1
```

```
>osa-cand054
AGGAAAAAATTCATTTTATGCCACTCTATTCGCAAGTCTTTTGGAATTTGCCAAGGAGCAAATTCTGAAAGTCGCAAGAACTGGGTGGCAGGTTCTGAAAGGCTTGTGAATCGAGTGGCGTATAATGAAATTTCTAAGTG
.(((((...((((((.((((((((((.((((((((((((((..(((..(((((((((.....))))...((((....).)))...)))))..)))..))))))))))))))).)))))))))).)))))).))))).....  (-66)
...............TTATGCCACTCTATTCGCAAG........................................................................................................      10
...............TTATGCCACTCTATTCGCAAGT.......................................................................................................      1
...............TTATGCCACTCTATTCGCAAGTCT.....................................................................................................      3
................TATGCCACTCTATTCGCAAGTCTT....................................................................................................      1
..................TGCCACTCTATTCGCAAGTCTTT...................................................................................................      1
..................TGCCACTCTATTCGCAAGTCTTTT..................................................................................................      3
...................GCCACTCTATTCGCAAGTCTTT...................................................................................................      1
....................CCACTCTATTCGCAAGTCTTT...................................................................................................      1
....................CCACTCTATTCGCAAGTCTTTT..................................................................................................      1
....................CCACTCTATTCGCAAGTCTTTTGG................................................................................................      5
....................CCACTCTATTCGCAAGTCTTTTGGA...............................................................................................      2
......................ACTCTATTCGCAAGTCTTTTG.................................................................................................      1
......................ACTCTATTCGCAAGTCTTTTGG................................................................................................      5
......................ACTCTATTCGCAAGTCTTTTGGA...............................................................................................      2
......................ACTCTATTCGCAAGTCTTTTGGAA..............................................................................................      12
...........................ATTCGCAAGTCTTTTGGAAT.............................................................................................      2
...........................ATTCGCAAGTCTTTTGGAATTTGC.........................................................................................      3
.......................................................................................GCAGGTTCTGAAAGGCTTGTGAAT.............................      1
.........................................................................................AGGTTCTGAAAGGCTTGTGAATCG...........................      2
...............................................................................................TGAAAGGCTTGTGAATCGAGTG.......................      2
...............................................................................................TGAAAGGCTTGTGAATCGAGTGG......................      1
...............................................................................................TGAAAGGCTTGTGAATCGAGTGGC.....................      6
................................................................................................GAAAGGCTTGTGAATCGAGTG.......................      1
................................................................................................GAAAGGCTTGTGAATCGAGTGG......................      1
................................................................................................GAAAGGCTTGTGAATCGAGTGGC.....................      9
................................................................................................GAAAGGCTTGTGAATCGAGTGGCG....................      167
.................................................................................................AAAGGCTTGTGAATCGAGTGG......................      1
.................................................................................................AAAGGCTTGTGAATCGAGTGGC.....................      1
..................................................................................................AAGGCTTGTGAATCGAGTGGC.....................      2
..................................................................................................AAGGCTTGTGAATCGAGTGGCG....................      2
..................................................................................................AAGGCTTGTGAATCGAGTGGCGT...................      4
..................................................................................................AAGGCTTGTGAATCGAGTGGCGTA..................      12
...................................................................................................AGGCTTGTGAATCGAGTGGCG....................      1
...................................................................................................AGGCTTGTGAATCGAGTGGCGT...................      1
......................................................................................................CTTGTGAATCGAGTGGCG....................      1
......................................................................................................CTTGTGAATCGAGTGGCGTAT.................      3
..........................................................................................................TGAATCGAGTGGCGTATAATGAAA..........      1
```

```
>osa-cand055
GTGGACTAGTTTTAAGGAGAAATGTCATTATATGCCACTCAATCCGCATGTTGTGAGAAATTGCCACTTAATTCGTGCACCTTTCAGAATTTGCTGGCGGGGCTGCGAGTTCGTGGCAAATTCTGAAAGGCAGACGAATTGAGTGGCATATAATGAAATTTCTCAGTTTTAGGGGGTGAT
....(((..((..((.(((((((.(((((((((((((((((((.((..(((....((((.(((((((..(((((((((.(((..(((......)))..))))).))))))).))))))).)))).....)))..)).))))))))))))))))))).)))))))..))..))..)))...  (-75.4)
....................................ACTCAATCCGCATGTTGTGAGA..........................................................................................................................  1
............................................................TTGCCACTTAATTCGTGCACCTTT................................................................................................  1
...................................................................................................................................AGACGAATTGAGTGGCATATAATG.........................  1
...................................................................................................................................AGACGAATTGAGTGGCATATAATGA........................  1
........................................................................................................................................AATTGAGTGGCATATAATGAAATT....................  30
```

```
>osa-cand056
AGACTGGCATGCTTCTTGTTAAATGGAGAGAACGAAAGAGAGATAGGGTCCTCTGCAATACTCACCTGAGCCTATGGCAGCTCCAACTCTCAGATTCACCTCAGTTCTGACTAGAGCGCAACCAAACGGTCCAGCTCCACAAAAAAATGGAGTGGAGTAGGCCAGATCGCTCCAAAAAATTGTACTAGCTGCGCTCCCAACTCCAACTCCAGAGGACCCTATCTCTCTTTCGTTCTCTCCCTTTAACAAGAAGCATGCCG
.....((((((((((((((((((.(((((((((((((((((((((((((((((((.....(((....)))....(((.((.........(((((...........)))))...((((((........((((..(((((((...........))))))).)))).....(((.(((....)))....))).))))))....)))))....))))))))))))))))))))))))))))))).)))))))))))))))))). (-130.8)
....TGGCATGCTTCTTGTTAAATG...........................................................................................................................................................................................................................................  1
.......CATGCTTCTTGTTAAATGGAG........................................................................................................................................................................................................................................  2
........ATGCTTCTTGTTAAATGGAGA.......................................................................................................................................................................................................................................  1
.........TGCTTCTTGTTAAATGGAGAGA.....................................................................................................................................................................................................................................  1
...........CTTCTTGTTAAATGGAGAGAA....................................................................................................................................................................................................................................  1
............TTCTTGTTAAATGGAGAGAAC...................................................................................................................................................................................................................................  1
...............TTGTTAAATGGAGAGAACGAA................................................................................................................................................................................................................................  4
................TGTTAAATGGAGAGAACGAAAGAG............................................................................................................................................................................................................................  4
................TGTTAAATGGAGAGAACGAAAGAGA...........................................................................................................................................................................................................................  1
.................GTTAAATGGAGAGAACGAAAG..............................................................................................................................................................................................................................  1
.................GTTAAATGGAGAGAACGAAAGAGA...........................................................................................................................................................................................................................  3
..................TTAAATGGAGAGAACGAAAG..............................................................................................................................................................................................................................  2
..................TTAAATGGAGAGAACGAAAGA.............................................................................................................................................................................................................................  21
..................TTAAATGGAGAGAACGAAAGAG............................................................................................................................................................................................................................  1
..................TTAAATGGAGAGAACGAAAGAGAG..........................................................................................................................................................................................................................  1
...................TAAATGGAGAGAACGAAAGA.............................................................................................................................................................................................................................  1
...................TAAATGGAGAGAACGAAAGAG............................................................................................................................................................................................................................  234
...................TAAATGGAGAGAACGAAAGAGA...........................................................................................................................................................................................................................  1
....................AAATGGAGAGAACGAAAGAGA...........................................................................................................................................................................................................................  2
....................AAATGGAGAGAACGAAAGAGAGA.........................................................................................................................................................................................................................  1
.....................AATGGAGAGAACGAAAGAGAG..........................................................................................................................................................................................................................  2
.....................AATGGAGAGAACGAAAGAGAGA.........................................................................................................................................................................................................................  2
..........................AGAGAACGAAAGAGAGATAGG.....................................................................................................................................................................................................................  3
..............................AACGAAAGAGAGATAGGGTCC.................................................................................................................................................................................................................  3
...............................ACGAAAGAGAGATAGGGTCCT................................................................................................................................................................................................................  1
........................................AGATAGGGTCCTCTGCAA..........................................................................................................................................................................................................  1
........................................AGATAGGGTCCTCTGCAATACTCA....................................................................................................................................................................................................  1
..................................................................................................................................................ATGGAGTGGAGTAGGCCAGAT.............................................................................................  1
....................................................................................................................................................................................................................AGGACCCTATCTCTCTTTCGTTCT........................  1
.....................................................................................................................................................................................................................GGACCCTATCTCTCTTTCGTTCTC.......................  1
.................................................................................................................................................................................................................................TCTTTCGTTCTCTCCCTTTAA..............  2
....................................................................................................................................................................................................................................TTCGTTCTCTCCCTTTAACAA...........  1
.....................................................................................................................................................................................................................................TCGTTCTCTCCCTTTAACAAG..........  3
........................................................................................................................................................................................................................................TTCTCTCCCTTTAACAAGAAGC......  1
```

```
>osa-cand057
AAAACGGAGGGAGTAATTGATAGTGAGTGATGATGTGACACACTTATATGTTGAGTTTTAAAAGTTAGTGGAGATGTAGGATGTCGCAAAATTGTCAACGATGTAACTTGGTTTGACGATGGAAAGGTATATTCCATATTGTTACATTATCATTCAACTGACTCATGTGTATTTTGAACCCGACTACTGACACAAAGCAAGATTTTTATAAGTAGAGAAA
....(((...(((((..((((((((((((((((((((((((.(((((.....)))))..........((((((.((((.....((.....(((((((((((......)))..))))))))).....))))))))))))..)))))))))))))))).))))..)))....)))))).....))).(((((.....(((......))).....))))).....  (-47)
....................TAGTGAGTGATGATGTGACACACT................................................................................................................................................................................   58
.....................AGTGAGTGATGATGTGACACACT................................................................................................................................................................................   4
.....................AGTGAGTGATGATGTGACACACTT...............................................................................................................................................................................   2
.......................TGAGTGATGATGTGACACACT................................................................................................................................................................................   2
..........................................................TAAAAGTTAGTGGAGATGTAGGAT..........................................................................................................................................   1
```

```
>osa-cand058
AACATGCTACTCCCTCTGTCCAAATATGTAGCAACCTAGAATGAGATTAGACCAATCCTAGGACTACGAATCTGAAAATAGAGAATCTAGAAATACTCTATGTACAGATTTGTAGTTCTAGGATGGATCTAATCCCATTCTAGGTTGTTACATATTGGGACGGAGGGAGTACGTTATTCC
...(((.((((((((((((((.(((((((((((((((((((((.((((((((((.(((((((((((((((((((...((((((.((......)).))))))...)))))))))))))))))))))).))))))).))))))))))))))))))))).)))))))))))))))))...... (-110.6)
.....................AAATATGTAGCAACCTAGAATGAG.......................................................................................................................................        1
................................AACCTAGAATGAGATTAGACCAAT............................................................................................................................        1
......................................GAATGAGATTAGACCAATCCTAGG......................................................................................................................        1
.............................................ATTAGACCAATCCTAGGACTACGA...............................................................................................................        1
...............................................................................................CTCTATGTACAGATTTGTAGTTCT.............................................................        1
....................................................................................................TGTACAGATTTGTAGTTCTAGGAT........................................................        2
.........................................................................................................AGATTTGTAGTTCTAGGATGGATC...................................................        1
..........................................................................................................GATTTGTAGTTCTAGGATGGATCT..................................................        1
...........................................................................................................ATTTGTAGTTCTAGGATGGATCTA.................................................        1
............................................................................................................TTTGTAGTTCTAGGATGGATC...................................................        1
...............................................................................................................................TCTAATCCCATTCTAGGTTGTTACA............................        1
...................................................................................................................................ATCCCATTCTAGGTTGTTACATAT.........................        1
......................................................................................................................................CCATTCTAGGTTGTTACATATT........................        1
.......................................................................................................................................CATTCTAGGTTGTTACATATTGGG.....................        10
........................................................................................................................................ATTCTAGGTTGTTACATATTGGG.....................        2
........................................................................................................................................ATTCTAGGTTGTTACATATTGGGA....................        20
.........................................................................................................................................TTCTAGGTTGTTACATATTGGGAC...................        18
..........................................................................................................................................TCTAGGTTGTTACATATTGGG.....................        3
..........................................................................................................................................TCTAGGTTGTTACATATTGGGA....................        1
..........................................................................................................................................TCTAGGTTGTTACATATTGGGAC...................        2
..........................................................................................................................................TCTAGGTTGTTACATATTGGGACG..................        18
...........................................................................................................................................CTAGGTTGTTACATATTGGGA....................        1
...........................................................................................................................................CTAGGTTGTTACATATTGGGACGG.................        4
............................................................................................................................................TAGGTTGTTACATATTGGGAC...................        3
............................................................................................................................................TAGGTTGTTACATATTGGGACGGA................        1
.............................................................................................................................................AGGTTGTTACATATTGGGACGGAG...............        1
..............................................................................................................................................GGTTGTTACATATTGGGACGGAGG..............        3
.................................................................................................................................................TGTTACATATTGGGACGG.................        1
.................................................................................................................................................TGTTACATATTGGGACGGAGGGA............        1
```

```
>osa-cand059
CAGCTGACATGGCATGCCATGTGTAGCCACATTGTAAGGGAGTCCTTGTGCACATGGTCAATCTCGCAGAAAGCCCACTGCAATCCAAATCTTCTTCACGATTGTTCCTATTCACCAAATCACCCGTCTTCTCGAATGACCGCCCACCATTTCCCTCTGTTGCTGTTGCTAACATCCATGGCCCAGGGACCCACTTGCAGCGTGGCTACATGTGGCATGC
...........((((((((..(((((((((..((((((((.(((((((.((.((((((((.((..((((........))))......((((........))))..............................)).)))))................((((((....)).))))...))))).))))))))).))))))..)))))))))..))))))))  (-80.2)
.............ATGCCATGTGTAGCCACATTGTAA.......................................................................................................................................................................................   1
..................ATGTGTAGCCACATTGTAAGGG....................................................................................................................................................................................   1
...................TGTGTAGCCACATTGTAAGG.....................................................................................................................................................................................   1
...................TGTGTAGCCACATTGTAAGGG....................................................................................................................................................................................   204
....................GTGTAGCCACATTGTAAGGGA...................................................................................................................................................................................   5
....................GTGTAGCCACATTGTAAGGGAGTC................................................................................................................................................................................   1
.....................TGTAGCCACATTGTAAGGG....................................................................................................................................................................................   1
.....................TGTAGCCACATTGTAAGGGAG..................................................................................................................................................................................   1
......................GTAGCCACATTGTAAGGGAGTCCT..............................................................................................................................................................................   1
.......................TAGCCACATTGTAAGGGAGTCCTT.............................................................................................................................................................................   2
................................................................................................................................................................................................ACTTGCAGCGTGGCTACATGTGGC....   1
.................................................................................................................................................................................................CTTGCAGCGTGGCTACATGTG......   14
..................................................................................................................................................................................................TTGCAGCGTGGCTACATGTGG.....   5
```

```
>osa-cand060
AAGCGTATTTTTAAGATTATAGGGTTGCCACCTTAATGGGAAAGTAAAGCCATCTCTCATGTAAGTAAAATAGAACTTCTCAATTTGGTGCACAATAAATATATTCCCTGATATACAAGAGAGAGGTGGAGAGGAGAGTGACTTGATTATGGGTTCCAAATGTTAATTAGGAGATGAGTAACACACTAGAGCTAACAATGCTAAGCTGCGAGACTGCTTTATAGCTTGTTTGGTGCGAATTCACACTGTAGATGGCCTTACTTTCCCATTAAGGTGACTACCTTATAATCTATAAACAAT
.............(((((((((((...((((((((((((((((((((.(((((((..(((((.((((.......(((((((...(((.....)))....((((((....)))))).....)))))))..((.....((((...((.((((...((((.(((....))).))))....))))))))))....)).....))))..((.((.((((.(((....)))..)))).))))......))).)).))))))).))))))))))))))))))))...))))))))))).........  (-91.5)
...............ATTATAGGGTTGCCACCTTAATGGG....................................................................................................................................................................................................................................................................          1
................TTATAGGGTTGCCACCTTAATGGG....................................................................................................................................................................................................................................................................          2
.................TATAGGGTTGCCACCTTAATGGGA...................................................................................................................................................................................................................................................................          1
..................ATAGGGTTGCCACCTTAATGG.....................................................................................................................................................................................................................................................................          2
...................TAGGGTTGCCACCTTAATGGG....................................................................................................................................................................................................................................................................          44
....................AGGGTTGCCACCTTAATGGGA...................................................................................................................................................................................................................................................................          3
..........................TGCCACCTTAATGGGAAAGTA.............................................................................................................................................................................................................................................................          2
.....................................................................................................................................................................................................................................................................TTTCCCATTAAGGTGACTACC..................          1
```

```
>osa-cand061
GCGCGTGAGCAGCAGCTGCTGCTCCCTTTCCTTGCAACGACATTGCCCTGCCAAAGATGCTTCATACACCAACGTCATGGTAAGGAAAGGTAGCAGTAGCTGCTGCTCTCGCGCGATGAG
(((((.(((((((((((((((((.(((((((((((...(((.(((....((.......))..........))).)))...))))))))))).)))))))))))))))))).)))))...... (-69.6)
................TGCTGCTCCCTTTCCTTGCAACG.................................................................................. 1
............................................................................ATGGTAAGGAAAGGTAGCAGTAGC.................... 29
...............................................................................GTAAGGAAAGGTAGCAGTAGCTG.................. 1
...............................................................................GTAAGGAAAGGTAGCAGTAGCTGC................. 2
................................................................................TAAGGAAAGGTAGCAGTAGCT................... 1
..........................................................................................TAGCAGTAGCTGCTGCTCTCGCGC...... 2
...........................................................................................AGCAGTAGCTGCTGCTCTCGC........ 1
```

Highlighted in red: **ATGGTAAGGAAAGGTAGCAGTAGC**

```
>osa-cand062
CCGAAATGCCTATTTCTGATGTAGGCATGTAAACTTGGTGGACTGAATGATCCTGATGCCTCACAATCCTATACCCGCCTCCGGTATAAACAAATGAAAAGCAATATTCCCCTTCAAAGGGTTTTATCAAGGACAAGCAAATTTGCTTTACTCGAGGAGTATGTAGGTCGCCCACCCAGTTGTGCATGCTTTCTGTGAAT
..(((((....)))))......((((((((((((.(((((..((..((.((((((((..(((......((((((.......))))))......))).............((((....))))...)))).))))))..))...((((..(((((...))))).)))).....))))).)))).)))))))))......... (-48)
...............CTGATGTAGGCATGTAAACTTGGT................................................................................................................................................................. 1
.................GATGTAGGCATGTAAACTTGGTGG............................................................................................................................................................... 1
...................TGTAGGCATGTAAACTTGGT................................................................................................................................................................. 1
...................TGTAGGCATGTAAACTTGGTG................................................................................................................................................................ 47
..............................AAACTTGGTGGACTGAATGATCCT.................................................................................................................................................. 5
..........................................CTGAATGATCCTGATGCCTCACAA...................................................................................................................................... 1
.................................................................................CGGTATAAACAAATGAAAAGCAAT............................................................................................... 1
.............................................................................................ATGAAAAGCAATATTCCCCTT...................................................................................... 1
.......................................................................................................ATATTCCCCTTCAAAGGGTTT............................................................................ 1
................................................................................................................TTCAAAGGGTTTTATCAAGGA................................................................... 1
.........................................................................................................................................CAAATTTGCTTTACTCGAGGAGT........................................ 1
.......................................................................................................................................................TCGAGGAGTATGTAGGTCGCCCAC......................... 1
...................................................................................................................................................................TAGGTCGCCCACCCAGTTGTG................ 1
```
