# Supplementary material for: High throughput sequencing reveals novel and abiotic stress-regulated microRNAs in the inflorescences of rice
Source: BMC Plant Biol. 2012 Aug 3;12:132. doi: 10.1186/1471-2229-12-132 (PMC3431262; doi:10.1186/1471-2229-12-132)
Supplement: Additional file 10 — MITEs are rich sources for generating miRNAs. Relative abundance of different classes of TEs in the genome is compared to the proportion of miRNAs that were derived from different classes of TEs. MITEs are apparently enriched in TEs from which miRNAs were derived. [file 1471-2229-12-132-S10.ppt]

## Slide 1
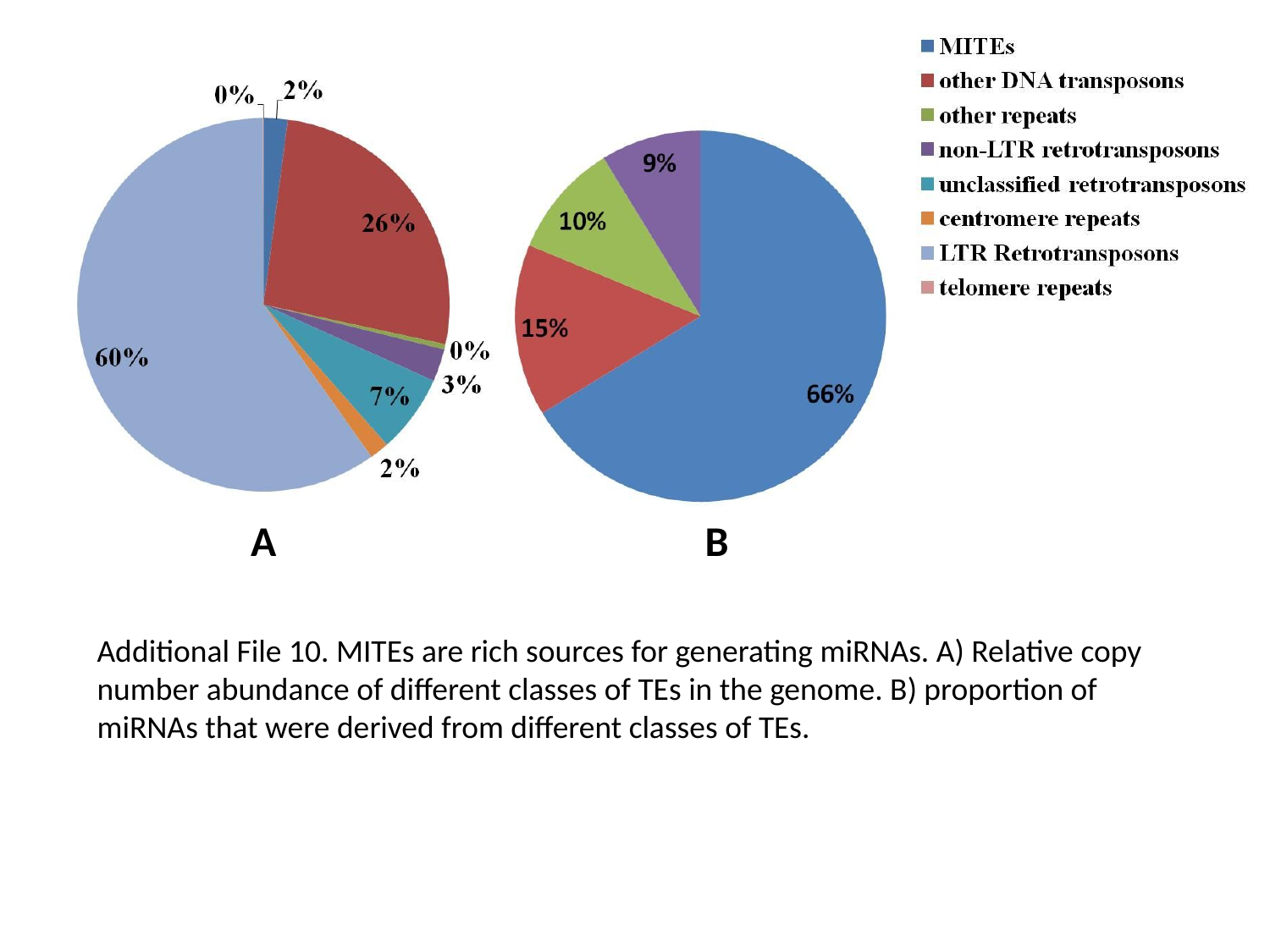

A B
Additional File 10. MITEs are rich sources for generating miRNAs. A) Relative copy number abundance of different classes of TEs in the genome. B) proportion of miRNAs that were derived from different classes of TEs.
